# TFIIH-p52**Δ**C defines a ninth xeroderma pigmentosum complementation–group XP-J and restores TFIIH stability to p8-defective trichothiodystrophy

**DOI:** 10.1172/JCI195732

**Published:** 2025-09-09

**Authors:** Yuka Nakazawa, Lin Ye, Yasuyoshi Oka, Hironobu Morinaga, Kana Kato, Mayuko Shimada, Kotaro Tsukada, Koyo Tsujikawa, Yosuke Nishio, Hiva Fassihi, Shehla Mohammed, Alan R. Lehmann, Tomoo Ogi

**Affiliations:** 1 Department of Molecular Genetics, Center for Neurological Diseases and Cancer, Nagoya University Graduate School of Medicine, Nagoya, Japan.; 2 Department of Genetics, Research Institute of Environmental Medicine (RIeM), Nagoya University, Nagoya, Japan.; 3 National Xeroderma Pigmentosum Service, Department of Photodermatology, St John’s Institute of Dermatology, and; 4 Rare Diseases Centre, Guy’s and St Thomas’ NHS Foundation Trust, London, United Kingdom.; 5 Genome Damage and Stability Centre, University of Sussex, Brighton, United Kingdom.; 6 Department of Human Genetics, Graduate School of Medicine, Nagoya University, Nagoya, Japan.; 7 Center for One Medicine Innovative Translational Research (COMIT), Nagoya University, Nagoya, Japan.; 8 Division of Molecular Physiology and Dynamics, Institute for Glyco-core Research (iGCORE), Tokai National Higher Education and Research System, Nagoya, Japan.; 9 Rare Disease Genome Centre, Nagoya University, Nagoya, Japan

**Keywords:** Cell biology, Dermatology, Genetics, DNA repair, Genetic diseases, Molecular biology

## Abstract

Few drugs are available for rare diseases due to economic disincentives. However, tailored medications for extremely rare disorders (N-of-1) offer a ray of hope. Artificial antisense oligonucleotides (ASOs) are now best known for their use in spinal muscular atrophy (SMA). The success of nusinersen/Spinraza for SMA indicates the potential of ASO therapies for other rare conditions. We propose a strategy to develop N-of-1 ASOs for treating one form of trichothiodystrophy (TTD), a rare condition with multisystem abnormalities and reduced life expectancy, associated with instability and greatly reduced amounts of the DNA-repair/transcription factor TFIIH. The therapeutic targets carry mutations in *GTF2H5*, encoding the TFIIH-p8 subunit. This approach was inspired by the diagnosis and molecular dissection of a xeroderma pigmentosum (XP) case with mutations in *GTF2H4*, encoding the TFIIH-p52 subunit. This is newly classified as a ninth XP complementation–group, XP-J, identified 5 decades after the discovery of the other XP complementation–groups. The p8-p52 interaction is required to support the TFIIH-complex formation, and the patient’s p52 C-terminal truncation results in the complete absence of p8 in TFIIH. However, intriguingly, TFIIH remained stable in vivo, and the patient with XP-J did not exhibit any TTD-features. The aim of our ASO-design is to induce a C-terminal truncation of p52 and we have successfully stabilized TFIIH in p8-deficient cells from patients with TTD-A.

## Abstract 2

This study identifies a potential therapeutic-route for severe trichothiodystrophy and exemplifies how molecular dissection of rare-disease can inform treatment strategies for others, highlighting the translational power of personalised-medicine.

## Graphical abstract

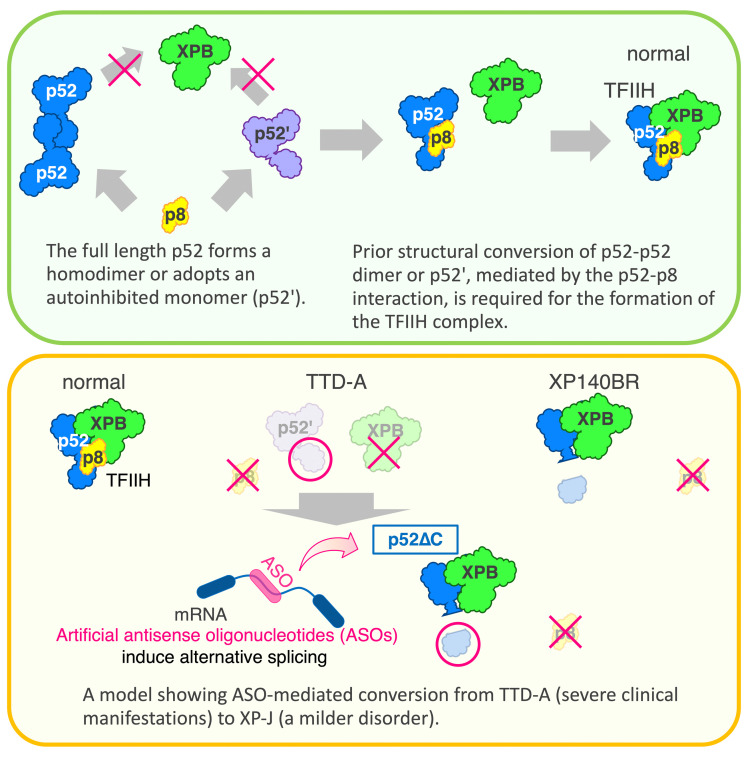

## Introduction

DNA repair and DNA damage response systems (DDR) are essential for maintaining genome integrity and are thus indispensable for life (1, 2). Congenital defects in DDR in humans lead to genome instability syndromes, a group of disorders typically characterized by developmental abnormalities, cancer predisposition, and premature aging (3–6). Many of these disorders are rare, with some classified as extremely rare disorders (N-of-1), defined as having fewer than 100 patients worldwide, or even as N-of-1, in which only a single case has been identified. The development of therapies for rare diseases is innately challenging, particularly due to the limited number of cases, which hampers progress in understanding pathogenesis, and the lack of economic feasibility for such as large-scale screening of small-molecule compounds (7). In light of these difficulties, personalized medicine, particularly therapies based on engineered nucleic acids such as artificial antisense oligonucleotides (ASOs), which are designed as sequence-specific drugs built on a common chemical backbone, is attracting growing interest as a promising alternative (8). Spinraza (nusinersen), an approved 2’-MOE-based ASO for the treatment of spinal muscular atrophy (SMA), is the most successful nucleic acid–based therapeutic to date (9–11). Notably, milasen is known as the first personalized ASO drug for an N-of-1 case (12), and more recently, treatment of a boy with atipeksen has been reported for ataxia-telangiectasia (AT), a DDR-deficiency disorder (13). All of these ASOs function by modulating pre-mRNA splicing (i.e., splicing switching).

Nucleotide excision repair (NER) is the most versatile DNA repair system, responsible for removing a wide range of DNA lesions induced by both endogenous and exogenous sources (14). Xeroderma pigmentosum (XP), Cockayne syndrome (CS), and trichothiodystrophy (TTD) are the 3 best-characterized genodermatoses resulting from NER-deficiency in humans (15, 16). XP shows photosensitivity and a high incidence of skin cancer, with neurological symptoms observed in some complementation groups (17–20). To date, XP has been classified into 8 complementation groups, XP-A–XP-G (21–25), and the variant form XP-V (26), with the corresponding genes identified as *XPA* (27), *ERCC3*/*XPB* (28), *XPC* (29), *ERCC2*/*XPD* (30), *DDB2*/*XPE* (31), *ERCC4*/*XPF* (32), *ERCC5*/*XPG* (33), and *POLH*/*XPV* (34). CS manifests as growth failure and premature aging (35, 36), whereas TTD involves systemic neurodevelopmental abnormalities, typically including sulfur-deficient brittle hair due to aberrant gene expression (37, 38). Mechanistically, NER is divided into 2 subpathways based on the mode of DNA damage recognition: global genome repair (GGR), in which XPC and UV-DDB (XPE) proteins detect DNA damage (39), and transcription-coupled repair (TCR), which is initiated by the stalling of RNA polymerase II (RNAPII) at sites of DNA lesions (4, 5, 40, 41). Following damage recognition, the incision and repair synthesis steps are shared by both NER subpathways. Typical CS cases, with mutations in *CSA* or *CSB*, result in defects in the initiation of TCR (35, 36). In contrast, XP (excluding GGR-deficient XP-C, XP-E, and NER-proficient XP variant) and TTD (excluding nonphotosensitive TTD) involve deficiencies in the damage removal process, resulting in the loss of both GGR and TCR activities (6). Rare mutations in *ERCC1* and *ERCC4*/*XPF*, encoding the NER 5’-endonuclease, may cause complicated features beyond XP, CS, and TTD, including Cerebro-Oculo-Facio-Skeletal Syndrome (COFS) (42), Fanconi anemia (FA) (43, 44), XFE-progeroid syndrome (XFE) (45), XP-CS-FA (44), and *ERCC1*-hepatorenal syndrome (46). Among NER factors, general transcription factor II H (TFIIH), which functions in both transcription and DNA repair, plays a central role in critical steps of the NER process (47–49). These steps include binding to damage recognition factors, unwinding DNA around the lesion, and recruiting XPA and endonucleases, XPF-ERCC1 and XPG, to the damage site. TFIIH consists of 10 subunits, composed of the core complex (p62, p52, p44, p34, and p8) and the CAK complex (MAT1, CDK7, and Cyclin H), with XPB and XPD serving as ATPase and helicase (50). Within the TFIIH complex, only mutations in the genes encoding XPB, XPD, and p8 have been implicated in human disorders, including XP, CS (XP combined with CS), and TTD (15, 16). In the accompanying paper, we describe an XP patient assigned to a newly defined ninth XP complementation group, XP-J, who carries mutations in *GTF2H4*, the gene encoding the p52 subunit of TFIIH (51).

In this study, we investigate the molecular pathogenesis of the patient with XP-J and explain why the mutations that induce truncation of p52 do not lead to TTD or CS phenotypes, despite the expectation that such loss would disrupt the p52-p8 interaction, destabilize TFIIH, and result in typical TTD pathology. Through the analysis, we demonstrate that TFIIH can remain stable even in the absence of p8 when combined with p52 C-terminal truncation. Furthermore, we show that ASOs that induce a p52 C-terminal truncation in cells from patients with TTD lacking p8 (TTD-A), lead to the stabilization of TFIIH. These findings suggest that our designed ASOs may be applicable as a therapeutic strategy for severe TTD-A cases. This approach highlights the promise of ASO-based personalized medicine while also demonstrating that insights from rare disease research can drive therapeutic advances in other disorders.

## Results

### TFIIH is stably formed in patient with XP-J cells expressing C-terminally truncated p52.

**Figure 1:**
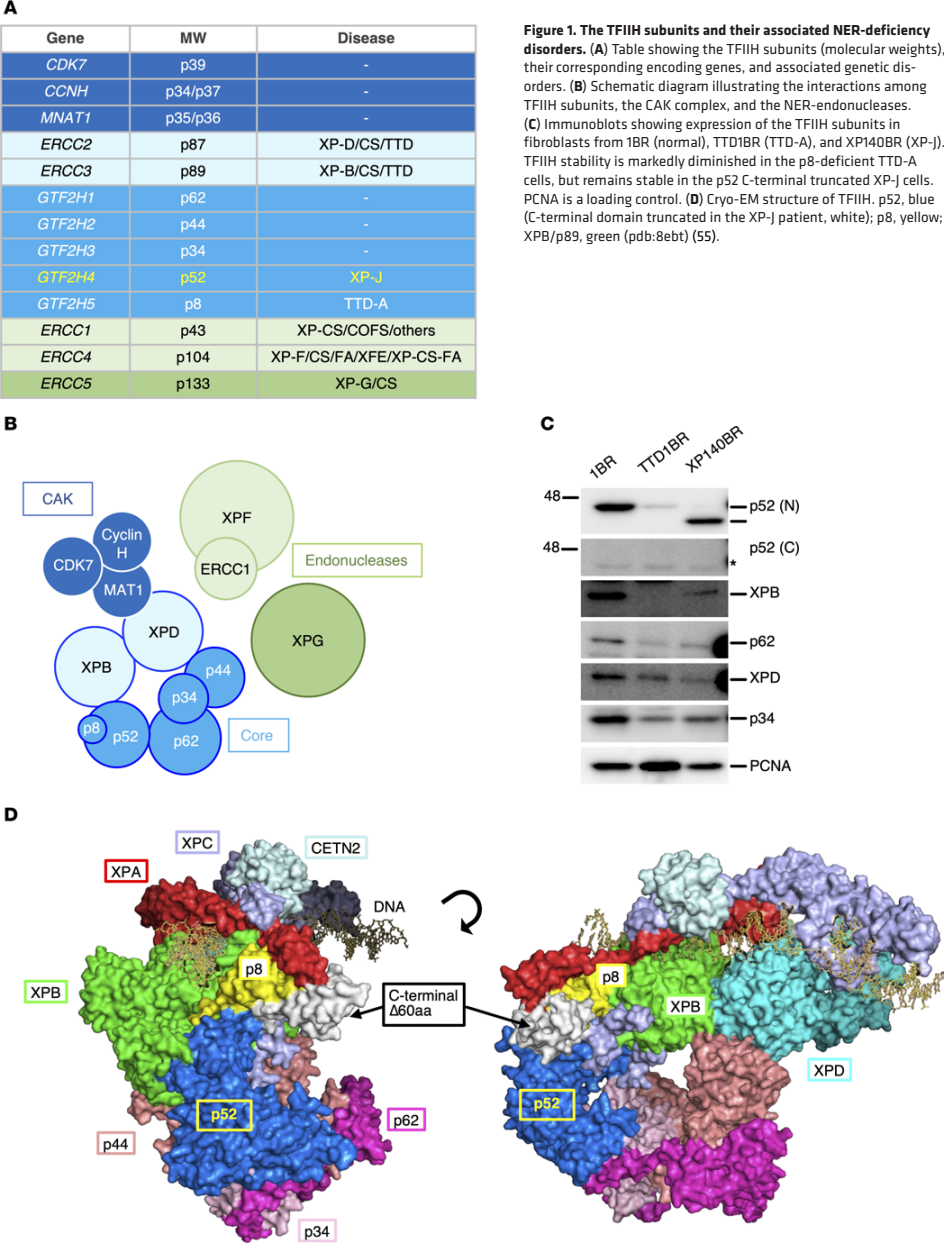
The TFIIH subunits and their associated NER-deficiency disorders. (**A**) Table showing the TFIIH subunits (molecular weights), their corresponding encoding genes, and associated genetic disorders. (**B**) Schematic diagram illustrating the interactions among TFIIH subunits, the CAK complex, and the NER-endonucleases. (**C**) Immunoblots showing expression of the TFIIH subunits in fibroblasts from 1BR (normal), TTD1BR (TTD-A), and XP140BR (XP-J). TFIIH stability is markedly diminished in the p8-deficient TTD-A cells, but remains stable in the p52 C-terminal truncated XP-J cells. PCNA is a loading control. (**D**) Cryo-EM structure of TFIIH. p52, blue (C-terminal domain truncated in the XP-J patient, white); p8, yellow; XPB/p89, green (pdb:8ebt) (55).

Malfunctions in the TFIIH-core complex and its associated factors, XPB/p89 and XPD, are known to cause NER-deficient disorders, XP, CS, and TTD, as well as combined conditions (Figure 1, A and B). Congenital mutations in the TFIIH p8 subunit are known to cause TTD-A (52), with loss-of-function variants in particular leading to the most severe form of the disease (53, 54). In the accompanying paper (51), we reported that the patient with XP-J (XP140BR) intrinsically expresses a pathogenic, C-terminally truncated form of p52 (p52ΔC) and exhibits only XP clinical manifestations without features of the TTD phenotype, despite the missing domain (also known as p8L) being essential for the interaction between p52 and p8 (55).

We presumed that the severity of TTD is attributable to a reduction in the TFIIH abundance and functionality due to complex destabilization, which explains the absence of p8 in severe TTD-A patients. In the cells from the patient with XP-J, the TFIIH complex remains stable in vivo and retains its function even without interaction between p52 and p8. Indeed, immunoblot analysis demonstrated a substantial reduction in the steady-state level of the TFIIH-core complex components, including XPB/p89 and p62, in TTD-A cells, whereas these proteins were slightly reduced but stably expressed in the cells from the patient with XP-J (Figure 1C). The missing p52 C-terminal domain (Figure 1D, highlighted in white) is also structurally important for the recruitment of TFIIH to damaged DNA in chromatin, mediated through its interactions with XPC (purple) and XPA (red) (55).

### TFIIH recruitment to DNA damage is compromised in cells from the patient with XP-J.

**Figure 2:**
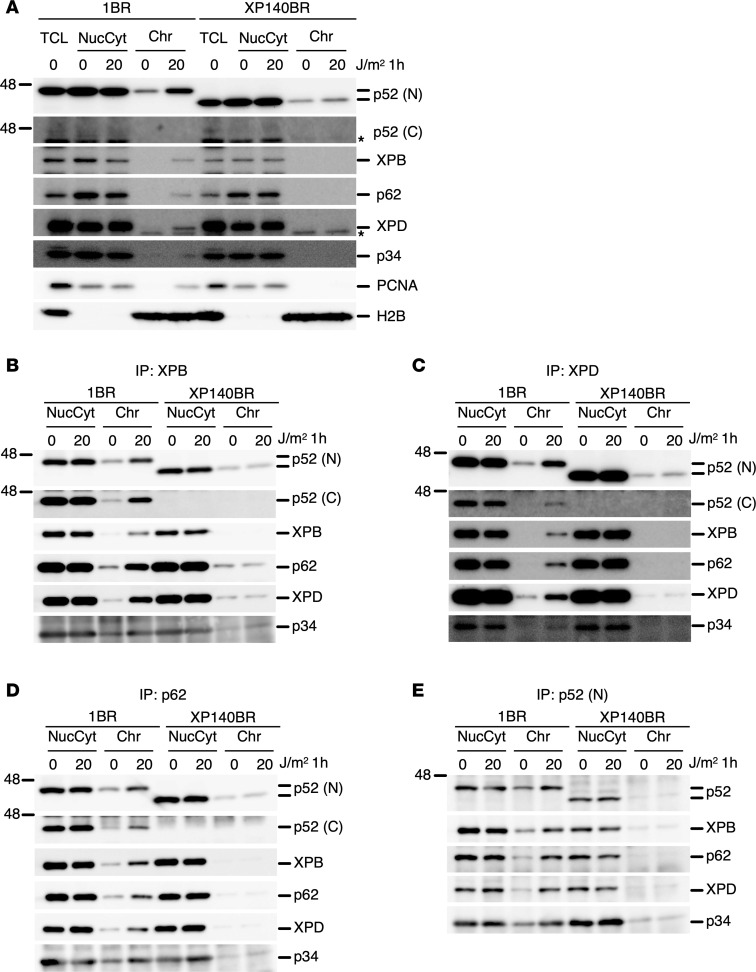
TFIIH recruitment to the chromatin fraction after DNA damage is compromised in XP-J cells. (**A**) TFIIH recruitment to damaged chromatin is only detectable in normal cells (input samples). (**B**–**D**) Immunoprecipitation was performed using the following antibodies: anti-XPB/p89 (**B**), XPD (**C**), p62 (**D**), and p52 (N-terminal) (**E**). TCL, total cell lysate; NucCyt, mixture of nuclear and cytoplasmic fractions; Chr, chromatin fraction. Antibodies used for immunodetection are indicated. The p52 (**C**) antibody recognizes the C-terminal domain of p52. PCNA serves as a loading control, and H2B is a histone protein localized in the chromatin fraction.

NER-deficient cellular phenotypes were observed in the cells from the patient with XP-J (51), attributable to the loss of TFIIH-p52–mediated DNA repair function. To assess the recruitment of TFIIH to DNA damage sites, chromatin coimmunoprecipitation of the TFIIH subunits was performed using extracts from UV-irradiated samples (Figure 2). The p52 C-terminal antibody hardly detects endogenous p52 in whole-cell lysates in normal cells, but the signal becomes detectable after immunoprecipitation. In normal cells, recruitment of all tested TFIIH-core subunits to the chromatin fraction after DNA damage was clearly detectable, whereas in the cells from the patient with XP-J, only the p52ΔC subunit was relocalize (Figure 2A). Chromatin coimmunoprecipitation experiments further confirmed that none of the TFIIH subunits were relocalize to chromatin in UV-irradiated XP-J cells (Figure 2, B–E). This indicates that the recruitment of core-TFIIH to DNA damage via GG-NER is largely compromised when the p52 C-terminal domain is lacking, even though TFIIH subunits have multiple docking interfaces with one another and to XPC. Notably, the total amount of TFIIH complex measured by the XPB/p89 immunoprecipitation was comparable in both normal and cells from the patient with XP-J (Figure 2B). Collectively, the DNA repair deficiency in the patient with XP-J can be solely attributed to the failure of TFIIH translocation after DNA damage, due to the loss of the p52 C-terminal domain, which mediates interactions with other NER factors (55).

### TFIIH p8 is dispensable for stable complex formation and TCR in patient with XP-J.

**Figure 3:**
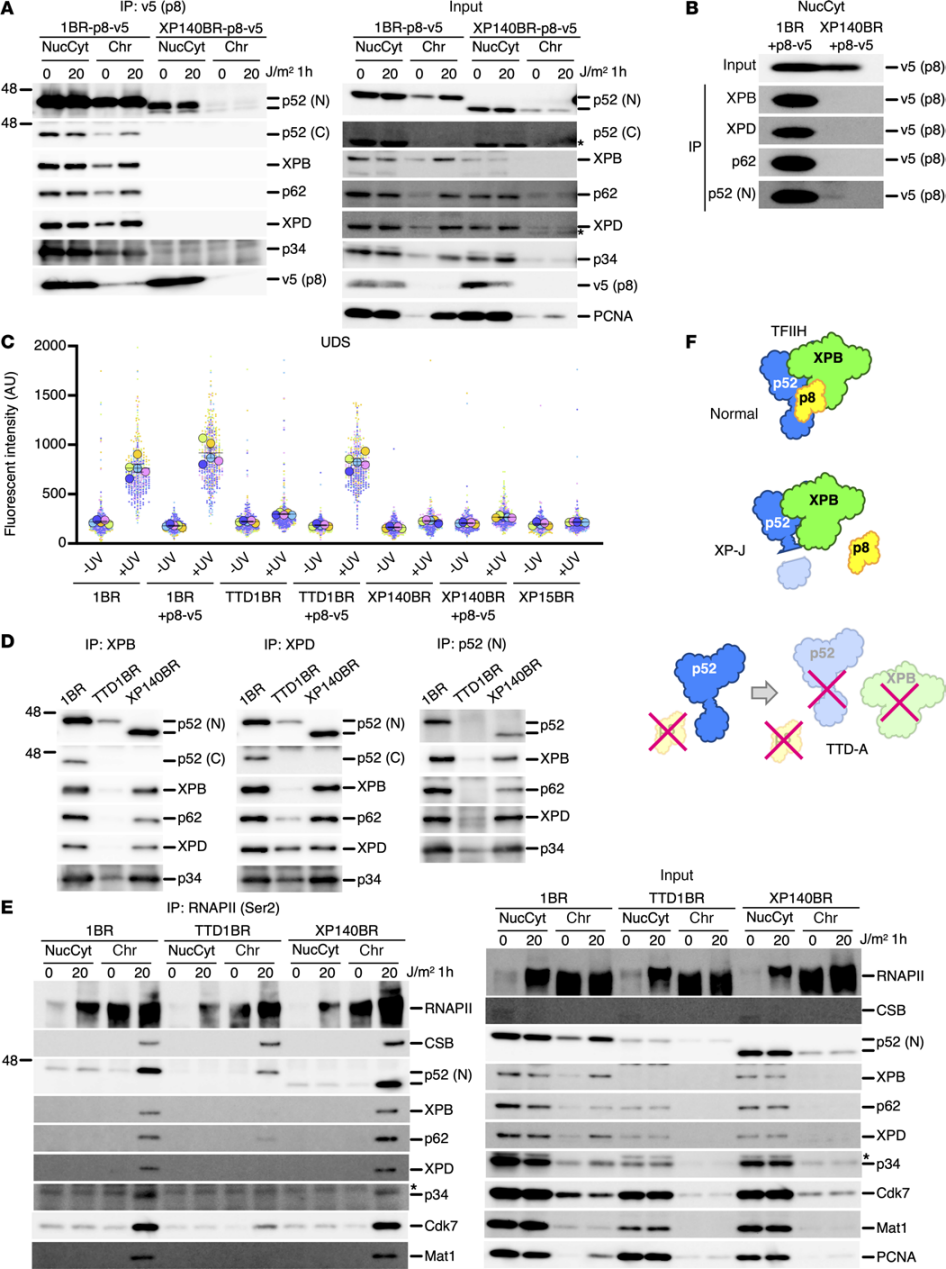
TFIIH-p8 is missing in XP-J cells, but the p52 C-terminal truncation compensates for the complex stability. (**A**) V5-tagged p8 was expressed in cells and immunoprecipitated using an anti-V5 antibody to detect the TFIIH-core complex by immunoblotting. The full-length and the C-terminal truncated form of p52 are indicated. (**B**) Counter coimmunoprecipitation was performed using antibodies against TFIIH-subunits to detect V5-tagged p8. (**C**) V5-tagged p8 does not interfere with DNA repair activity in normal cells and fully rescues p8-deficiency in TTD-A cells. Bars and error bars represent means and SEM, respectively, of experiments (*n* = 5, as indicated by the colored-circles and their corresponding plots). 20J/m^2^ UVC-irradiation. (**D**) TFIIH expression is diminished in p8-deficient TTD-A patient cells (TTD1BR). (**E**) Normal TCR initiation is observed through the recruitment of TFIIH to DNA damage-stalled RNAPII in TTD-A and XP-J cells. Immunoprecipitation of elongating RNAPII was performed using anti-phospho-Ser2 antibodies (3E10). (**F**) Schematic representation of the TFIIH stability in XP-J and TTD-A cells. Asterisks indicate nonspecific products.

Considering the XP-exclusive phenotype without TTD features in the patient with XP-J, we anticipated that the patient’s TFIIH complex would be structurally stable and retain the p8 subunit in vivo, thereby maintaining complex integrity, but lack DNA repair activity. Because the p8 subunit was undetectable by immunoblotting in our hands, we introduced V5-tagged p8 to determine whether it would be incorporated into the TFIIH complex. While V5-p8 immunoprecipitated p52ΔC, which likely exists as free, unfolded protein, no other TFIIH subunits were coimmunoprecipitated, indicating that p8 may not be incorporated into the TFIIH complex in the cells from the patient with XP-J (Figure 3A). Counter coimmunoprecipitation experiments by the TFIIH-core subunits confirmed the lack of p8 in the TFIIH-core complex in the cells from the patient with XP-J (Figure 3B). As measured by UDS, overexpression of V5-tagged p8 did not interfere with DNA repair activity in normal cells, fails to complement the defect in XP140BR, but fully rescued the p8 deficiency in TTD-A cells (Figure 3C). These results indicate that overexpressed V5-tagged p8 retains its repair activity and that the defect in XP140BR extends beyond p8 loss. In cells from patients with TTD-A, the levels of TFIIH subunits that directly interact with p8 (i.e. XPB/p89, p52) were substantially reduced (Figure 1C). To further examine the composition of TFIIH complex, we performed coimmunoprecipitation experiments. In cells from patients with TTD-A, none of the TFIIH subunits were coimmunoprecipitated with XPB/p89 or p52. In contrast, all TFIIH-core components were detected in normal and cells from the patient with XP-J (Figure 3D).

In TCR-deficient disorders, patients with CS exhibit severe systemic abnormalities, and a model for explaining the CS pathogenesis has been proposed in which persistent stalling of RNAPII at DNA lesions eventually leads to the induction of apoptosis (56). In CS, the initiation of TCR is compromised due to defective ubiquitination of stalled RNAPII and impaired recruitment of TFIIH to sites of DNA damage (40). While cells from patients with XP-J and TTD-A also exhibit TCR deficiency, their clinical manifestations differ substantially from those observed in CS. To further investigate the differences in molecular pathogenesis between these disorders, we analyzed the recruitment of TFIIH to DNA damage-stalled RNAPII by chromatin coimmunoprecipitation assays (Figure 3E). In cells from patients with TTD-A, although CSB recruitment to the chromatin fraction and RNAPII ubiquitination proceed normally, there is a marked reduction in the nuclear-localized, unbound TFIIH pool. Furthermore, TFIIH accumulation in chromatin is not detectable by RNAPII immunoprecipitation, indicating that TCR initiation is specifically impaired. This defect is likely due to the destabilization of the TFIIH complex and a reduced total amount of functional TFIIH. In contrast, in the cells from the patient with XP-J, TFIIH recruitment to DNA damage–stalled RNAPII is proficient, suggesting that the absence of p8, due to truncation of the p52 C-terminal domain, affects only the downstream processes of NER, namely the recruitment of NER incision factors such as XPA, followed by repair DNA synthesis.

Collectively, the TFIIH complex remains stable in vivo when the p52 C-terminal domain is truncated (Figure 3F, normal vs. XP-J), whereas the p8 subunit becomes indispensable when the p52 C-terminal domain is present (Figure 3F, normal versus TTD-A). The TCR-defective cellular phenotype in XP-J did not contribute to the development of notable CS or TTD manifestations, as structurally stable TFIIH is abundantly present and the critical processes for removing stalled RNAPII from the chromatin fraction, which is necessary to prevent apoptosis, remain functionally intact (56).

### ASOs designed to induce GTF2H4 alternative splicing promote p52ΔC expression and stabilize the TFIIH complex.

**Figure 4:**
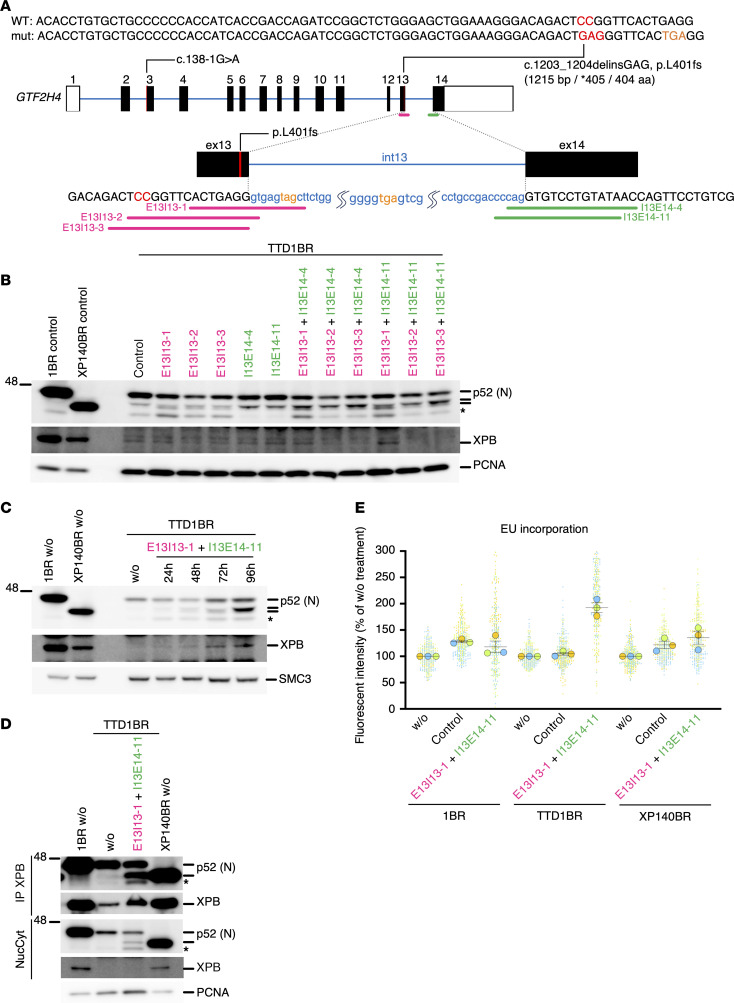
Artificial antisense oligonucleotides (ASOs) designed to induce *GTF2H4* alternative splicing facilitate p52 C-terminal truncation and promote TFIIH stability. (**A**) Schematic representation of the *GTF2H4* gene structure and the design of ASOs to induce intron 13 inclusion, leading to a frameshift and a C-terminal truncation of p52. In-frame stop codons in intron 13 (orange). (**B**) Induction of p52 C-terminal truncation in TTD-A cells treated with ASOs (24 hours). (**C** and **D**) Simultaneous blocking of the 5’ and 3’ splice sites of intron 13 facilitates efficient induction of the p52 truncation and TFIIH stabilization in TTD-A cells. In **D**, immunoprecipitation of XPB/p89 was performed. Asterisks indicate nonspecific products. (**E**) ASO treatment mostly rescues transcription in TTD cells. Transcription activity was measured by EU incorporation. Bars and error bars represent means and SEM, respectively, of experiments (*n* = 3, as indicated by the colored-circles and their corresponding plots). 20J/m^2^ UVC-irradiation. wo, without treatment; control, control-ASO treatment.

Since p8 is dispensable for TFIIH functionality in the patient with XP-J, who exhibits neither CS nor TTD manifestations, we anticipated that inducing p52 C-terminal truncation in p8-deficient cells from patients with TTD-A may facilitate TFIIH stabilization. To achieve this, we designed ASOs to deliberately disrupt proper splice site recognition required for the removal of intron 13, thereby inducing its inclusion in *GTF2H4* mRNA transcripts and mimicking the XP-J patient’s frameshift pathogenic mutation located in its exon 13 (Figure 4A and Table 1). To identify effective ASOs for this purpose, we conducted a screening using a set of systematically designed ASOs, each expected to exert steric blocking effects at either the 5’ or 3’ spliceosome binding sites (Figure 4, A and B). We first introduced individual ASOs separately into p8-null TTD-A cells (TTD1BR) to test their effects on the induction of p52ΔC, using it as a marker for the efficacy of steric-blocking ASOs targeting *GTF2H4* intron 13 splicing (Figure 4B). Twenty-four hours after ASO treatment, several faster-migrating bands appeared in each sample, which may correspond to C-terminal truncated forms of p52 protein. Notably, when 2 ASOs were combined — 1 designed to target the 5’ splice site (pink) and the other targeting the 3’ (green) — the intensity of the faster-migrating p52 products increased. We further tested the stability of TFIIH by measuring the accumulation of XPB/p89. The amount of XPB/p89 increased the most when 2 specific ASOs (E13I13-1 and I13E14-11) were used, indicating that these ASOs function as steric blockers at the 5’ and 3’ spliceosome binding sites of *GTF2H4* intron 13 (Figure 4, A and B).

To further optimize the ASO treatment conditions, we examined the treatment duration on the p52ΔC induction and XPB/p89 accumulation, as a marker of the TFIIH stability, using the most effective ASO pair identified in Figure 4B. The effect was enhanced in a treatment time–dependent manner; marked induction of the truncated forms of p52 protein, and, correspondingly, increased levels of XPB/p89 were both clearly observed after up to 96 hours of treatment (Figure 4C). We further investigated whether any of the observed truncated p52 products correspond to the C-terminal truncated form caused by the pathogenic variants in the patient with XP-J and whether they can properly form the TFIIH complex. Coimmunoprecipitation was performed using XPB/p89 antibodies on samples treated for 96 hours with the ASO pair used in Figure 4C. A distinct product was detected that migrated at the same size as the known truncated form in the cells from the patient with XP-J, and a substantial increase in XPB/p89 levels was also clearly detected (Figure 4D).

To determine whether the ASO-mediated TFIIH stabilization improves transcriptional function, basal transcription levels were measured in the ASO-treated cells. The ASO treatment mostly rescued transcription activity in TTD-A cells (Figure 4E), although complementation experiments to assess transcription recovery by p8 expression in TTD-A cells were not feasible due to the transcriptional impact of viral transduction. Given that prior reports suggest normal TFIIH transcriptional activity without p8 in vitro (57, 58), this issue remains controversial.

Collectively, these data indicate that an induction of p52 C-terminal truncation by ASO treatment facilitates TFIIH complex stability in p8-deficient cells from patients with TTD-A. This suggests that modulating the p52-p8 interaction and promoting TFIIH complex formation may serve as a potential therapeutic strategy for TTD-A cases.

## Discussion

### Molecular pathogenesis of the patient with XP-J.

In this study, we demonstrate how the TFIIH complex remains functional in a newly classified patient with XP-J carrying mutations that result in a C-terminal truncation of p52 (p52ΔC). The truncated p52 C-terminal globular domain (approximately 400–462 aa) is essential for the recruitment of NER factors to TFIIH following DNA damage (Figure 2); thus, as described in the accompanying paper (51), the patient’s typical XP features and NER-deficient cellular phenotype are consistent with this loss. However, the absence of TTD clinical manifestations in the patient with XP-J appears inconsistent with these molecular defects, as the p52 C-terminal domain is also required for its interaction with p8, and a malfunction of p8 is known to cause TTD-A.

Past genetics and molecular biological studies performed in humans and other organisms have revealed that the primary role of p8 is to maintain stability of the TFIIH complex, in addition to support DNA repair via recruitment of NER factors (52). Loss of p8 leads to a reduced level of the TFIIH complex due to destabilization and results in NER defects in cells from patients with TTD-A, while transcriptional activity measured in vitro appears to be normal (57, 58). In the cells from the patient with XP-J, the steady-state level of TFIIH, especially the XPB/p89 subunit, was slightly reduced, but not to the extent observed in cells from patients with TTD-A (Figure 1C). The p8 subunit was not detectable from the TFIIH complex in the XP-J cells overexpressing V5-tagged p8 (Figure 3, A and B), suggesting that p8 may be dispensable for normal fetal development when combined with p52ΔC, despite the absence of p8 alone being lethal in mice (59), and causing a devastating phenotype in humans, as seen in the most severe form of TTD-A (53, 54). On the other hand, a reverse relationship was observed in *Drosophila*, where the same gene combination exhibited cross complementation: embryonic lethality of *mrn*/*mrn* (*marionette*: mutant fly displays phenocopying features of human TTD including, neurological abnormalities, brittle bristles, cuticle defects, and UV hypersensitivity) animals caused by a homozygous truncation mutation in p52 (pQ255*) was rescued by overexpression of p8, suggesting an essential role for p8 as a chaperone in the TFIIH complex stability (60). Nevertheless, these observations suggest that the molecular structural basis for the TFIIH stability involving p8 is not fully identical across species. By contrast, biochemical studies had identified the primary role of p52 as an XPB/p89-associated ATPase stimulator / modulator (61), essential for the opening of lesion-containing DNA in coordination with XPD helicase activity prior to damage incision. Within this function, the second XPB binding domain (aa 305–358, outside of the C-terminal globular domain) in p52 is required for the ATPase stimulation; but, additional roles of the C-terminal domain — despite its loss causing defective NER — remain to be elucidated.

Further TFIIH structural studies in humans have revealed that p52 may facilitate the integration of XPB/p89 into the rest of the complex and that its C-terminal domain is crucial for forming a heterodimer with p8 (55, 62, 63). Through this interaction, p52 and p8 form a module with XPB/p89 to constitute a subcomplex. Thus, the loss of either subunit compromises the integrity of the assembly. Importantly, these structural studies were conducted on the intact TFIIH complex, and, therefore, do not represent the structures of individual subunit monomers as independently functioning units. Detailed substructural analyses of p52 and p8 have been conducted in fungi, demonstrating that the p52-p8 heterodimer assembles independently without requiring other subunits (64, 65). Moreover, a solution structure of human p8 has been elucidated, revealing its presence as a symmetrical homodimer (66). Intriguingly, the yeast p52-p8 heterodimer structure, although lacking the p52 N-terminal domain, resembles that of the human p8 homodimer without sequence homology, which is consistent with observations of the human p52-p8 module structure within the intact TFIIH complex (55, 65, 66). An important question that remains to be elucidated is whether p52 forms a homodimer, which may provide a clue to understanding the molecular pathogenesis of the patient with XP-J.

**Figure 5:**
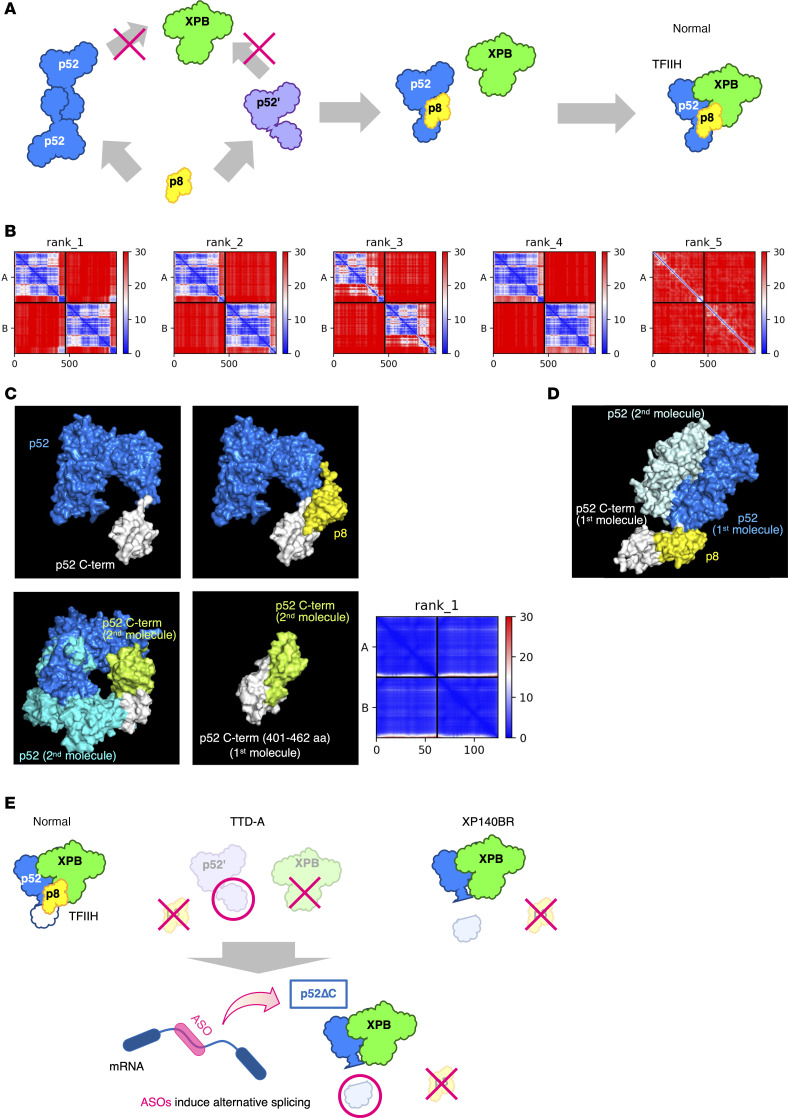
A model for the p52 C-terminal self-inhibitory structure. (**A**) The full length p52 forms a homodimer or adopts an autoinhibited monomer (p52’). Prior structural conversion of p52-p52 dimer or p52’, mediated by the p52-p8 interaction, is required for the formation of the TFIIH complex. (**B** and **C**) AlphaFold structure prediction revealed a potential homodimer formation mediated by the C-terminal domain of p52. In the rank1 model, the relative positioning of the C-terminal domains of molecule A and molecule B appears to be fixed (blue) (**B**). p52 monomer, shown in blue (top-left: C-terminal domain, white), and p52-p8 dimer (top-right: p8, yellow) in the cryo-EM TFIIH structure, pdb:8ebt (55). Full-length p52 homodimer structure, with N-terminal steric crashes, predicted in **B** (bottom-left: first p52 molecule, blue; second p52 molecule, N-terminal domain, sky blue/C-terminal domain, yellow-green). p52 C-terminal domain (401-462aa) homodimer structure (bottom-middle: first molecule, white; second molecule, yellow-green), and the relative positioning (bottom-right: fixed positions shown in blue) (**C**). (**D**) Crystal structure of a fungal p52-p8 (pdb: 6trs) (64). C-terminal domain of the second p52 molecule lacks electron density. (**E**) A model showing ASO-mediated conversion from TTD-A (severe clinical manifestations) to XP-J (a milder disorder).

We hypothesize that full-length p52 may form a homodimer (p52-p52) or adopt an autoinhibited monomer (p52’) conformation in vivo, either of which may represent an inappropriate structure for assembly into the functional TFIIH complex (Figure 5A). To prevent this, p8 is thought to function by resolving such conformations through heterodimerization with the C-terminal domain of p52. To further investigate whether human full-length p52 can form a homodimer, we employed AlphaFold-based structural predictions (67). Although most of the predicted models suggested that the dimeric configuration is unstable, one model revealed a potentially plausible homodimer mediated through interactions at the C-terminal domain (Figure 5B-rank1). However, this prediction is still not sufficient to establish physiological relevance, as the spatial relationship between the 2 N-terminal regions is not well defined, and the homodimeric model exhibits steric clashes in the N-terminal region (Figure 5C, bottom left). Nevertheless, given that the p8-p52 C-terminal domain heterodimer is structurally indistinguishable from the p8-p8 homodimer (55), and that an additional structural prediction of a p52 C-terminal domain (401–462 aa only) homodimer showed a fixed spatial arrangement between the monomers (Figure 5C, bottom middle and bottom right), it is reasonable to assume that p52 is also capable of forming a homodimer via its C-terminal domain in vivo. Notably, in the crystal structure of the fungal p52-p8 complex, 2 p52 molecules in the asymmetric unit formed a homodimer through their N-terminal regions. The C-terminal domain of the first p52 molecule was stably engaged with p8, whereas that of the second p52 was not observed in the electron density map (Figure 5D), suggesting structural disorder or conformational flexibility (64).

Collectively, these observations and considerations support the model that p52 adopts alternative conformations, either as a homodimer or an autoinhibited monomer, that are incompatible with assembly into the functional TFIIH complex (Figure 5A). Through this intrinsic mechanism, the p52-p8 module regulates its incorporation into TFIIH and modulates the steady state level of the complex. Since the C-terminal domain of p52 is crucial for the formation of these alternative structures, and p8 facilitates their resolution, the absence of this domain in the patient with XP-J renders p8 dispensable for stable TFIIH complex formation. We suggest that this explains the lack of marked TTD features in the patient (Figure 3F).

### Molecular basis of TTD phenotypes independent of DNA repair deficiency.

The absence of characteristic TTD features, such as brittle hair, nail abnormalities, and ichthyosis, in the patient with XP-J provides further evidence that these phenotypes are not caused by DNA repair deficiency, but instead result from instability of components involved in gene expression (68). This aligns with previous observations that mutations affecting the structural stability of TFIIH, as well as other transcriptional regulators (e.g., TFIIE and splicing factors such as MPLKIP, DBR1, and RNF113A) or translational components (e.g., aminoacyl tRNA synthetases, xARS), lead to incomplete terminal differentiation and typical TTD symptoms independent of DNA repair (69). Notably, the XP-J p52ΔC mutation does not destabilize TFIIH, consistent with the absence of TTD manifestations. These findings reinforce the model that protein instability in the gene expression machineries underlies TTD features and suggest that additional, as yet unidentified, mutations in the TFIIH subunits may give rise to phenotypes that do not conform to the classic XP, CS, or TTD classifications.

### A proof of concept for TTD-A therapeutics using ASOs.

Modulation of gene expression through ASOs is emerging as a promising therapeutic strategy for genetic disorders, including those with limited patient populations (70). A well-known example is ‘*nusinersen*’, an ASO approved for the treatment of SMA, which modulates *SMN2* splicing to restore functional SMN protein (9–11). Remarkably, despite the genetic heterogeneity of SMA, a single ASO design is applicable across many patients, highlighting the therapeutic value of targeting shared downstream mechanisms (71). This concept is particularly relevant for N-of-1 disorders, where traditional drug development is often infeasible.

In this study, we demonstrate a proof of concept for ASO-based therapy in TTD-A, the rarest TTD subtype caused by mutations in *GTF2H5*/*TTDA* (38). Our strategy leverages the observation from a patient with XP-J carrying a TFIIH-p52ΔC mutation who, despite lacking p8 in TFIIH, did not exhibit the TTD phenotype (51). This finding suggests that p52ΔC stabilizes TFIIH independently of p8. Based on this insight, we designed ASOs to induce a similar p52 C-terminal truncation by altering *GTF2H4* splicing, thereby functionally compensating for the loss of p8 in TTD-A cells. ASO-treated cells showed partial recovery of TFIIH protein levels and transcriptional function (Figure 4), supporting the feasibility of this compensatory approach (Figure 5E).

Our findings suggest a broader therapeutic concept: restoration of multiprotein complex stability via modulation of a compensatory subunit. This mechanism, akin to extragenic suppression observed in model organisms, is rarely exploited in human therapeutics. The potential to generalize this strategy to other complexes could open new avenues for treating disorders caused by complex instability.

Given that TTD is a systemic disorder caused by transcriptional defects, prospective targets for ASO therapy include the nervous systems, as well as the skin and various visceral organs, where clinical manifestations are commonly observed. ASO delivery must target a wide range of organs and cell types; therefore, therapeutic intervention would likely focus on those tissues where clinical benefit can be most readily achieved. To date, intrathecal injection has been the primary method for delivering ASOs in diseases with neurodegenerative symptoms. In contrast, systemic delivery via intravenous injection has been employed in Duchenne muscular dystrophy (DMD) (eteplirsen), while subcutaneous injection is used in transthyretin amyloidosis (inotersen) (8). If ASO therapy is to be considered for TTD-A, the choice of target organs and delivery routes will need to be evaluated based on safety, efficacy, and delivery efficiency.

In conclusion, our study illustrates how mechanistic insights from a single rare case can inform therapeutic development, underscoring the scientific and clinical value of studying ultra-rare diseases. Personalized strategies, including ASOs targeting shared functional vulnerabilities rather than specific mutations, may offer viable treatments even for disorders with minimal patient numbers.

## Methods

### Sex as a biological variable.

We examined one female case with a very rare autosomal recessive genodermatosis in this study. Sex was not considered as a biological variable.

### Human studies.

The patient with XP-J and the parent’s samples were obtained with a local ethical approval from the Ethics Committee for Human Genome Studies at the Research Institute of Environmental Medicine (RIeM), Nagoya University. See below for details in Study approval.

### Cell lines and culture.

The following cell lines were used in this study: 1BR (normal human primary fibroblasts), XP140BR (primary fibroblasts from the XP-J patient), and TTD1BR (primary fibroblasts from a TTD-A patient) (51, 56, 72–75). All cells were maintained in DMEM (WAKO) supplemented with 10% FCS (Invitrogen) and antibiotics, unless otherwise noted.

### Generation of V5-tagged p8 expressing cells.

Details have been described previously (56). Human *GTF2H5* cDNA was cloned in-frame with a sequence encoding a C-terminal V5 tag into the pLenti6 vector (Invitrogen) to generate pLenti6/*GTF2H5*-V5. Lentivirus particles were produced in HEK293FT cells transfected with the *GTF2H5*-encoding plasmid and ViraPower Packaging Mix (Invitrogen), which includes pLP1, pLP2, and pLP/VSVG, using Lipofectamine 2000 (Invitrogen). Viral supernatants were collected 48 hours after transfection and concentrated using PEG-it Virus Precipitation Solution (System Biosciences). The resulting viral particles were used to infect the indicated primary fibroblast cells.

### Coimmunoprecipitation.

Details were described previously (40, 56). 1BR (normal) and the XP-J (XP140BR), as well as TTD-A (TTD1BR) patient-derived primary fibroblasts were UV irradiated (20 J/m^2^) and incubated for 1 hour. Chromatin (Chr) and the mixture of nuclear/cytoplasm (NucCyt) fractions were prepared as described previously (40). Coimmunoprecipitation (co-IP) was performed at 4°C for 2 hour incubation with 1 mL EB buffer prepared from 4 x 10^6^ cells and 2 μg of indicated antibodies. Complex were precipitated by additional incubation with 20 μL protein A or G agarose beads (Millipore) for 2 hours at 4°C. After washing with high salt buffer (50 mM Tris HCl pH 7.5, 300 mM NaCl, 0.5% NP-40, 2 mM MgCl2), the beads were resuspended in SDS buffer.

### Immunoblotting and antibodies.

Cell lysates and co-IP samples were resolved by SDS-PAGE (5%–20% gradient gels), and proteins were transferred to PVDF membranes for immunodetection. The following antibodies were used in this study: mouse monoclonal antibody to p52 (A10, Santa Cruz Biotechnology), mouse monoclonal antibody to p52 (A2, Santa Cruz Biotechnology), rabbit polyclonal antibody to p52 (A8425, ABclonal), mouse monoclonal antibody to p89 (G10, Santa Cruz Biotechnology), rabbit polyclonal antibody to p89 (A301−337A, Bethyl Laboratories), mouse monoclonal antibody to XPD (G2, Santa Cruz Biotechnology), rabbit polyclonal antibody to XPD (A303-658A, Bethyl Laboratories), mouse monoclonal antibody to p62 (G10, Santa Cruz Biotechnology), rabbit polyclonal antibody to p62 (A303-515A, Bethyl Laboratories), mouse monoclonal antibody to XPG (8H7, Santa Cruz Biotechnology), rabbit polyclonal antibody to p34 (A7188, ABclonal), rat monoclonal antibody to RPB1-phospho-Ser2-CTD (3E10, Millipore), rabbit polyclonal antibody to RPB1-phospho-Ser2-CTD (ab5095, abcam), mouse monoclonal antibody to cdk7 (MO1, Cell Signaling Technology), mouse monoclonal antibody to CSB (E6, Santa Cruz Biotechnology), mouse monoclonal antibody to mat1 (F6, Santa Cruz Biotechnology), rabbit polyclonal antibody to V5-tag (PM003, MBL), mouse monoclonal antibody to V5-tag (1H6, MBL), mouse monoclonal antibody to PCNA (PC10, invitogen), rabbit polyclonal antibody to H2B (ab1790, abcam), and rabbit polyclonal antibody to SMC3 (A300-060A, Bethyl Laboratories).

### Unscheduled DNA synthesis assay.

Details have been described previously (72, 74). Unscheduled DNA synthesis (UDS) was measured using a fluorescence-based ethynyldeoxyuridine (EdU) incorporation assay. Cells were plated in plastic 96-well plates 24 hours prior to the experiments. UV-irradiated (20 J/m^2^ at 254 nm UVC) cells were incubated for 4 hours in medium supplemented with 5 μM 5-ethynyl-2’-deoxyuridine (EdU). Incorporated EdU was detected through fluorescent-azide conjugation (Click-chemistry), and nuclear fluorescence imaging and data processing was automated using the CX7 Imaging System (Thermo Scientific).

### Design of the antisense oligo nucleotides.

Sequences of the antisense oligonucleotides (ASOs) used in this study are listed in Table 1. ASOs were purchased from Ajinomoto Bio-Pharma Services and Hokkaido System Science. ASOs were designed based on the following principal criteria: 17 bp in total length; LNA-modifications (DNA or RNA) were introduced every other nucleotide; in RNA-oligos, the modifications were introduced in other than uridine nucleotides. Off-target profiles of the ASOs were calculated by GGGenome with the following criteria (https://gggenome.dbcls.jp/en/hg19/): database, human genome GRCh37/hg19; max number of mismatches/gaps, 0; search for both strands. The off-targets in genic regions were summarized.

### Transfection of ASOs.

Details were described previously (76). ASOs were transfected using Lipofectamine 2000 or 3000 (ThermoFisher). Cells were seeded in plastic 12 well plates or 15 cm^2^ dishes and cultured in DMEM (10% FBS, without antibiotics) overnight. Transfection reagents were prepared in OptiMEM (ThermoFisher) mixed with Lipofectamine 2000 (1: 500 dilution) or 3000 (1:350 dilution) and ASOs (final concentration 40 nM), followed by dilution in 1 ml or 20 ml DMEM (10% FBS, without antibiotics). The culture media were replaced by the transfection reagents mixture.

### Transcription assay by EU-incorporation.

Details have been described previously (73, 74). Cells were plated in plastic 96-well plates 24 hours prior to the experiments. Transcription level was measured using a fluorescence-based ethynyluridine (EU) incorporation assay. Cells were incubated for 2 hours in medium supplemented with 100 μM 5-ethynyluridine. Incorporated EU was detected through fluorescent-azide conjugation and measured using the CX7 Imaging System.

### p52 dimer structure prediction.

The 3-dimensional structures of the full-length (1–462 aa) and C-terminal (401–462 aa) p52-p52 homodimer were predicted using AlphaFold2 implemented via ColabFold (67, 77). The following parameters were used: sequence search method = MMseqs2, model type = AlphaFold2-multimer-v3, number_of_recycles = 20, number of models = 5, use_amber = true (structure relaxation enabled), use_templates = true, and max-msa = 32:64. All other parameters were set to default. Structural models were ranked based on the predicted aligned error (PAE) and pLDDT scores.

### Statistics.

All experiments were performed at least in triplicate.

### Study approval.

This study was approved by the Ethics Committee for Human Genome Studies at the Research Institute of Environmental Medicine (RIeM), Nagoya University (approval no. 337-42). Written informed consent was obtained prior to participation. Photographs of the patient were taken with separate consent for their use, and records of informed consent have been retained.

### Data availability.

All data and materials used in this study are available from the corresponding author upon reasonable request. Supporting data values for Figure 3C and Figure 4E are available in the Supplemental Data Values file in Microsoft Excel format (supplemental material available online with this article; https://doi.org/10.1172/JCI195732DS1).

## Author contributions

TO designed the study and wrote the manuscript. Y Nakazawa, LY, and YO are cofirst authors, with the order reflecting Y Nakazawa’s key role in the project, followed by LY and YO according to their respective contributions to the experimental work. Y Nakazawa, LY, YO, HM, KK, MS, and K Tsukada performed the experiments. K Tsujikawa, Y Nishio, HF, SM, ARL, and TO analyzed the data. All authors reviewed the manuscript.

## Funding support

- Japan Agency for Medical Research and Development (AMED) under grant numbers JP23ek0109678, JP23ek0109617, JP24ek0109760 and JP24ek0109765 to TO.
- Grants in Aid for Scientific Research KAKENHI from the Japan Society for the Promotion of Science (JSPS) (JP 23H00516) to TO.
- Science Research Grants from The Uehara Memorial Foundation to TO.
- Visionary Research Grants from Takeda Science Foundation to TO.
- KAKENHI JSPS grant number JP24K02223 to Y Nakazawa.
- JST FOREST Program (JPMJFR221E) at the Japan Science and Technology Agency to Y Nakazawa.
- Takeda Science Foundation to Y Nakazawa.

## Supplementary Material

Unedited blot and gel images

Supporting data values

## Figures and Tables

**Table 1:**
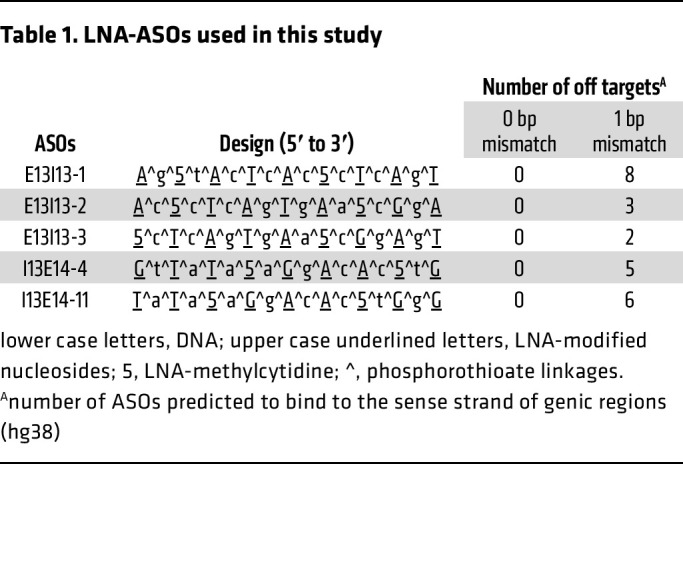
LNA-ASOs used in this study

## Version 1 09/09/2025

In-Press Preview

## Version 2 11/17/2025

Electronic publication
