# Supplementary material for: TFIIH-p52ΔC defines a ninth xeroderma pigmentosum complementation–group XP-J and restores TFIIH stability to p8-defective trichothiodystrophy
Source: J Clin Invest. 2025 Sep 9;135(22):e195732. doi: 10.1172/JCI195732 (PMC12618060; doi:10.1172/JCI195732)
Supplement: Unedited blot and gel images [file jci-135-195732-s225.pdf]

Fig1 c

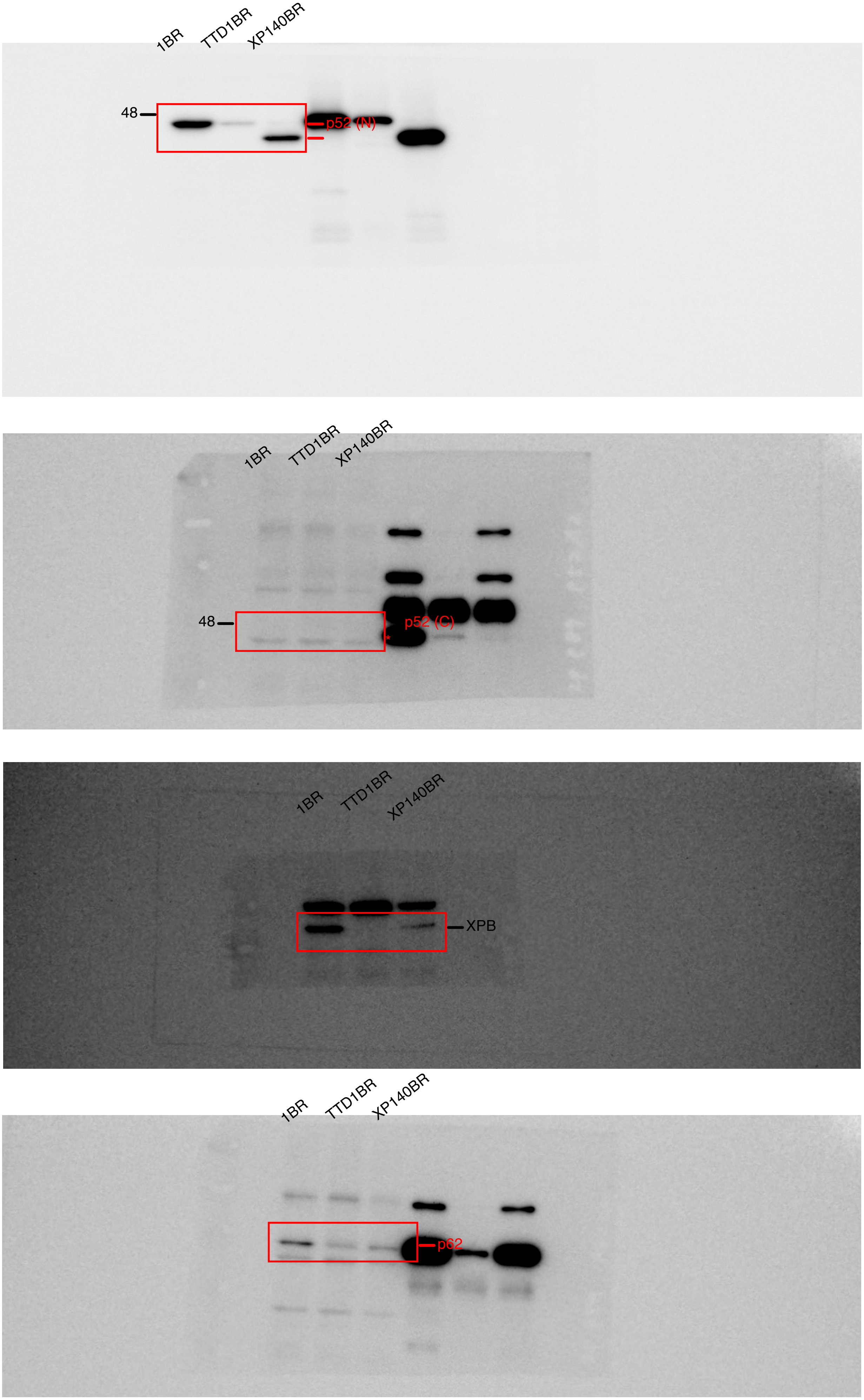
1BR
TTD1BR
XP140BR
48
p52 (N)
1BR
TTD1BR
XP140BR
48
p52 (C)
*
1BR
TTD1BR
XP140BR
XPB
1BR
TTD1BR
XP140BR
p62

Fig1 c

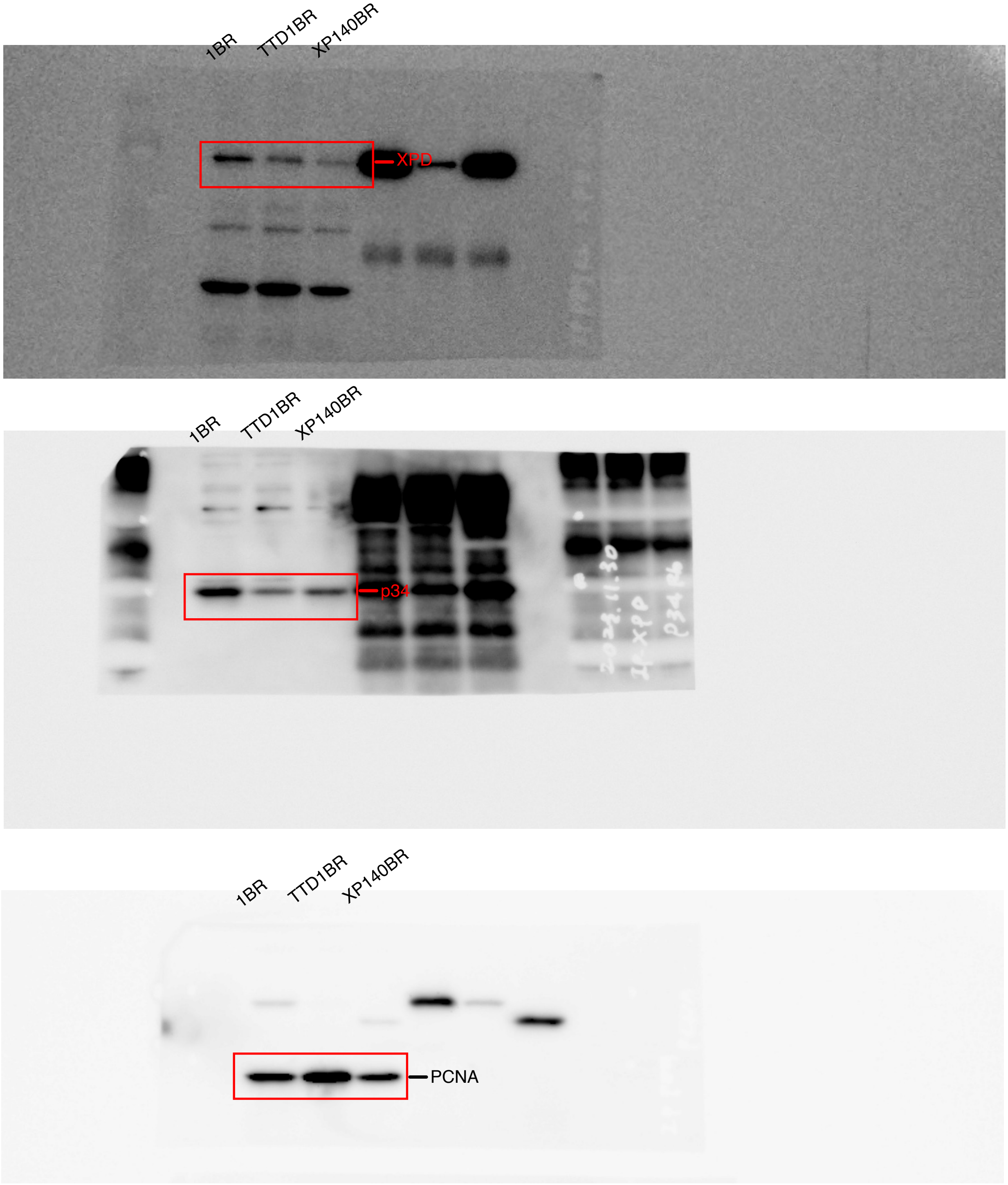

1BR
TTD1BR
XP140BR
XPD
1BR
TTD1BR
XP140BR
p34
1BR
TTD1BR
XP140BR
PCNA

Fig2 a

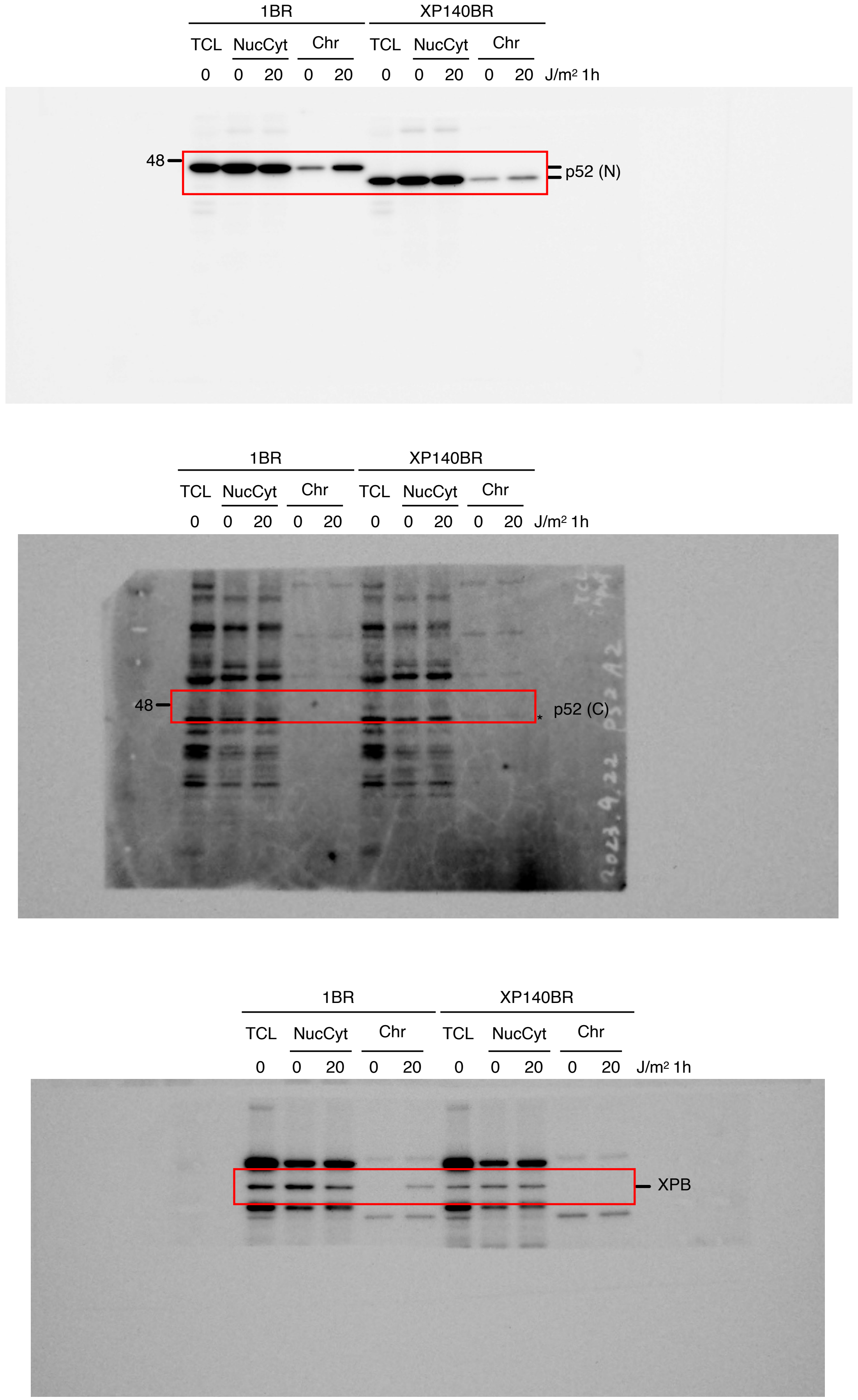
1BR
XP140BR
TCL
NucCyt
Chr
TCL
NucCyt
Chr
0 0 20 0 20 0 0 20 0 20 J/m² 1h
48
p52 (N)
1BR
XP140BR
TCL
NucCyt
Chr
TCL
NucCyt
Chr
0 0 20 0 20 0 0 20 0 20 J/m² 1h
48
*
p52 (C)
1BR
XP140BR
TCL
NucCyt
Chr
TCL
NucCyt
Chr
0 0 20 0 20 0 0 20 0 20 J/m² 1h
XPB

Fig2 a

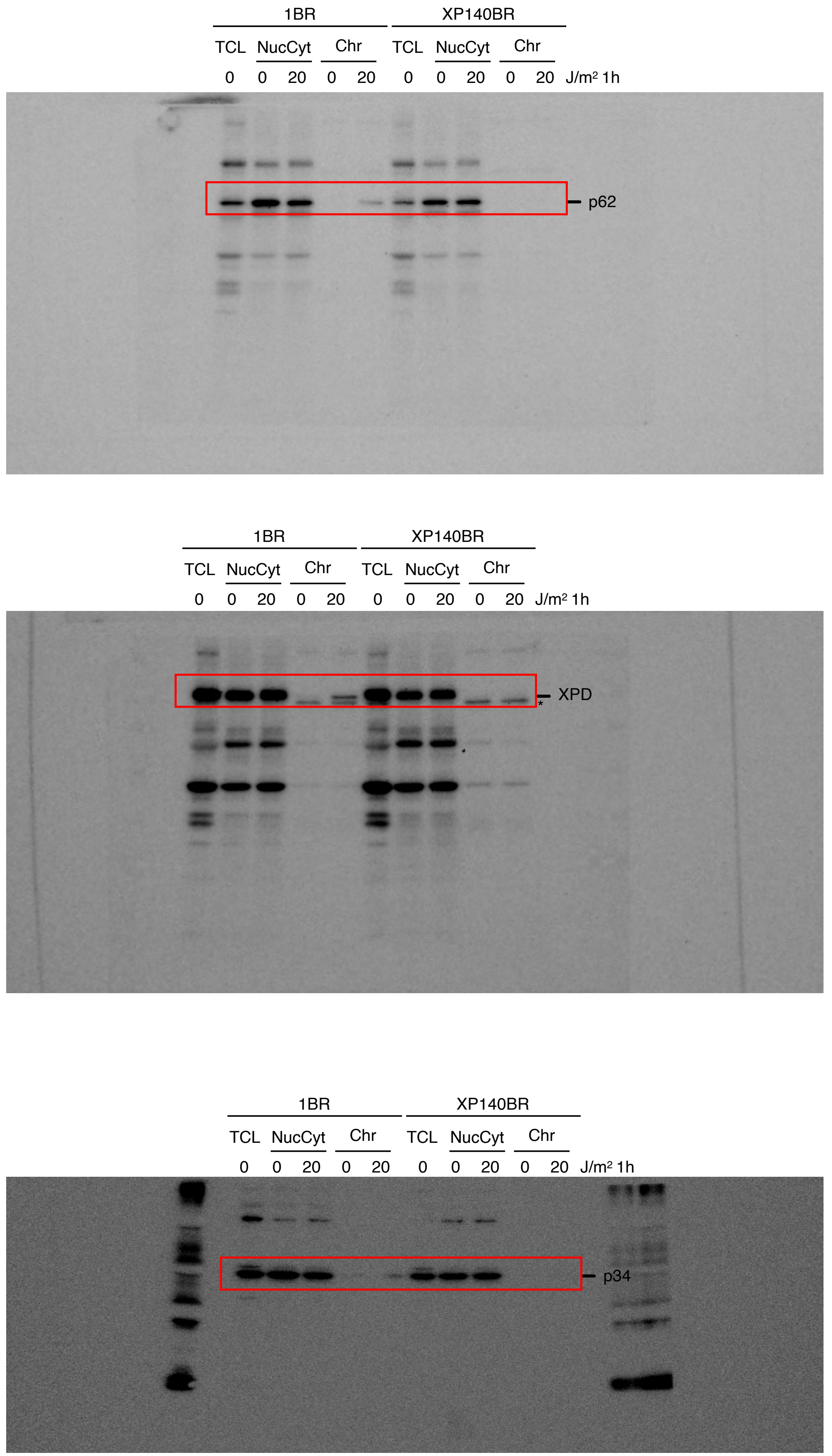

1BR
XP140BR
TCL NucCyt Chr TCL NucCyt Chr
0 0 20 0 20 0 0 20 0 20 J/m² 1h
1BR
XP140BR
TCL NucCyt Chr TCL NucCyt Chr
0 0 20 0 20 0 0 20 0 20 J/m² 1h

Fig2 a

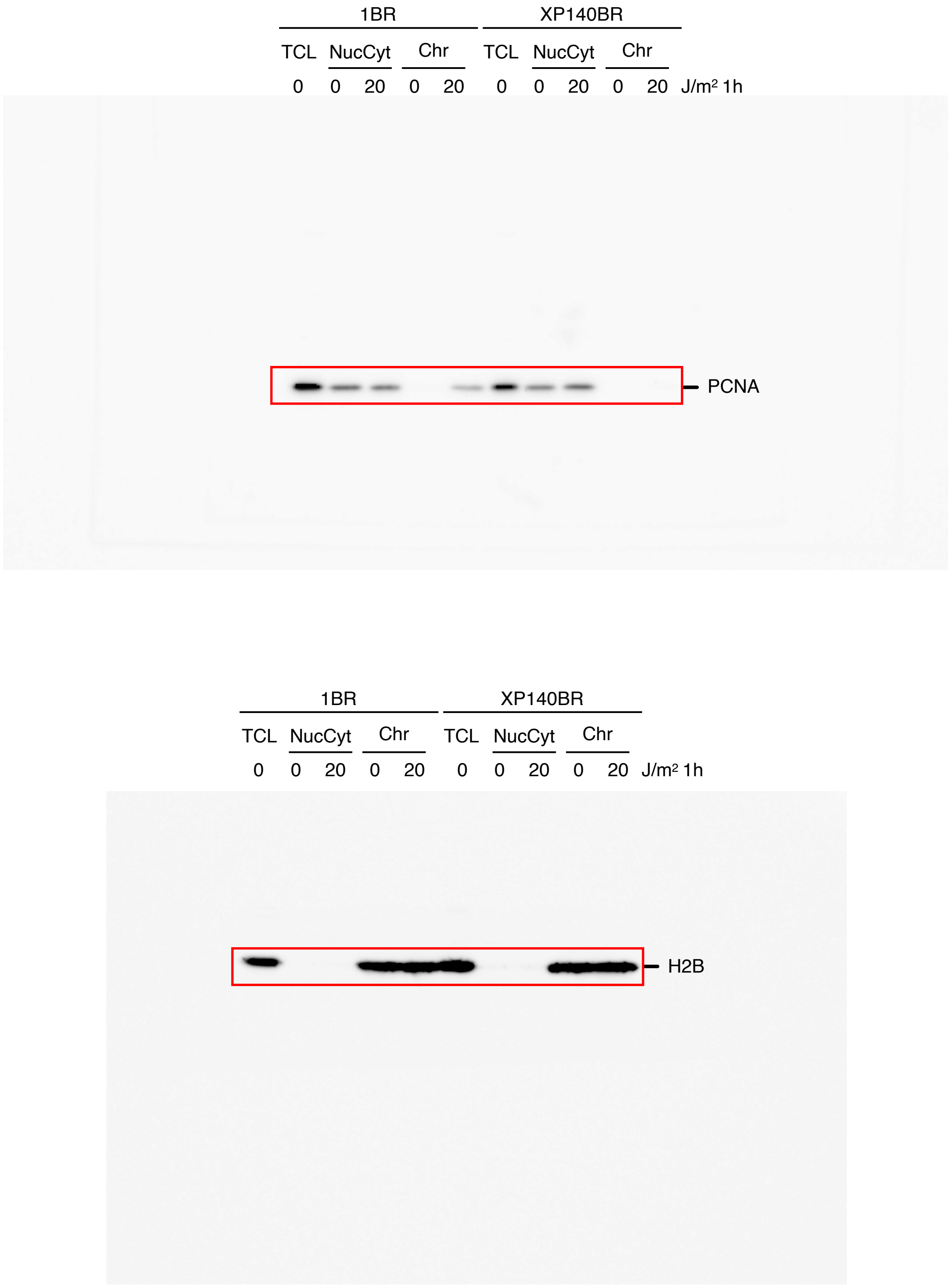
1BR
XP140BR
TCL
NucCyt
Chr
TCL
NucCyt
Chr
0 0 20 0 20 0 0 20 0 20 J/m2 1h
PCNA
1BR
XP140BR
TCL
NucCyt
Chr
TCL
NucCyt
Chr
0 0 20 0 20 0 0 20 0 20 J/m2 1h
H2B

Fig2 b

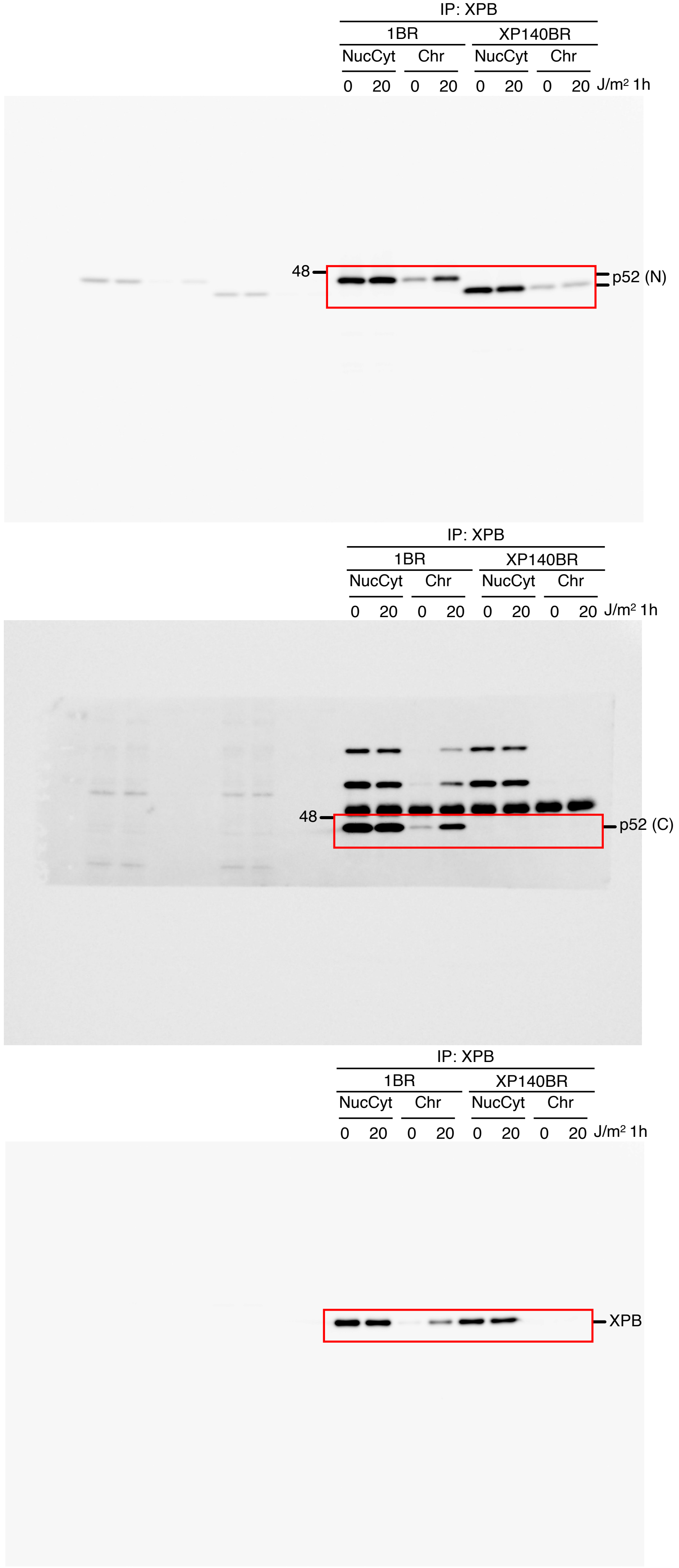
IP: XPB
1BR
XP140BR
NucCyt
Chr
NucCyt
Chr
0
20
0
20
0
20
0
20
J/m² 1h
48
p52 (N)
IP: XPB
1BR
XP140BR
NucCyt
Chr
NucCyt
Chr
0
20
0
20
0
20
0
20
J/m² 1h
48
p52 (C)
IP: XPB
1BR
XP140BR
NucCyt
Chr
NucCyt
Chr
0
20
0
20
0
20
0
20
J/m² 1h
XPB

Fig2 b

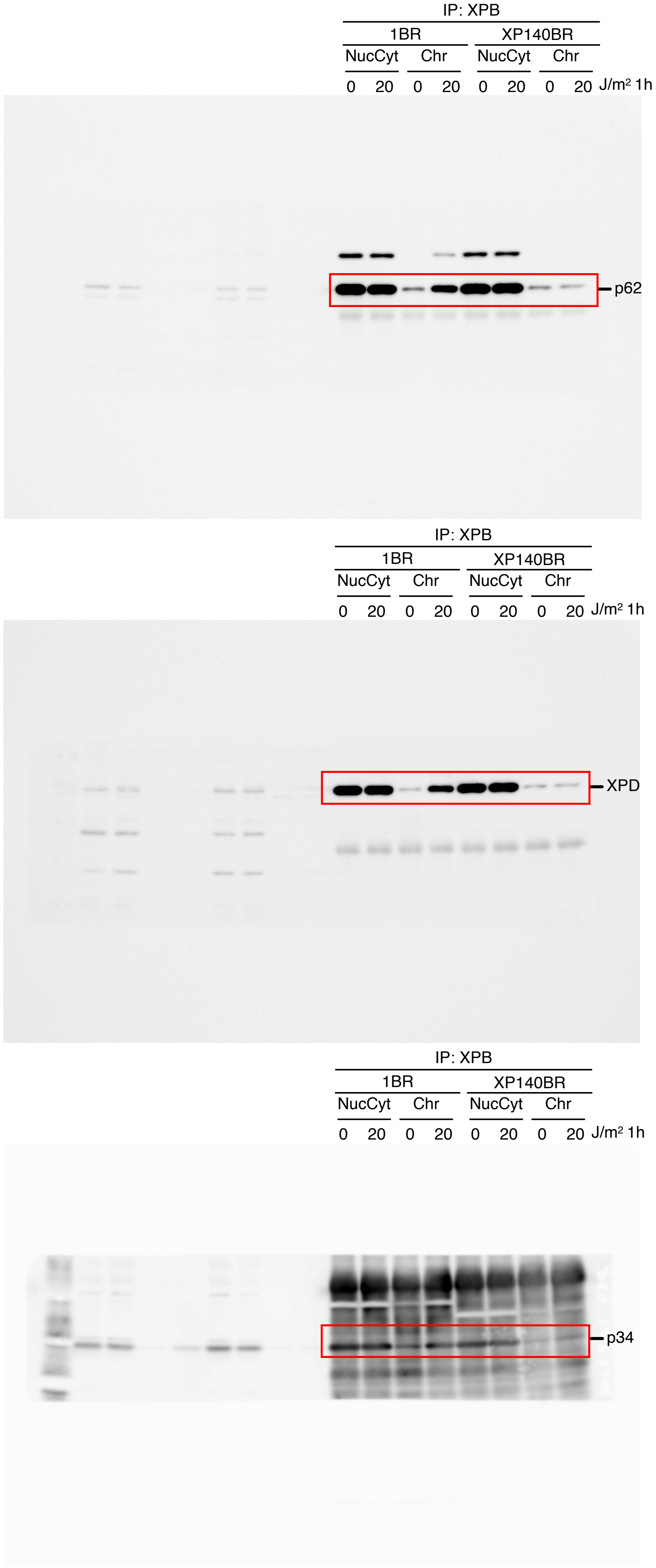
IP: XPB
1BR
XP140BR
NucCyt
Chr
NucCyt
Chr
0
20
0
20
0
20
0
20
J/m² 1h
p62
IP: XPB
1BR
XP140BR
NucCyt
Chr
NucCyt
Chr
0
20
0
20
0
20
0
20
J/m² 1h
XPD
IP: XPB
1BR
XP140BR
NucCyt
Chr
NucCyt
Chr
0
20
0
20
0
20
0
20
J/m² 1h
p34

Fig2 c

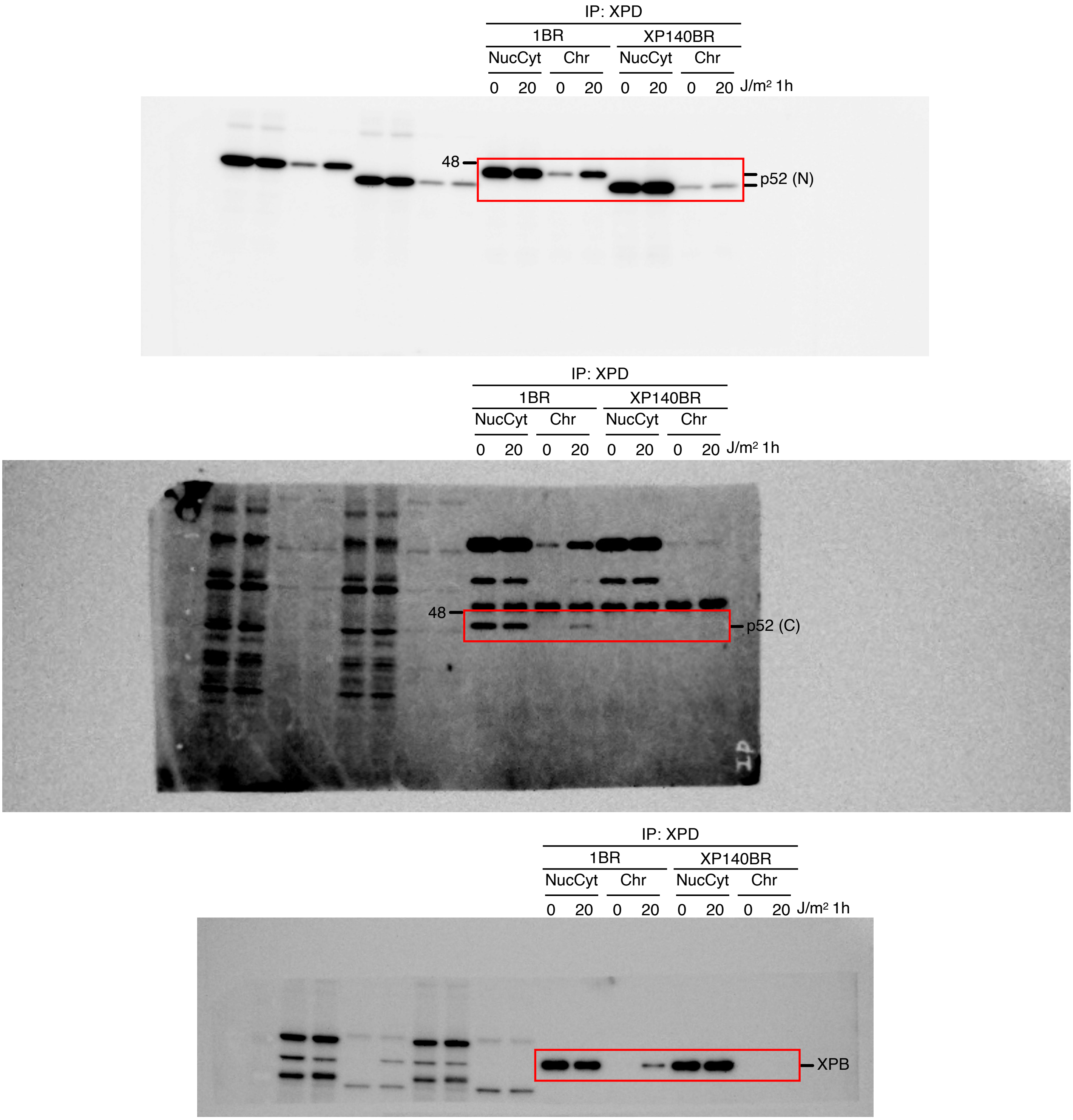
IP: XPD
1BR
XP140BR
NucCyt
Chr
NucCyt
Chr
0
20
0
20
0
20
0
20
J/m2 1h
48
p52 (N)
IP: XPD
1BR
XP140BR
NucCyt
Chr
NucCyt
Chr
0
20
0
20
0
20
0
20
J/m2 1h
48
p52 (C)
IP
IP: XPD
1BR
XP140BR
NucCyt
Chr
NucCyt
Chr
0
20
0
20
0
20
0
20
J/m2 1h
XPB

Fig2 c

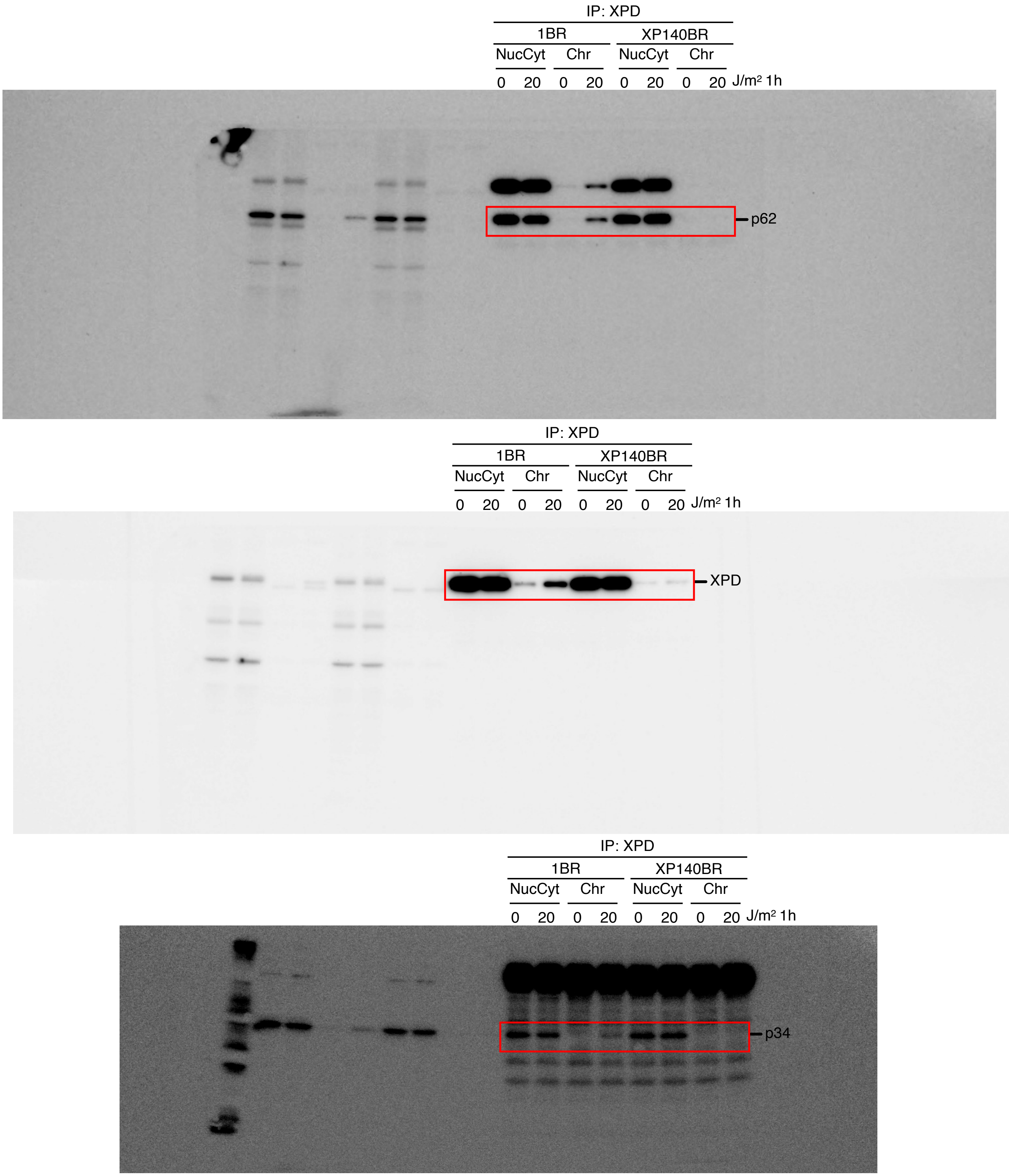
IP: XPD
1BR
XP140BR
NucCyt
Chr
NucCyt
Chr
0 20 0 20 0 20 0 20 J/m² 1h
p62
IP: XPD
1BR
XP140BR
NucCyt
Chr
NucCyt
Chr
0 20 0 20 0 20 0 20 J/m² 1h
XPD
IP: XPD
1BR
XP140BR
NucCyt
Chr
NucCyt
Chr
0 20 0 20 0 20 0 20 J/m² 1h
p34

Fig2 d

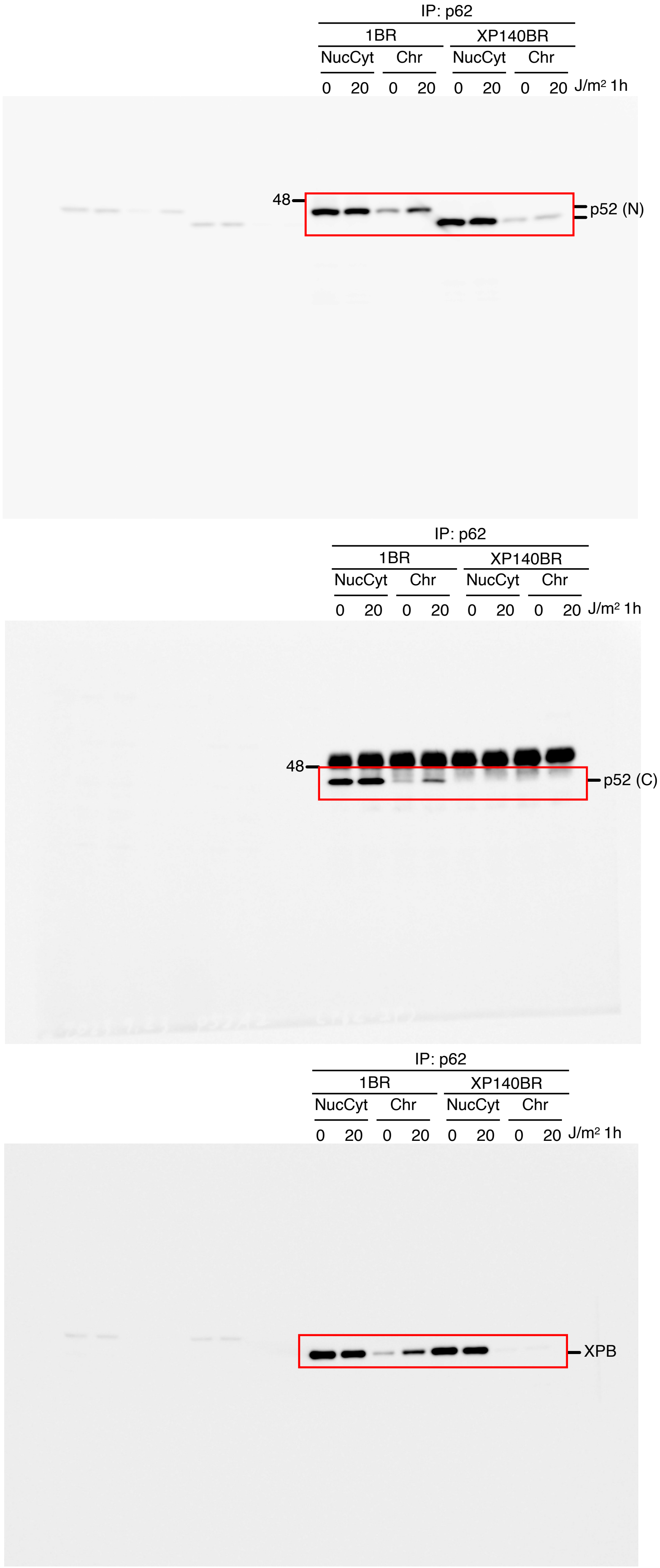
IP: p62
1BR
XP140BR
NucCyt
Chr
NucCyt
Chr
0 20 0 20 0 20 0 20 J/m2 1h
48
p52 (N)
IP: p62
1BR
XP140BR
NucCyt
Chr
NucCyt
Chr
0 20 0 20 0 20 0 20 J/m2 1h
48
p52 (C)
IP: p62
1BR
XP140BR
NucCyt
Chr
NucCyt
Chr
0 20 0 20 0 20 0 20 J/m2 1h
XPB

Fig2 d

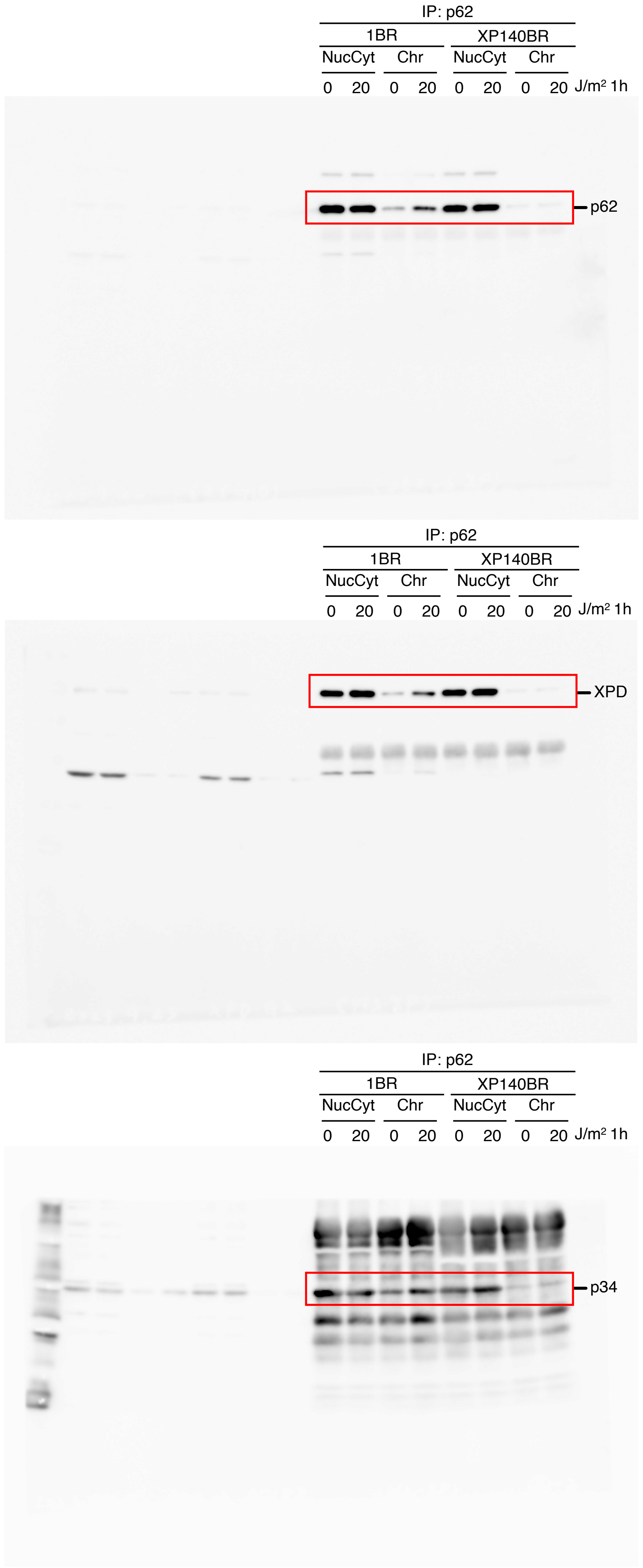

IP: p62
1BR
XP140BR
NucCyt
Chr
NucCyt
Chr
0 20 0 20 0 20 0 20 J/m² 1h
p62
IP: p62
1BR
XP140BR
NucCyt
Chr
NucCyt
Chr
0 20 0 20 0 20 0 20 J/m² 1h
XPD
IP: p62
1BR
XP140BR
NucCyt
Chr
NucCyt
Chr
0 20 0 20 0 20 0 20 J/m² 1h
p34

Fig2 e

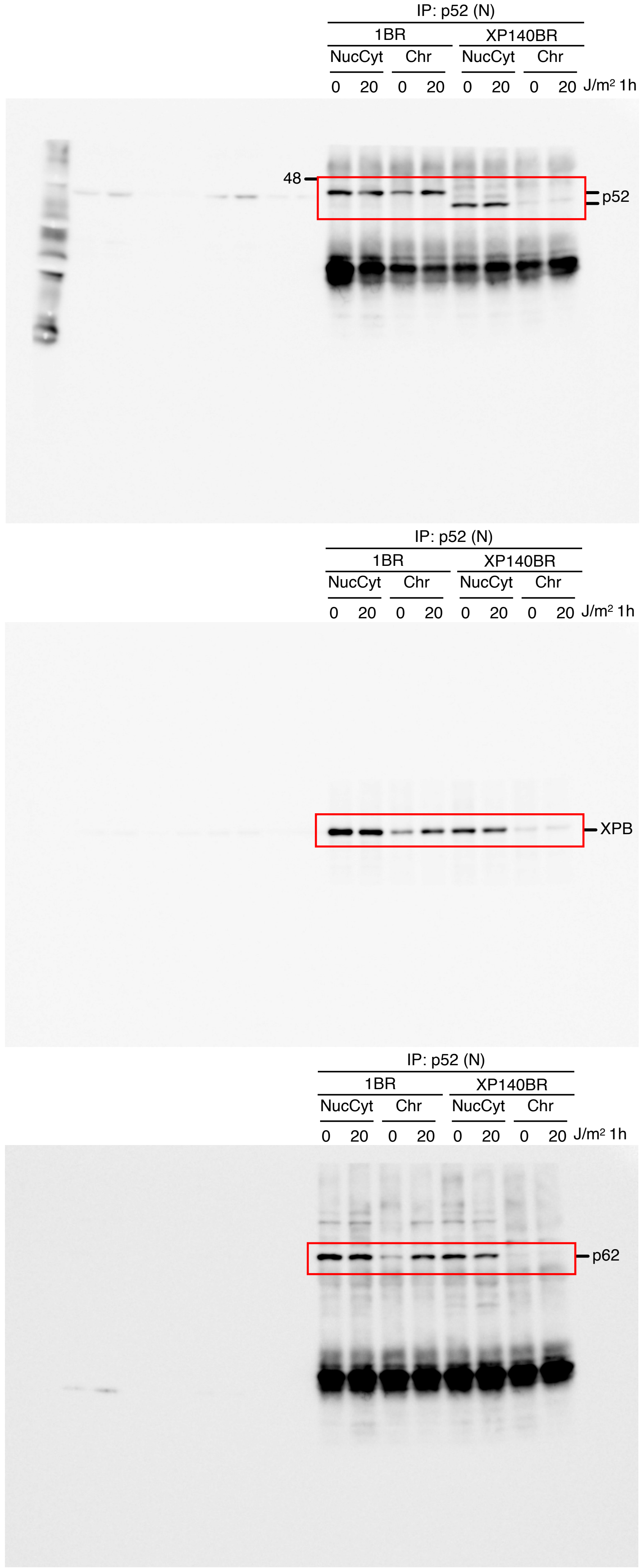

IP: p52 (N)
1BR
XP140BR
NucCyt
Chr
NucCyt
Chr
0
20
0
20
0
20
0
20
J/m2 1h
48
p52
IP: p52 (N)
1BR
XP140BR
NucCyt
Chr
NucCyt
Chr
0
20
0
20
0
20
0
20
J/m2 1h
XPB
IP: p52 (N)
1BR
XP140BR
NucCyt
Chr
NucCyt
Chr
0
20
0
20
0
20
0
20
J/m2 1h
p62

Fig2 e

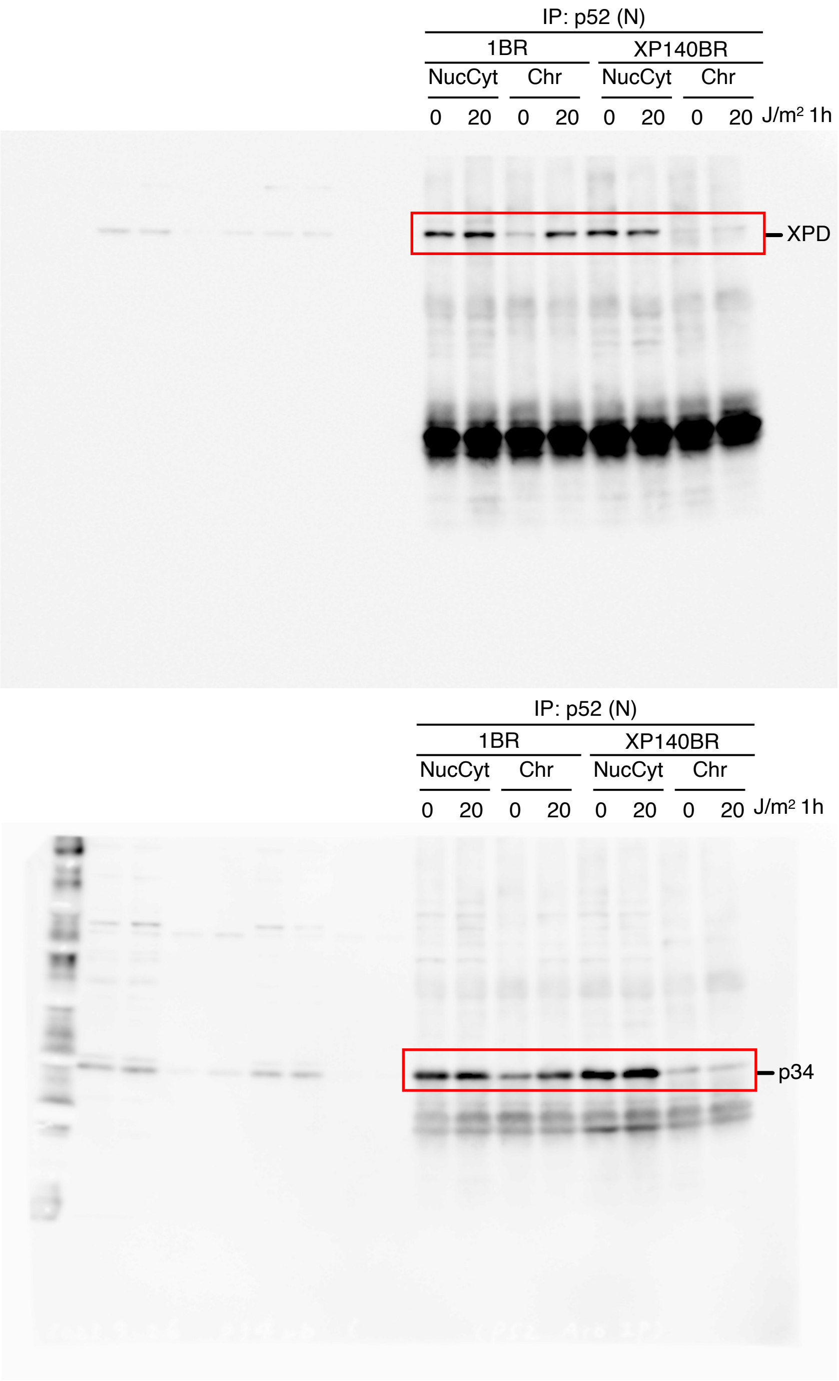
IP: p52 (N)
1BR
XP140BR
NucCyt
Chr
NucCyt
Chr
0
20
0
20
0
20
0
20
J/m² 1h
XPD
IP: p52 (N)
1BR
XP140BR
NucCyt
Chr
NucCyt
Chr
0
20
0
20
0
20
0
20
J/m² 1h
p34

Fig3 a

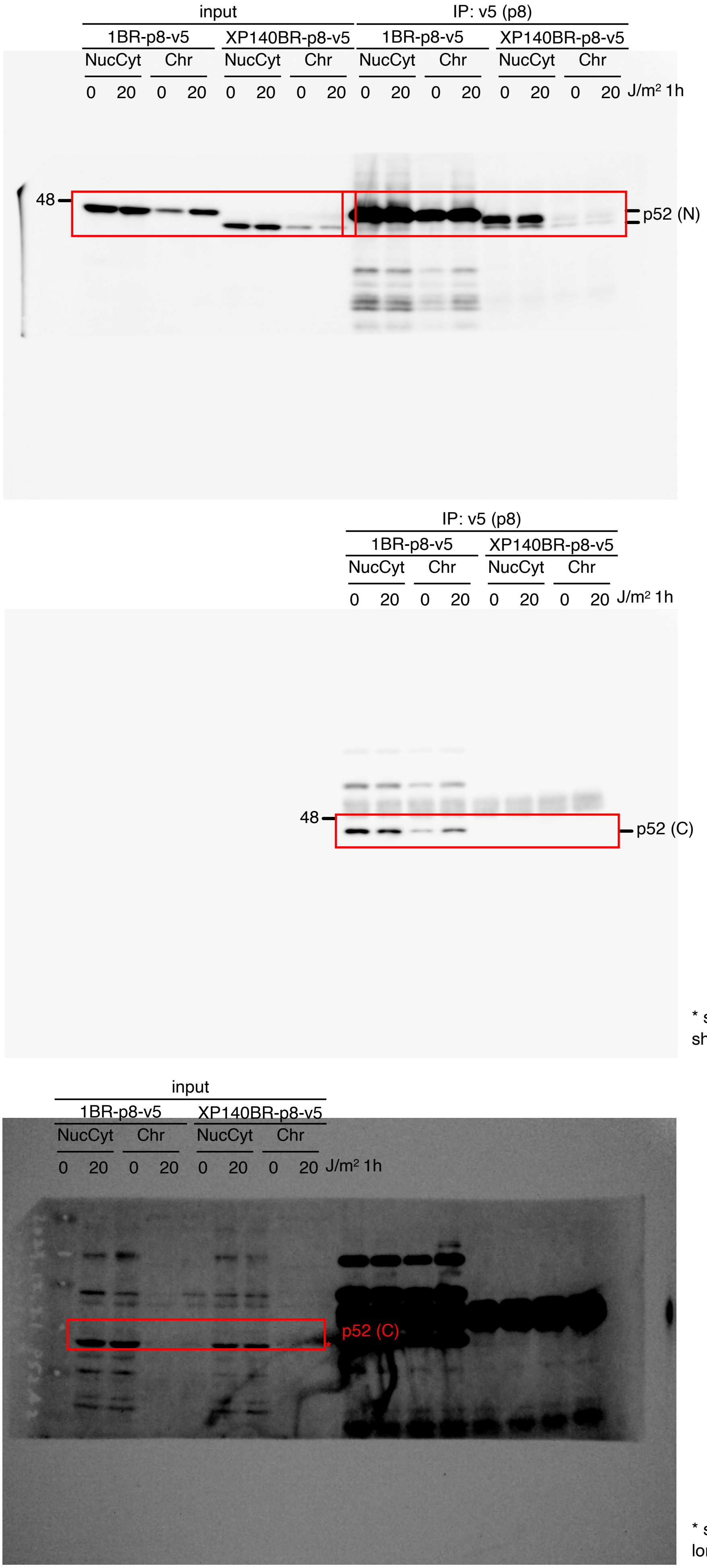

* same membrane
short exp.

* same membrane
long exp.

Fig3 a

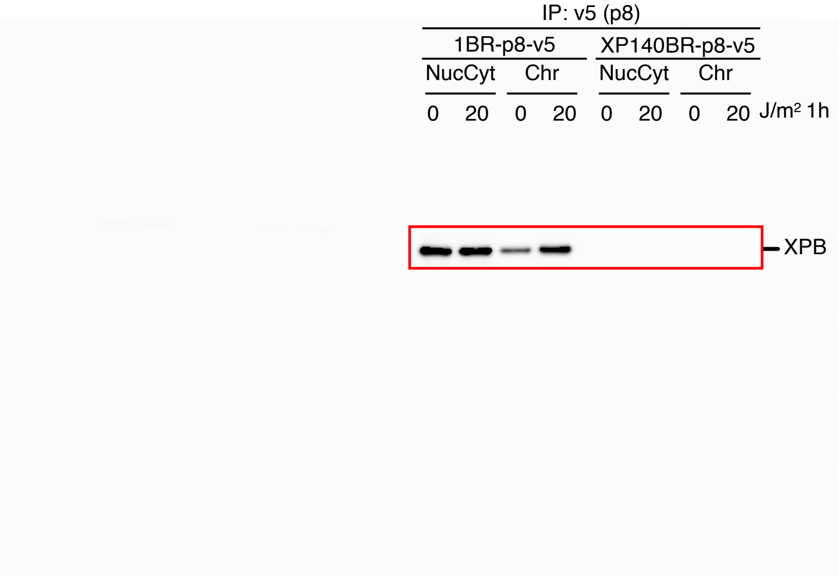
IP: v5 (p8)
1BR-p8-v5
XP140BR-p8-v5
NucCyt
Chr
NucCyt
Chr
0 20 0 20 0 20 0 20 J/m² 1h
XPB

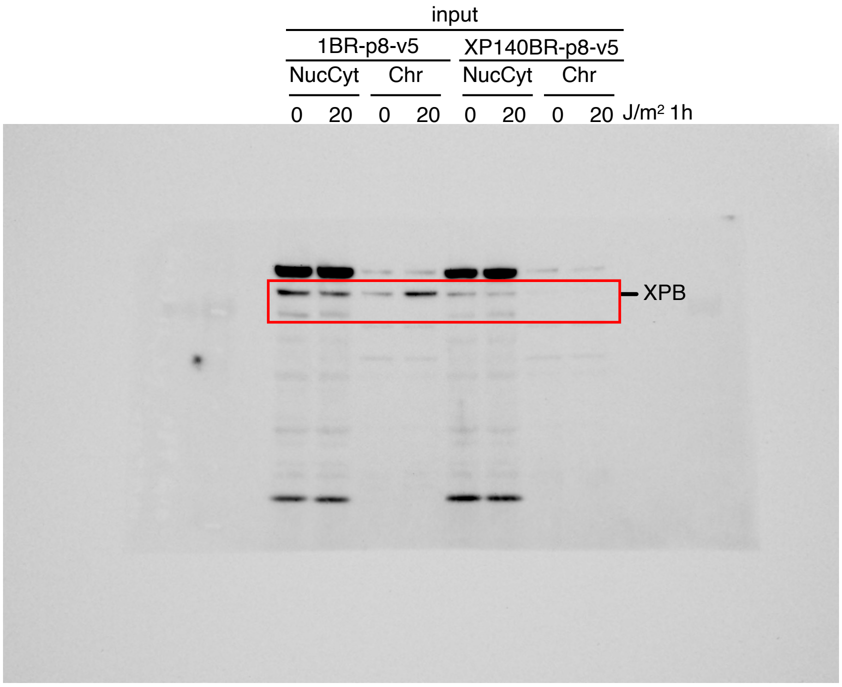
input
1BR-p8-v5
XP140BR-p8-v5
NucCyt
Chr
NucCyt
Chr
0 20 0 20 0 20 0 20 J/m² 1h
XPB

Fig3 a

IP: v5 (p8)

1BR-p8-v5				XP140BR-p8-v5			
NucCyt		Chr		NucCyt		Chr	
0	20	0	20	0	20	0	20

J/m² 1h

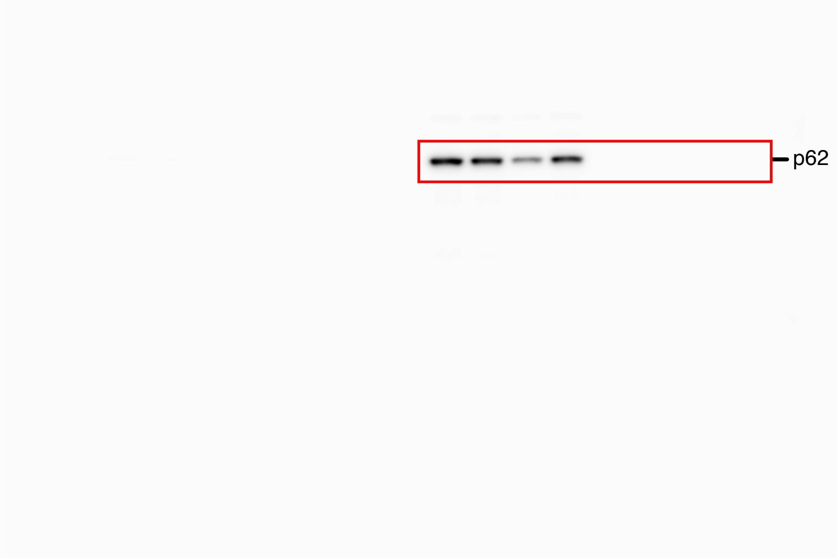

** same membrane
short exp.

input

1BR-p8-v5				XP140BR-p8-v5			
NucCyt		Chr		NucCyt		Chr	
0	20	0	20	0	20	0	20

J/m² 1h

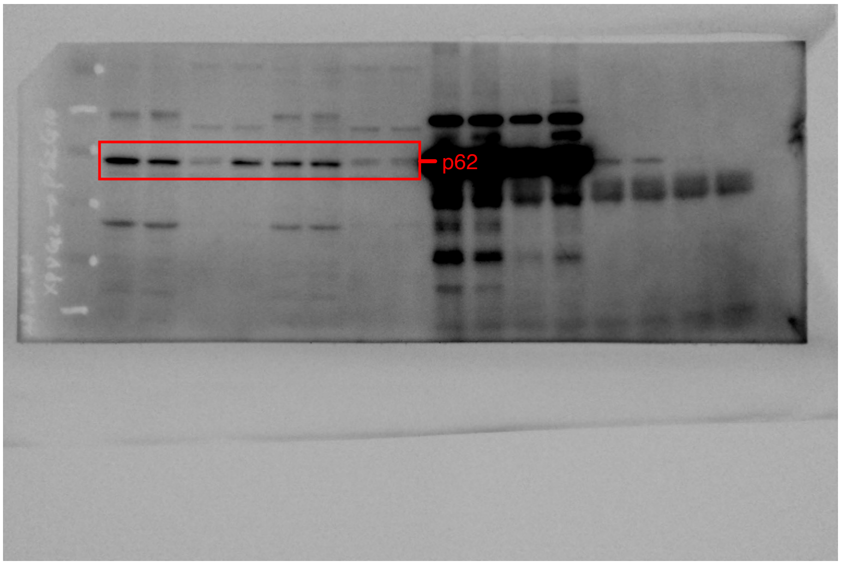

** same membrane
long exp.

Fig3 a

IP: v5 (p8)

1BR-p8-v5 | XP140BR-p8-v5

NucCyt | Chr | NucCyt | Chr

0 20 0 20 0 20 0 20 J/m2 1h

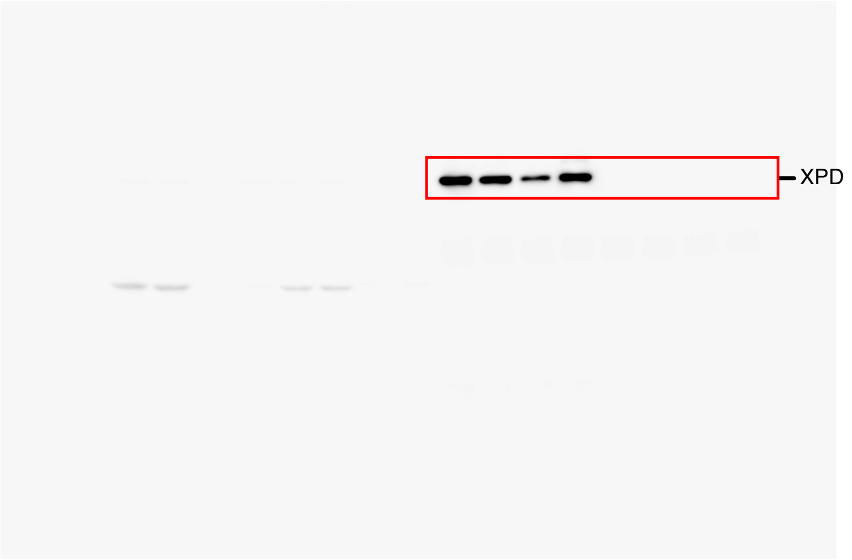

*** same membrane
short exp.

input

1BR-p8-v5 | XP140BR-p8-v5

NucCyt | Chr | NucCyt | Chr

0 20 0 20 0 20 0 20 J/m2 1h

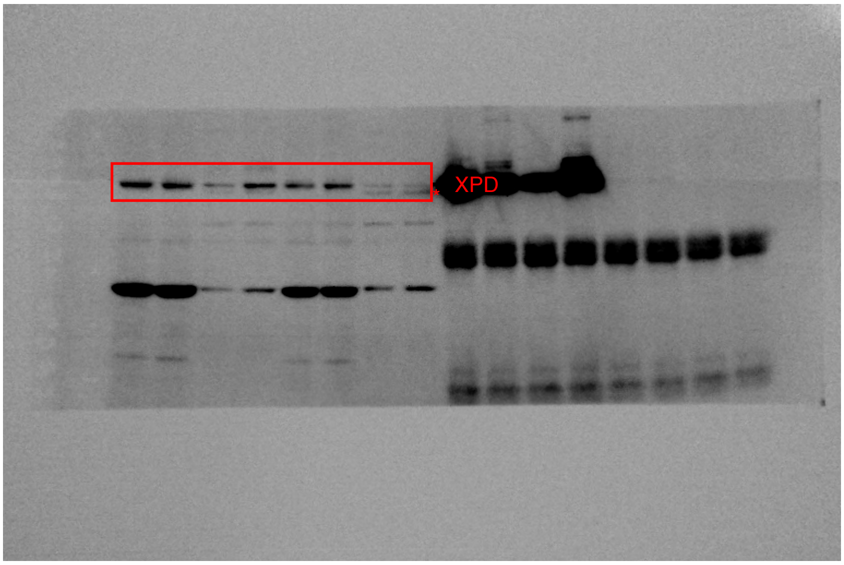

*** same membrane
long exp.

Fig3 a

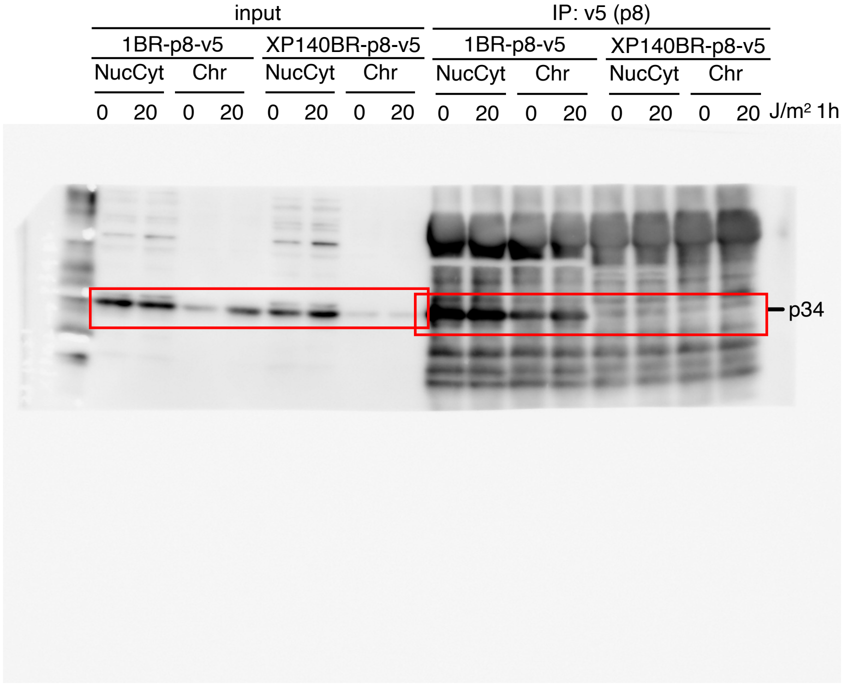
input
IP: v5 (p8)
1BR-p8-v5
XP140BR-p8-v5
1BR-p8-v5
XP140BR-p8-v5
NucCyt
Chr
NucCyt
Chr
NucCyt
Chr
NucCyt
Chr
0 20 0 20 0 20 0 20 0 20 0 20 0 20 0 20 J/m² 1h
p34

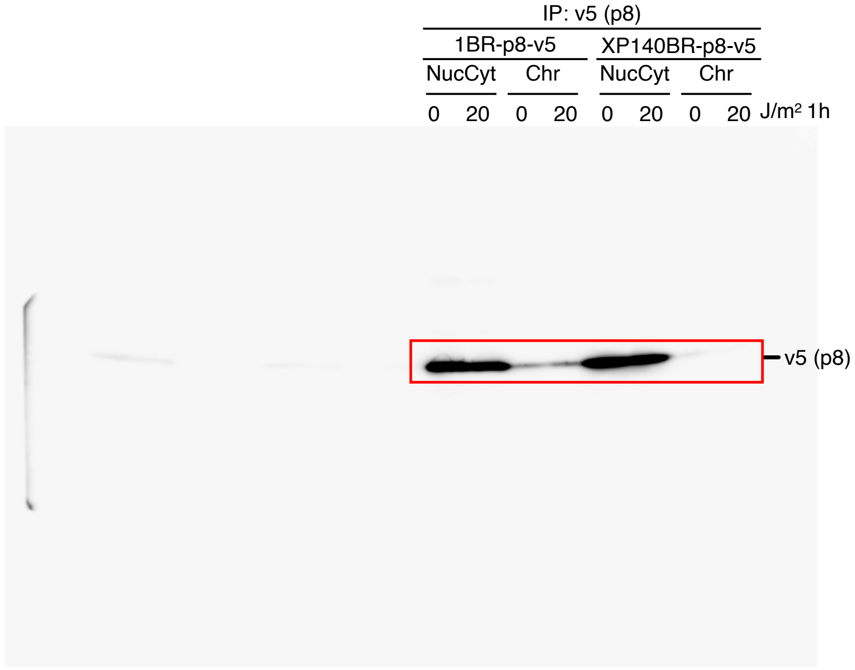
IP: v5 (p8)
1BR-p8-v5
XP140BR-p8-v5
NucCyt
Chr
NucCyt
Chr
0 20 0 20 0 20 0 20 J/m² 1h
v5 (p8)

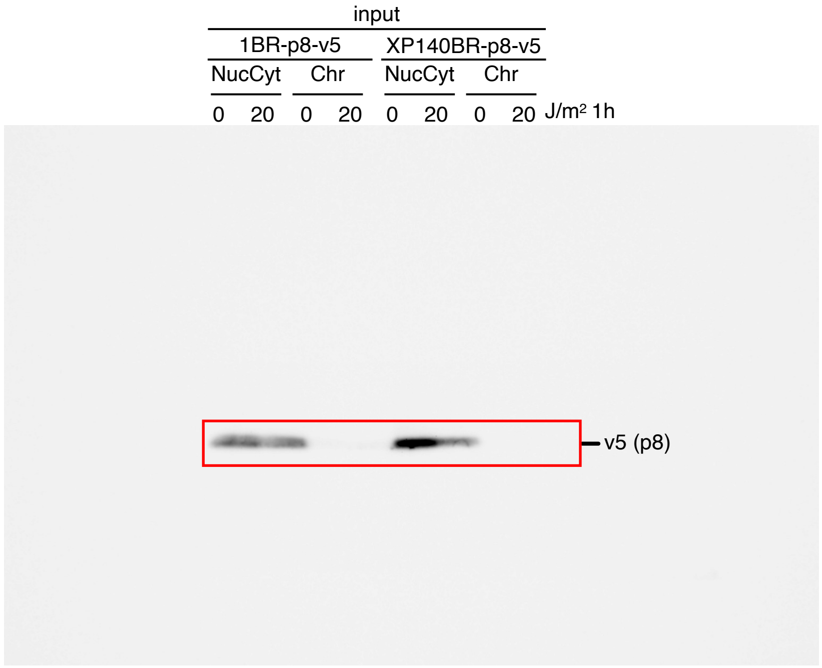
input
1BR-p8-v5
XP140BR-p8-v5
NucCyt
Chr
NucCyt
Chr
0 20 0 20 0 20 0 20 J/m² 1h
v5 (p8)

Fig3 a

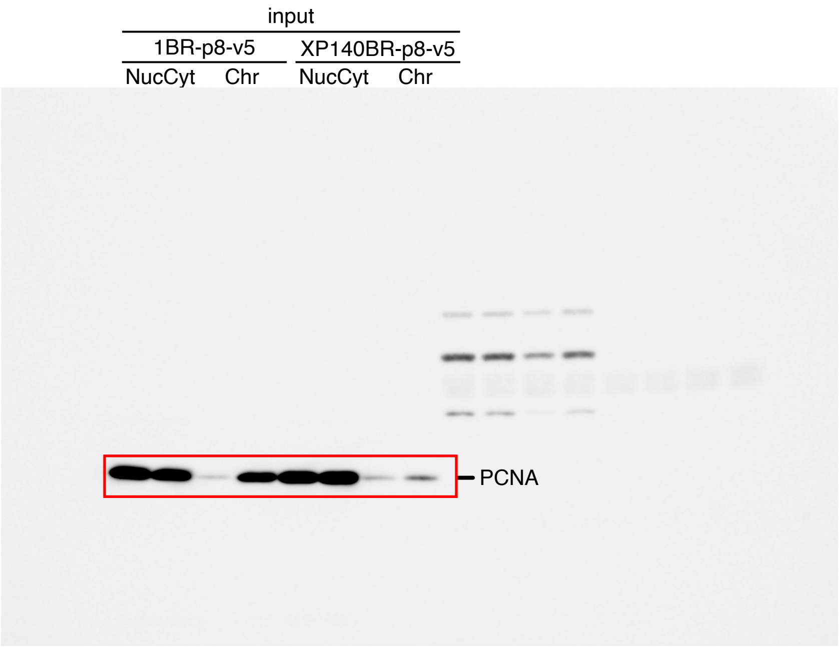

Fig3 b

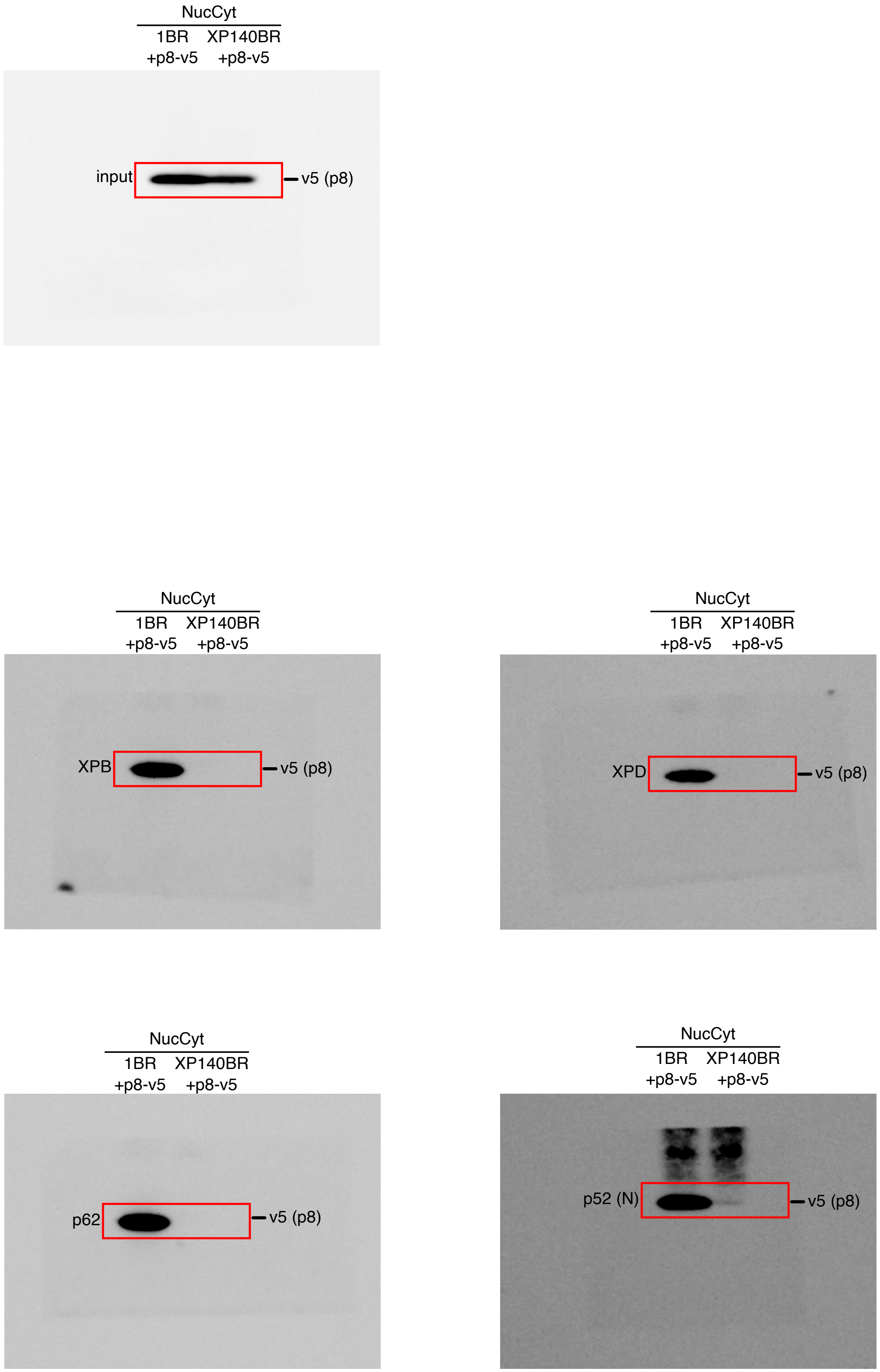
NucCyt
1BR
+p8-v5
XP140BR
+p8-v5
input
v5 (p8)
NucCyt
1BR
+p8-v5
XP140BR
+p8-v5
XPB
v5 (p8)
NucCyt
1BR
+p8-v5
XP140BR
+p8-v5
XPD
v5 (p8)
NucCyt
1BR
+p8-v5
XP140BR
+p8-v5
p62
v5 (p8)
NucCyt
1BR
+p8-v5
XP140BR
+p8-v5
p52 (N)
v5 (p8)

Fig3 d

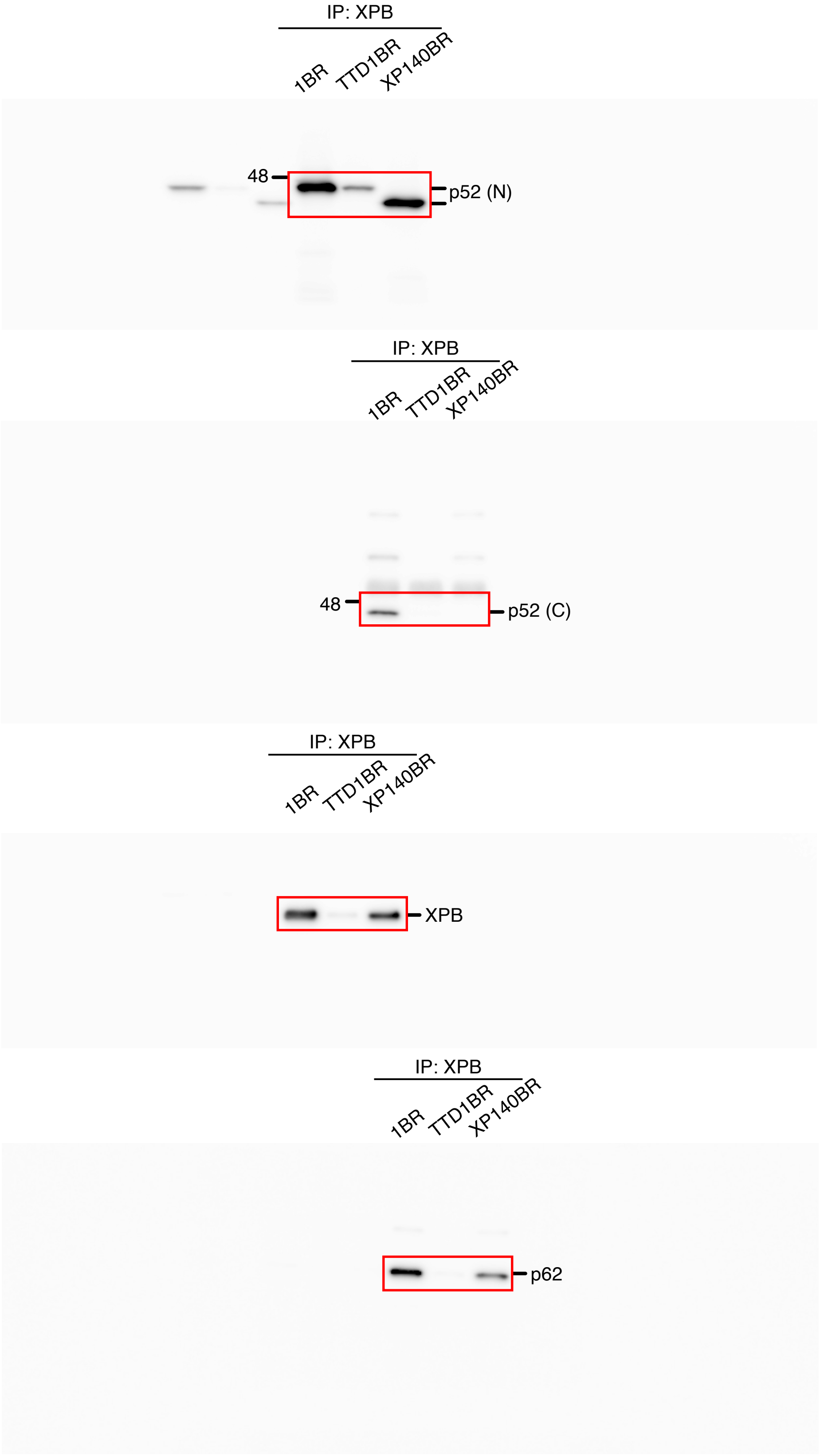
IP: XPB
1BR
TTD1BR
XP140BR
48
p52 (N)
IP: XPB
1BR
TTD1BR
XP140BR
48
p52 (C)
IP: XPB
1BR
TTD1BR
XP140BR
XPB
IP: XPB
1BR
TTD1BR
XP140BR
p62

Fig3 d

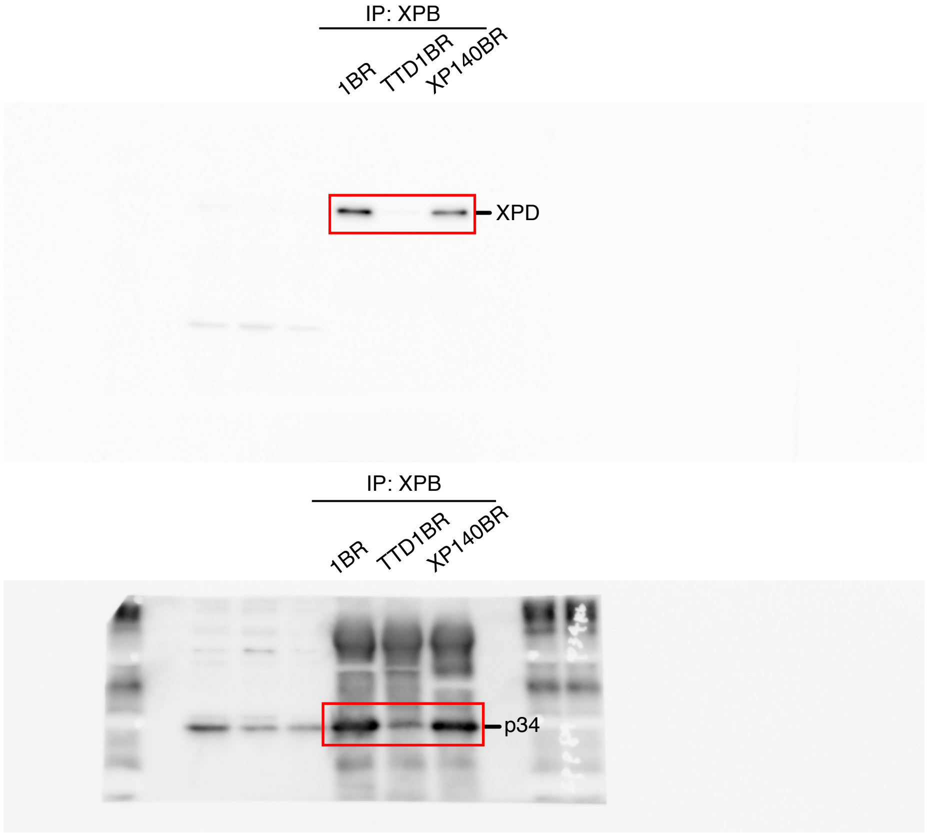

Fig3 d

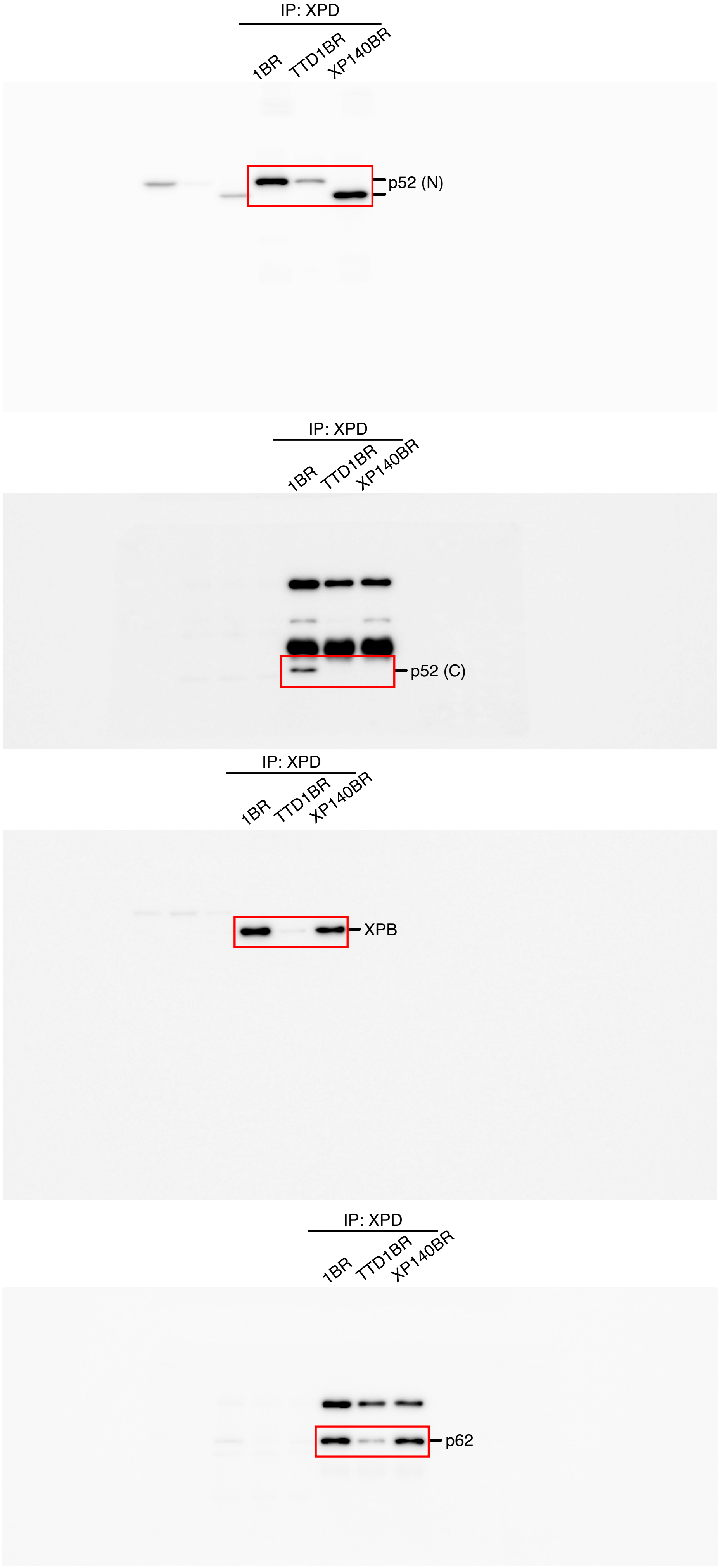
IP: XPD
1BR
TTD1BR
XP140BR
p52 (N)
IP: XPD
1BR
TTD1BR
XP140BR
p52 (C)
IP: XPD
1BR
TTD1BR
XP140BR
XPB
IP: XPD
1BR
TTD1BR
XP140BR
p62

Fig3 d

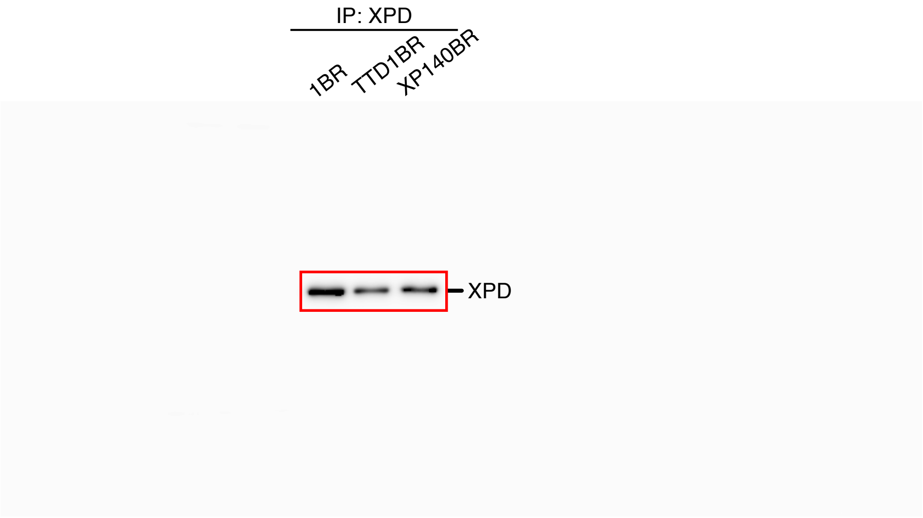

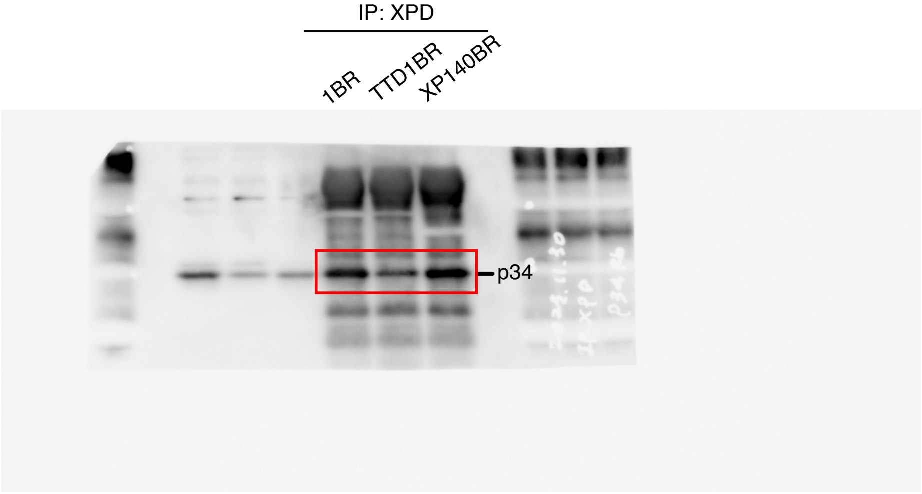

Fig3 d

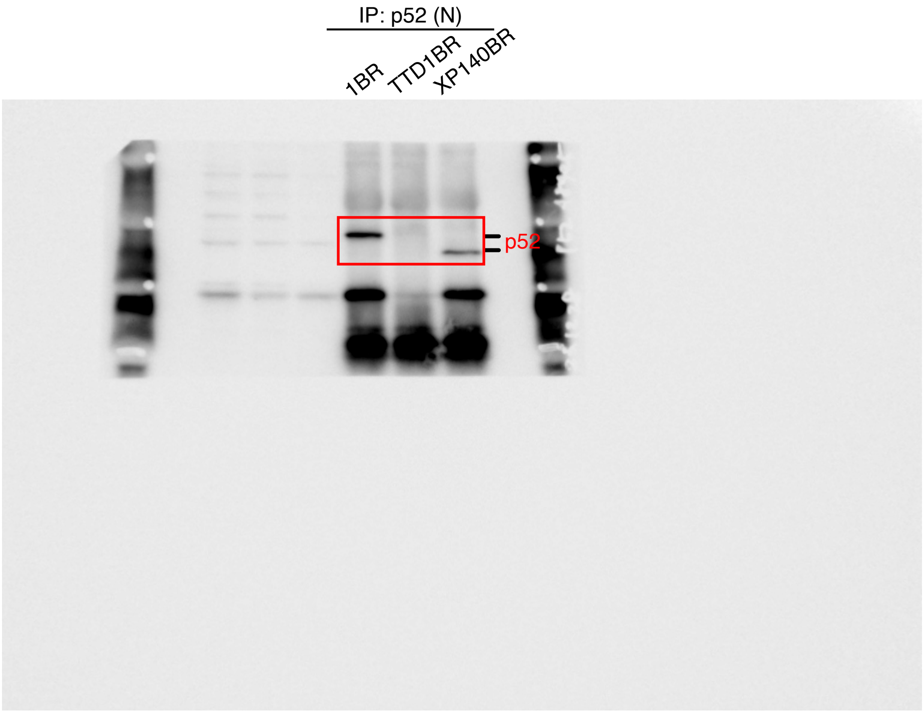
IP: p52 (N)
1BR
TTD1BR
XP140BR
p52

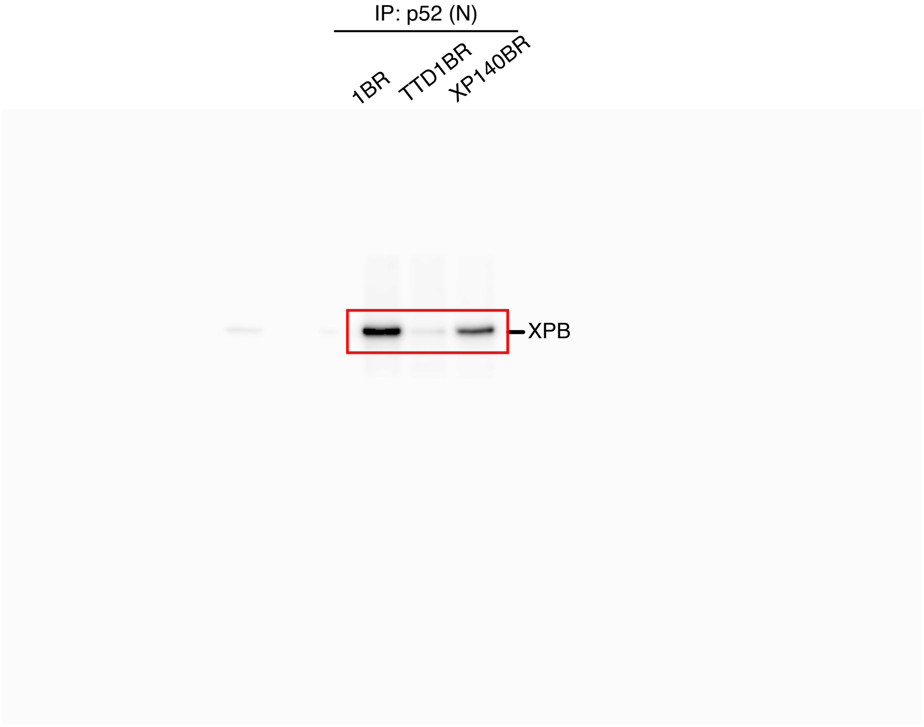
IP: p52 (N)
1BR
TTD1BR
XP140BR
XPB

Fig3 d

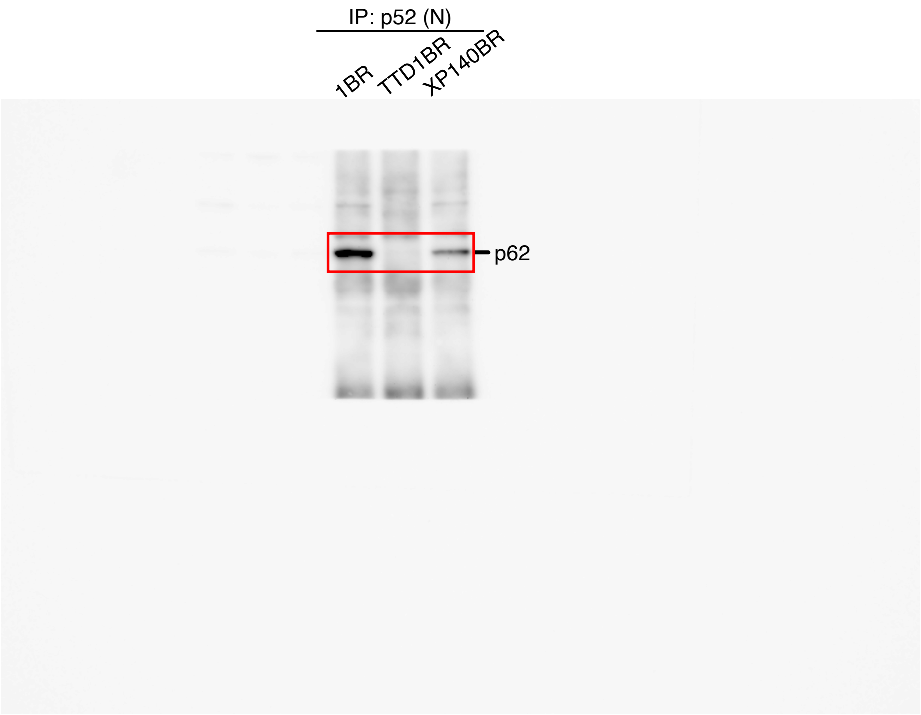
IP: p52 (N)
1BR
TTD1BR
XP140BR
p62

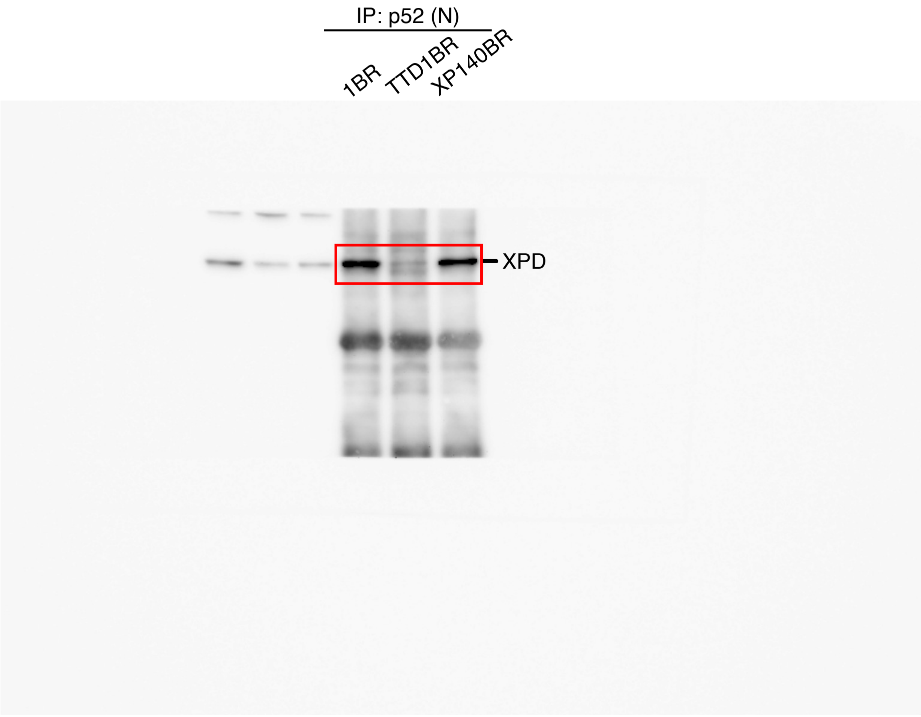
IP: p52 (N)
1BR
TTD1BR
XP140BR
XPD

Fig3 d

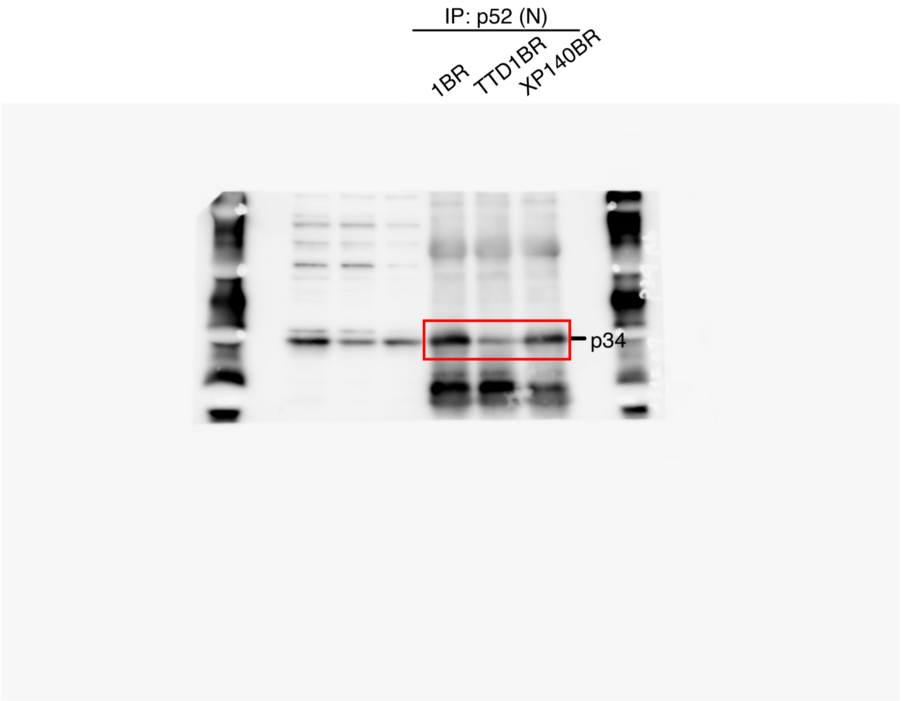

Fig3 e

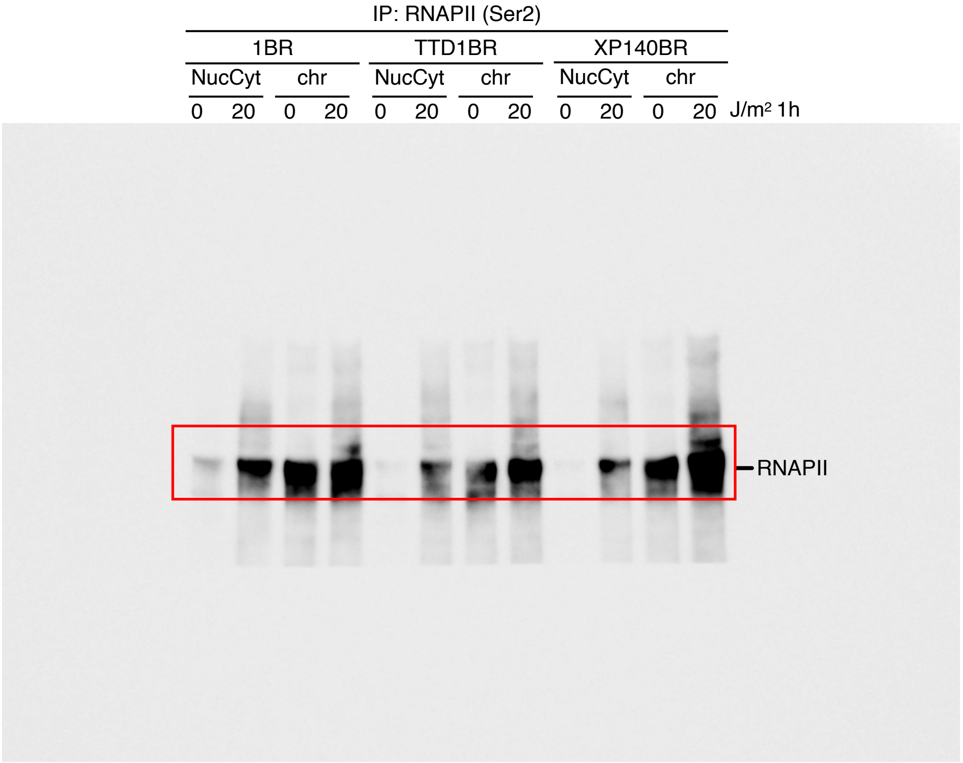
IP: RNAPII (Ser2)
1BR
TTD1BR
XP140BR
NucCyt
chr
NucCyt
chr
NucCyt
chr
0 20 0 20 0 20 0 20 0 20 0 20 J/m² 1h
RNAPII

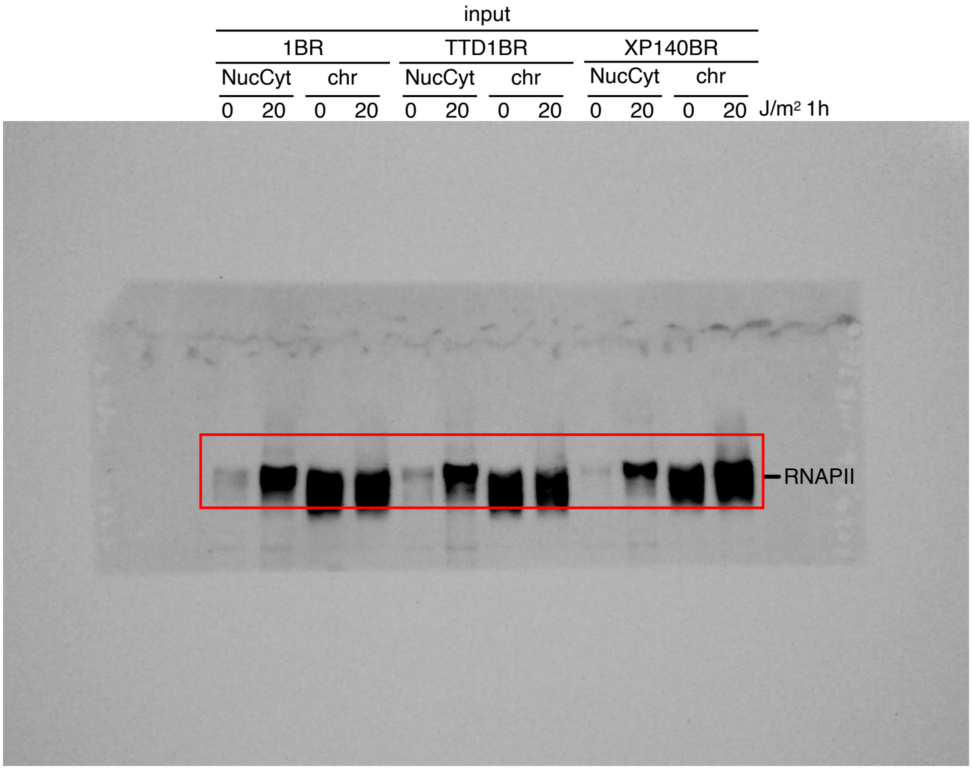
input
1BR
TTD1BR
XP140BR
NucCyt
chr
NucCyt
chr
NucCyt
chr
0 20 0 20 0 20 0 20 0 20 0 20 J/m² 1h
RNAPII

Fig3 e

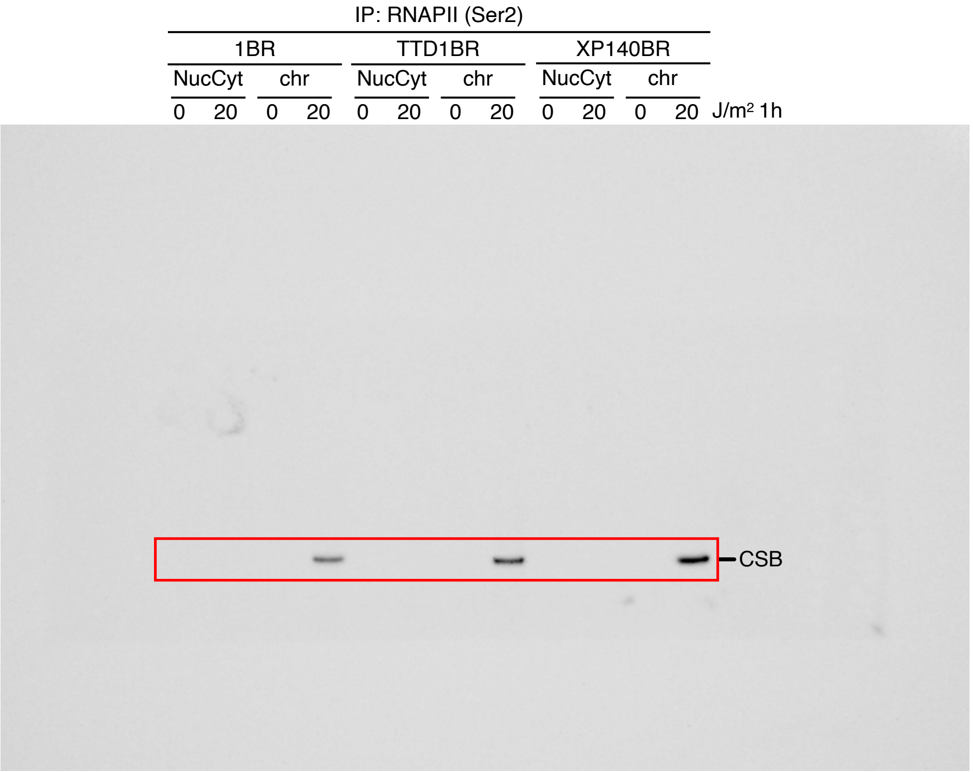

IP: RNAPII (Ser2)
1BR
TTD1BR
XP140BR
NucCyt
chr
NucCyt
chr
NucCyt
chr
0 20 0 20 0 20 0 20 0 20 0 20 J/m² 1h
CSB

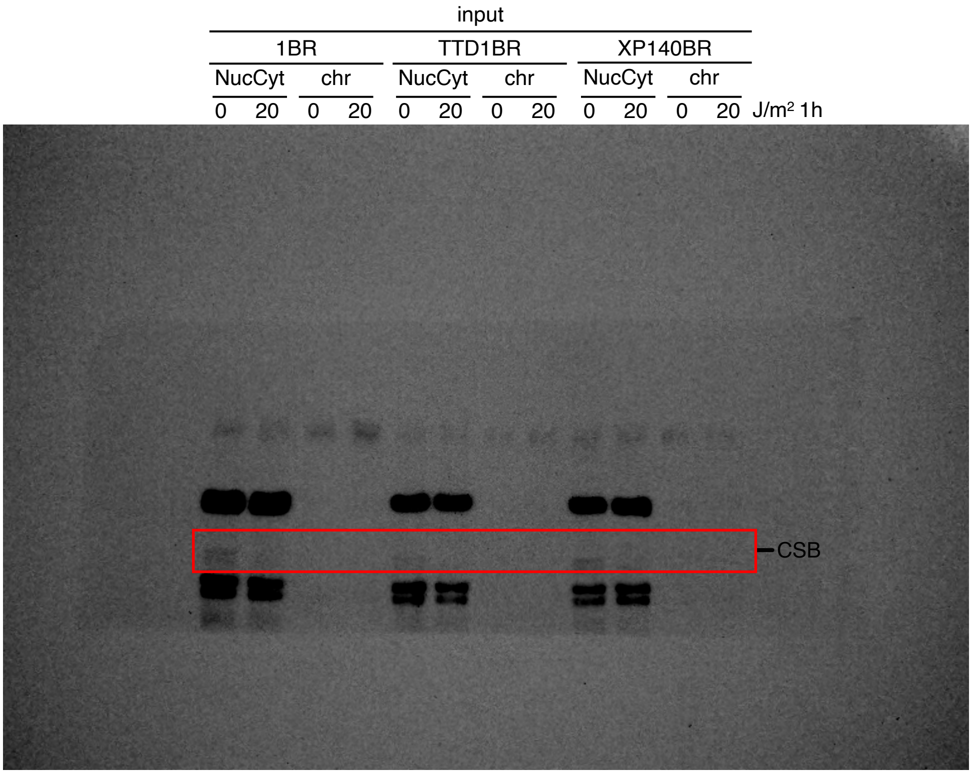

input
1BR
TTD1BR
XP140BR
NucCyt
chr
NucCyt
chr
NucCyt
chr
0 20 0 20 0 20 0 20 0 20 0 20 J/m² 1h
CSB

Fig3 e

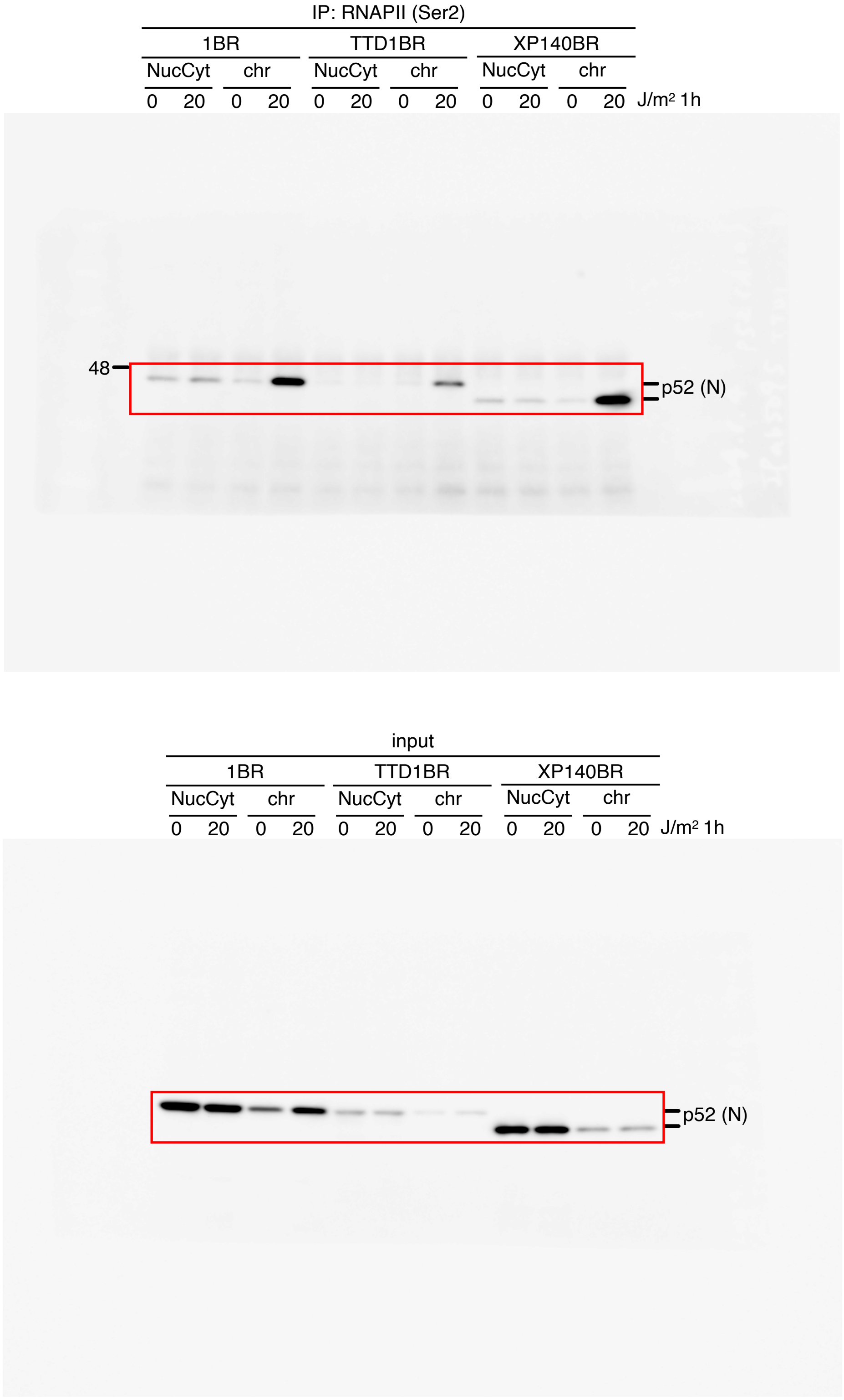
IP: RNAPII (Ser2)
1BR
TTD1BR
XP140BR
NucCyt
chr
0
20
J/m² 1h
48
p52 (N)
input

Fig3 e

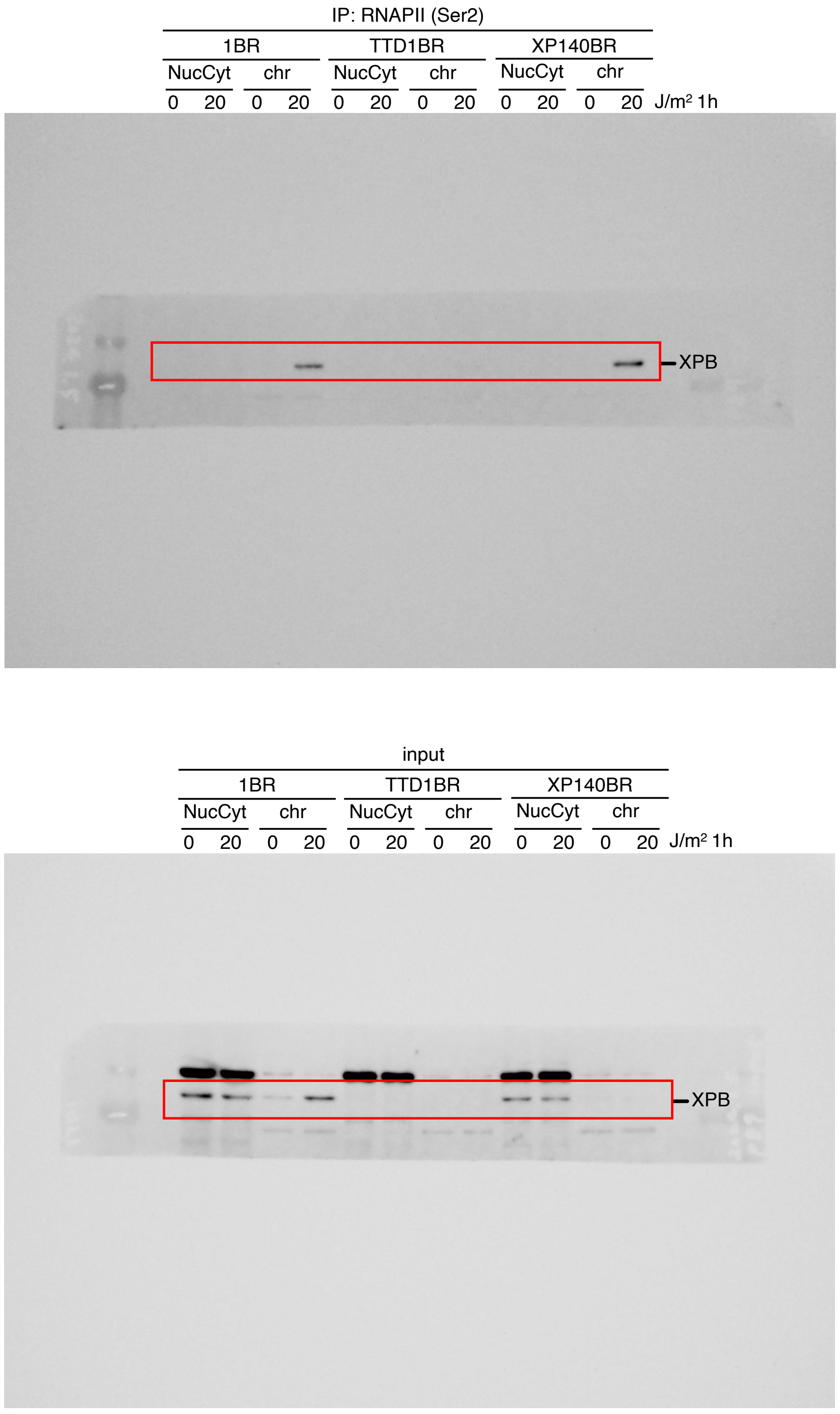
IP: RNAPII (Ser2)
1BR
TTD1BR
XP140BR
NucCyt
chr
NucCyt
chr
NucCyt
chr
0 20 0 20 0 20 0 20 0 20 0 20 J/m² 1h
XPB
input
1BR
TTD1BR
XP140BR
NucCyt
chr
NucCyt
chr
NucCyt
chr
0 20 0 20 0 20 0 20 0 20 0 20 J/m² 1h
XPB

Fig3 e

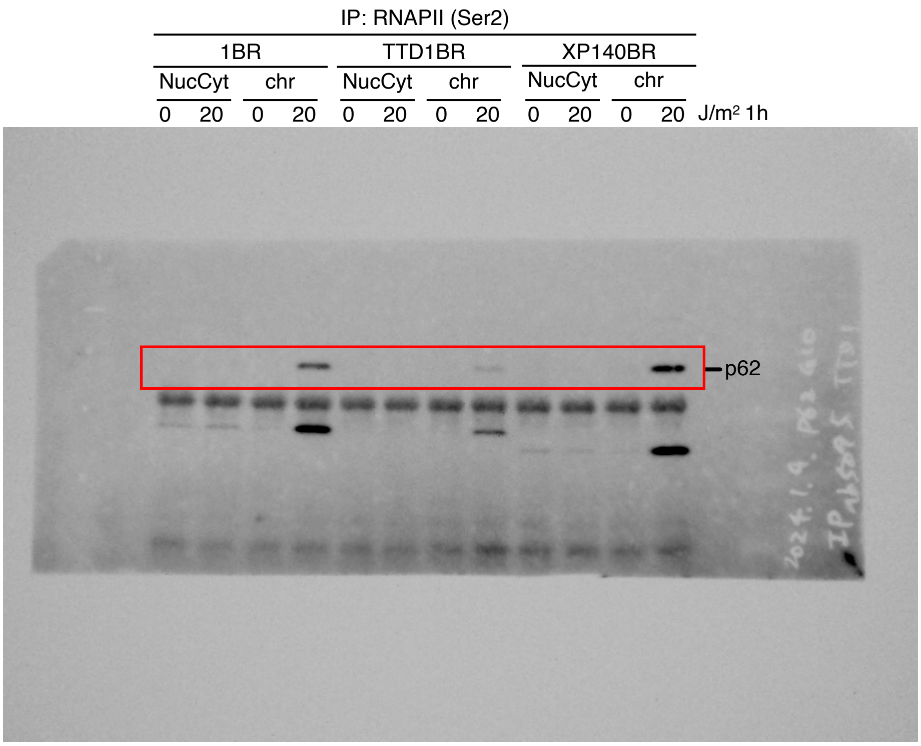
IP: RNAPII (Ser2)
1BR
TTD1BR
XP140BR
NucCyt
chr
NucCyt
chr
NucCyt
chr
0 20 0 20 0 20 0 20 0 20 0 20 J/m² 1h
p62

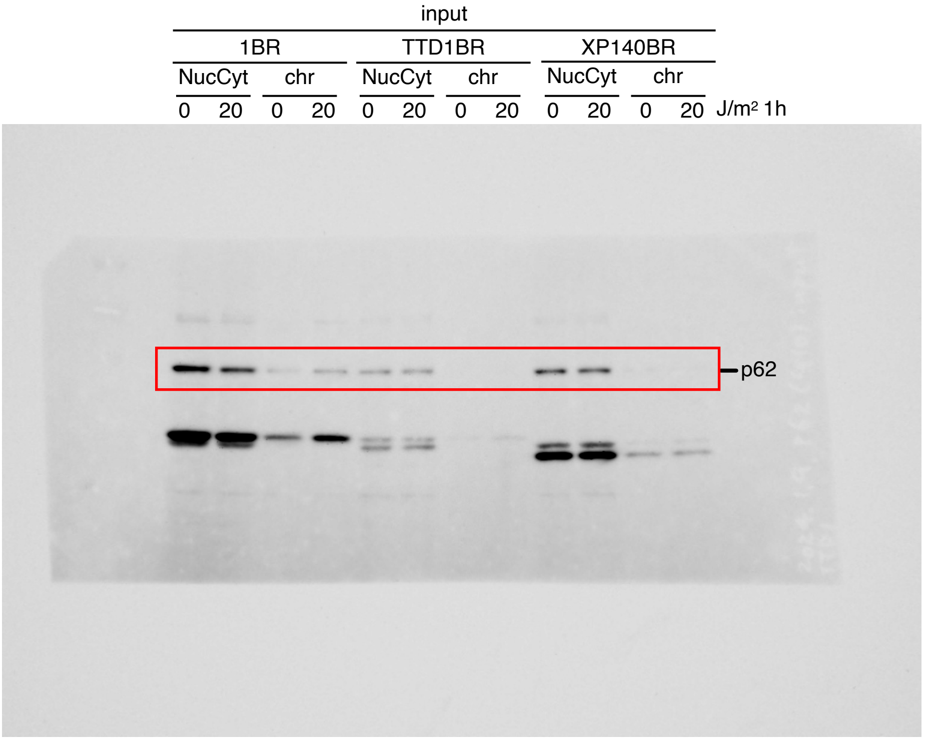
input
1BR
TTD1BR
XP140BR
NucCyt
chr
NucCyt
chr
NucCyt
chr
0 20 0 20 0 20 0 20 0 20 0 20 J/m² 1h
p62

Fig3 e

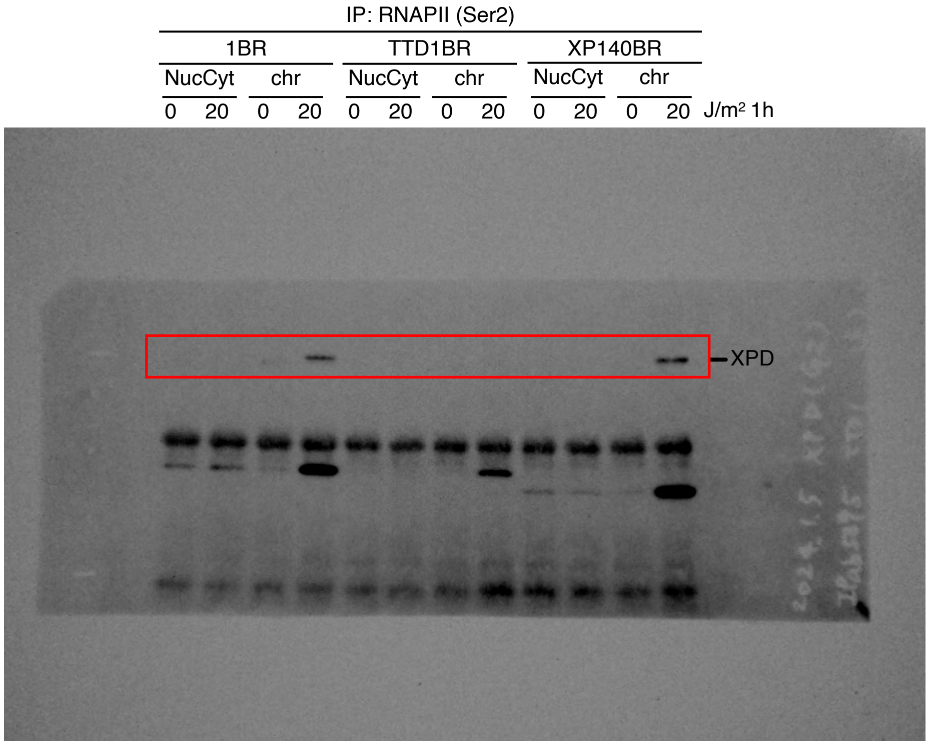
IP: RNAPII (Ser2)
1BR
TTD1BR
XP140BR
NucCyt chr NucCyt chr NucCyt chr
0 20 0 20 0 20 0 20 0 20 0 20 J/m² 1h
XPD

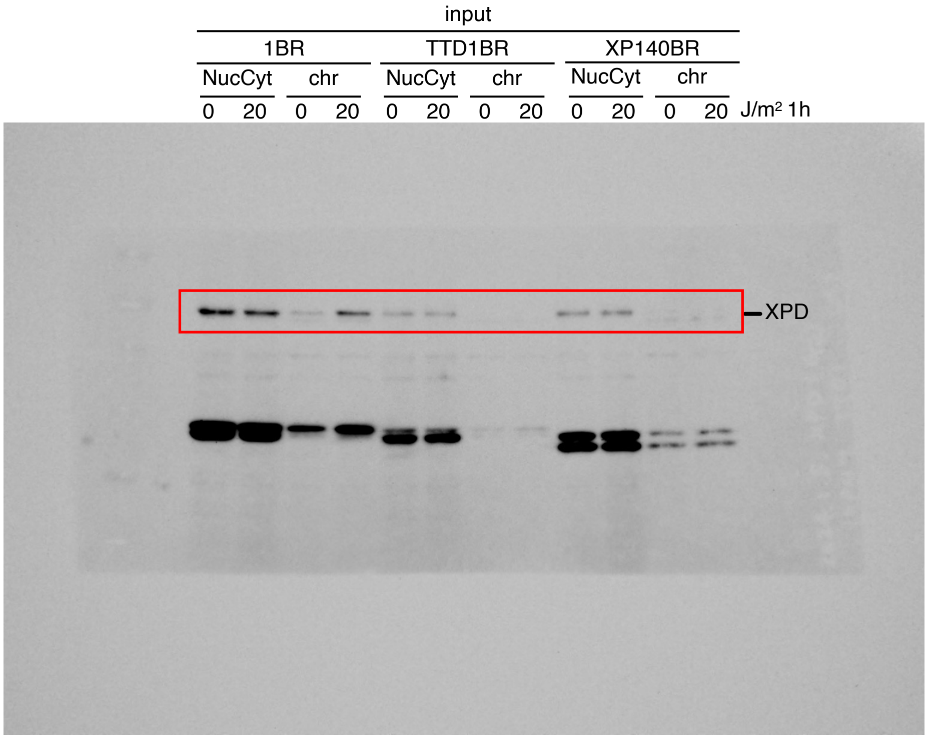
input
1BR
TTD1BR
XP140BR
NucCyt chr NucCyt chr NucCyt chr
0 20 0 20 0 20 0 20 0 20 0 20 J/m² 1h
XPD

Fig3 e

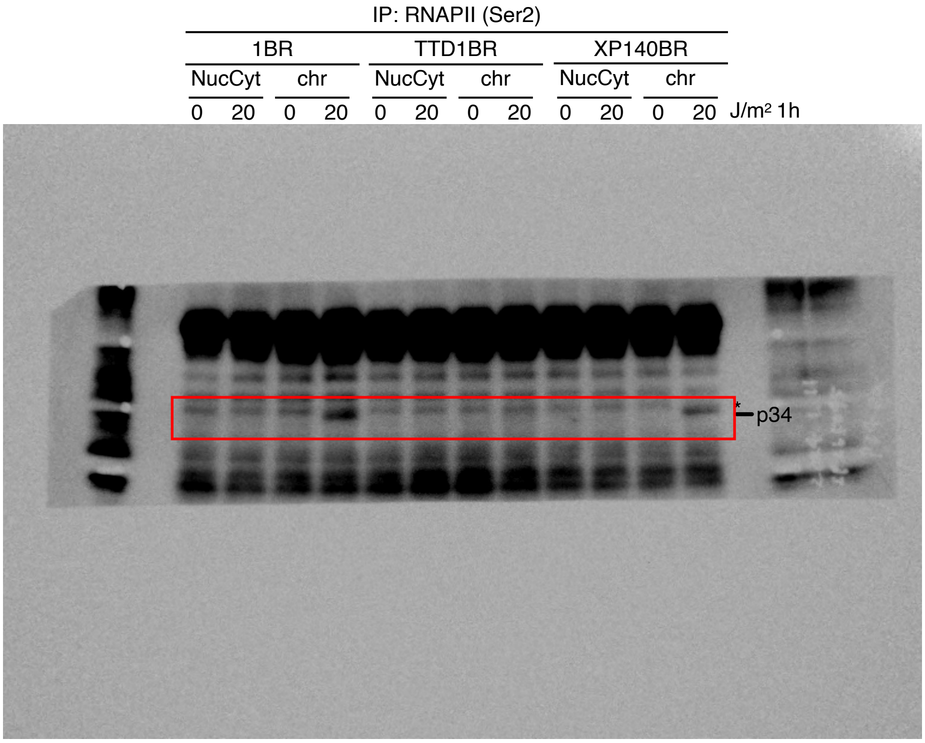
IP: RNAPII (Ser2)
1BR
TTD1BR
XP140BR
NucCyt
chr
NucCyt
chr
NucCyt
chr
0 20 0 20 0 20 0 20 0 20 0 20 J/m2 1h
*
p34

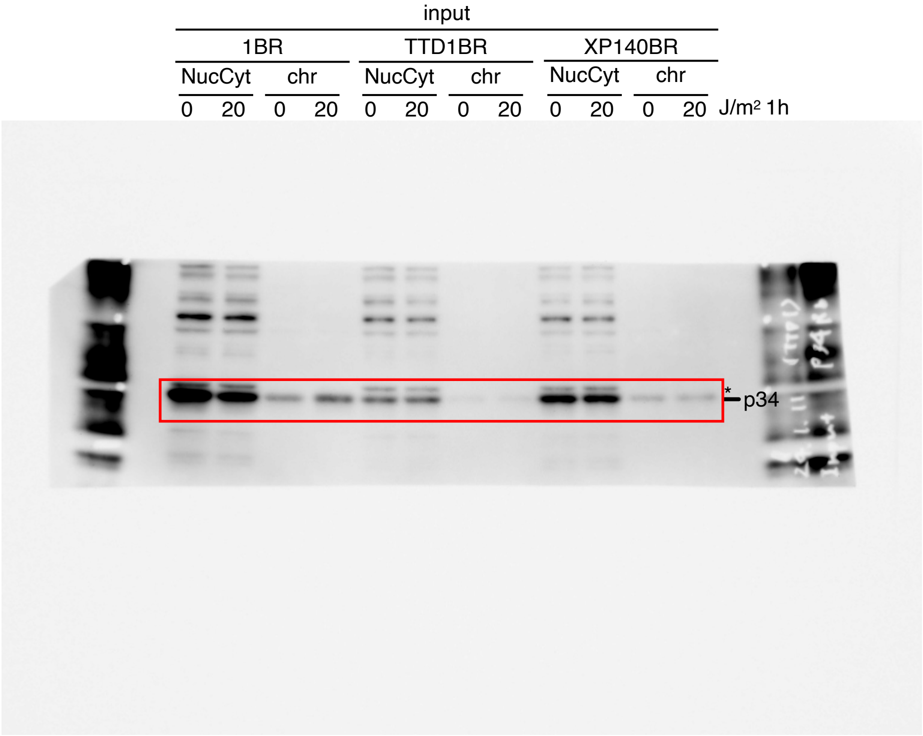
input
1BR
TTD1BR
XP140BR
NucCyt
chr
NucCyt
chr
NucCyt
chr
0 20 0 20 0 20 0 20 0 20 0 20 J/m2 1h
*
p34

Fig3 e

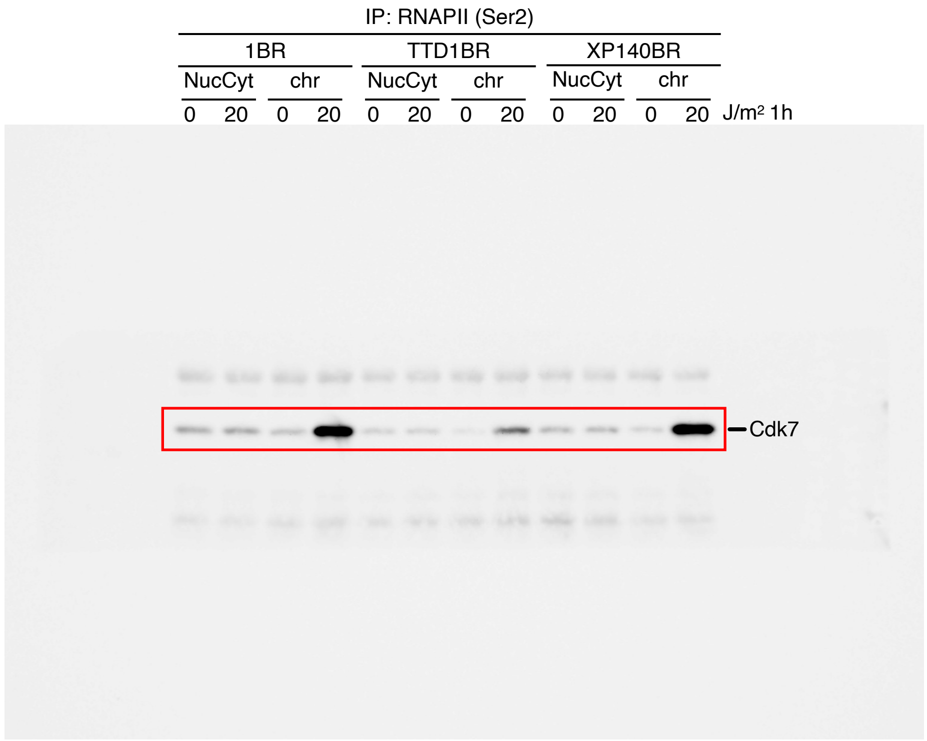
IP: RNAPII (Ser2)
1BR
TTD1BR
XP140BR
NucCyt
chr
NucCyt
chr
NucCyt
chr
0 20 0 20 0 20 0 20 0 20 0 20 J/m² 1h
Cdk7

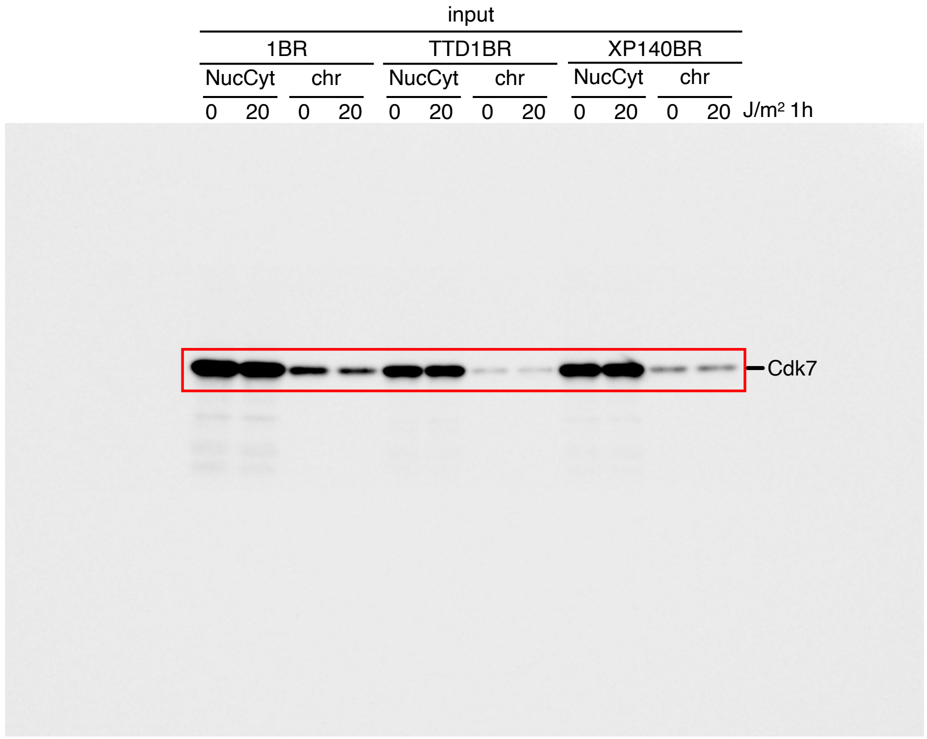
input
1BR
TTD1BR
XP140BR
NucCyt
chr
NucCyt
chr
NucCyt
chr
0 20 0 20 0 20 0 20 0 20 0 20 J/m² 1h
Cdk7

Fig3 e

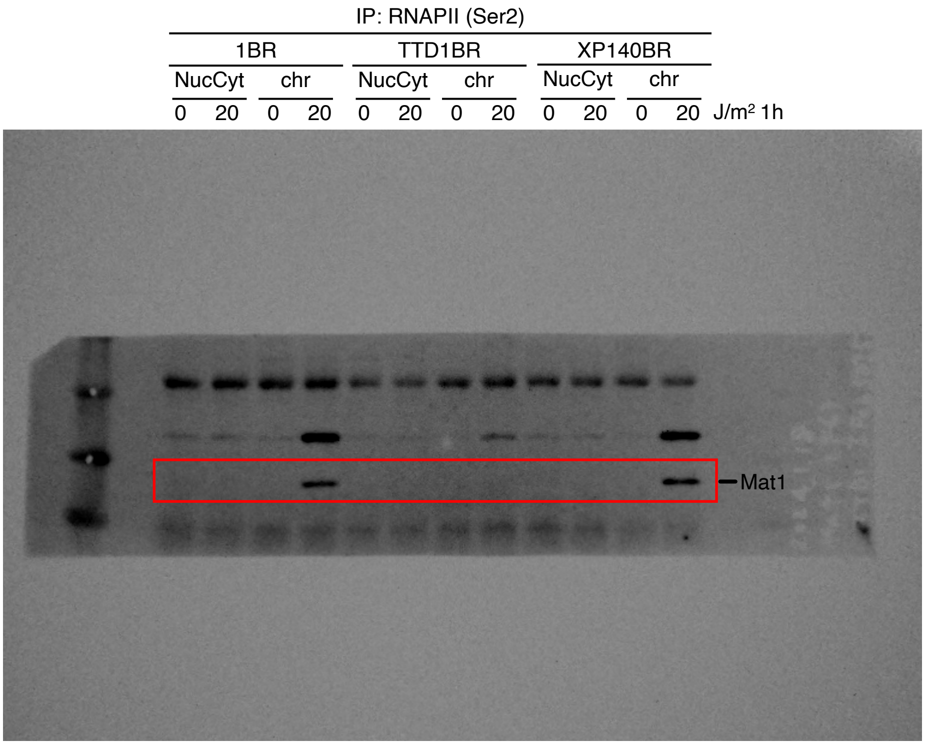
IP: RNAPII (Ser2)
1BR
TTD1BR
XP140BR
NucCyt
chr
NucCyt
chr
NucCyt
chr
0 20 0 20 0 20 0 20 0 20 0 20 J/m2 1h
Mat1

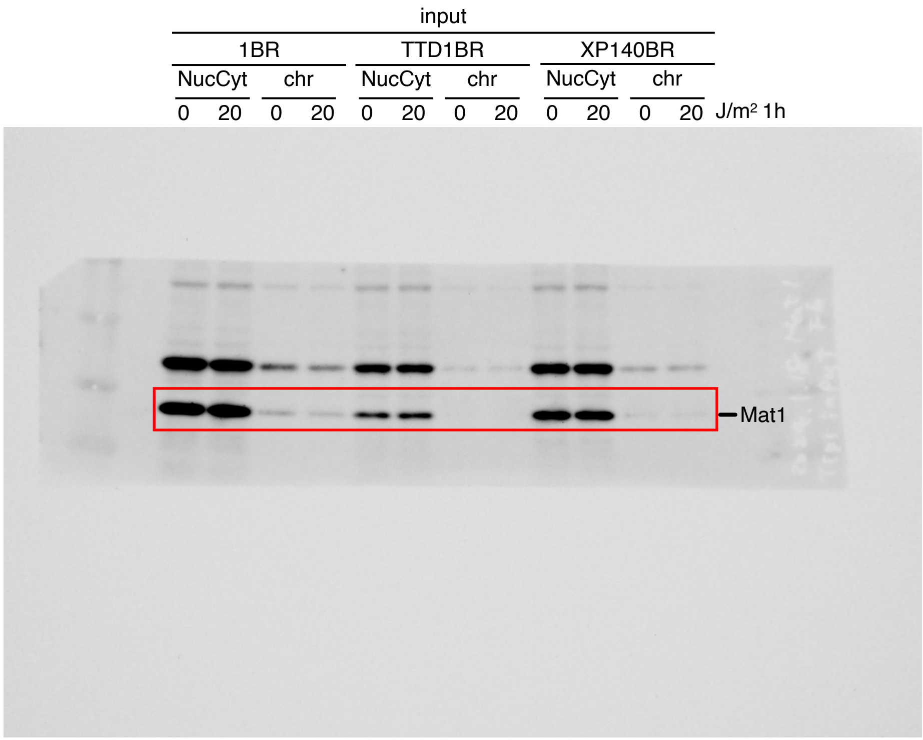
input
1BR
TTD1BR
XP140BR
NucCyt
chr
NucCyt
chr
NucCyt
chr
0 20 0 20 0 20 0 20 0 20 0 20 J/m2 1h
Mat1

Fig3 e

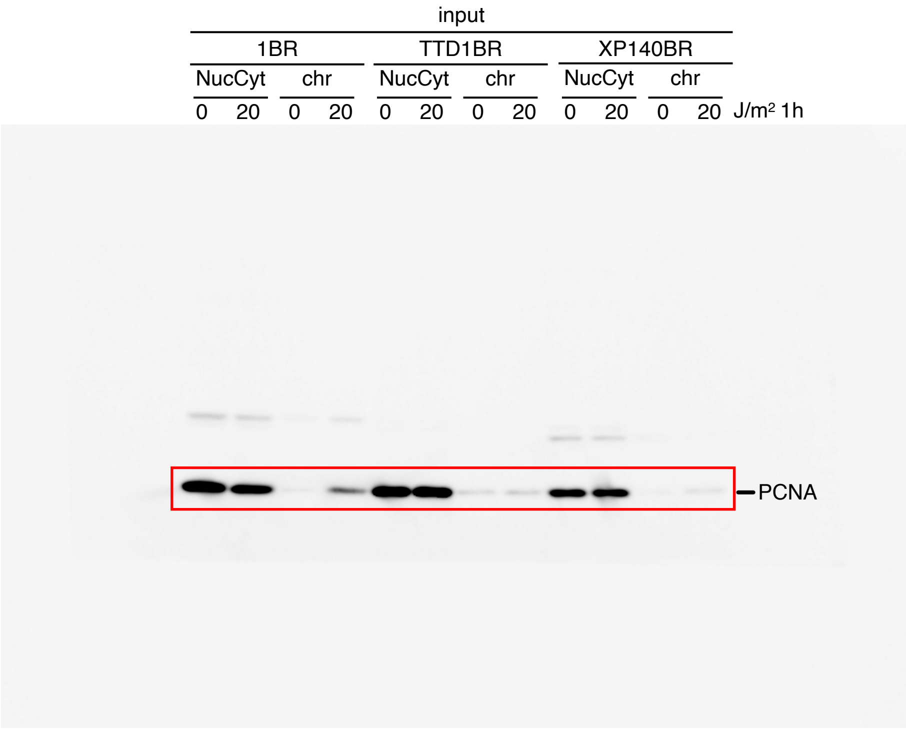
input
1BR
TTD1BR
XP140BR
NucCyt
chr
NucCyt
chr
NucCyt
chr
0 20 0 20 0 20 0 20 0 20 0 20 J/m2 1h
PCNA

Fig4 b

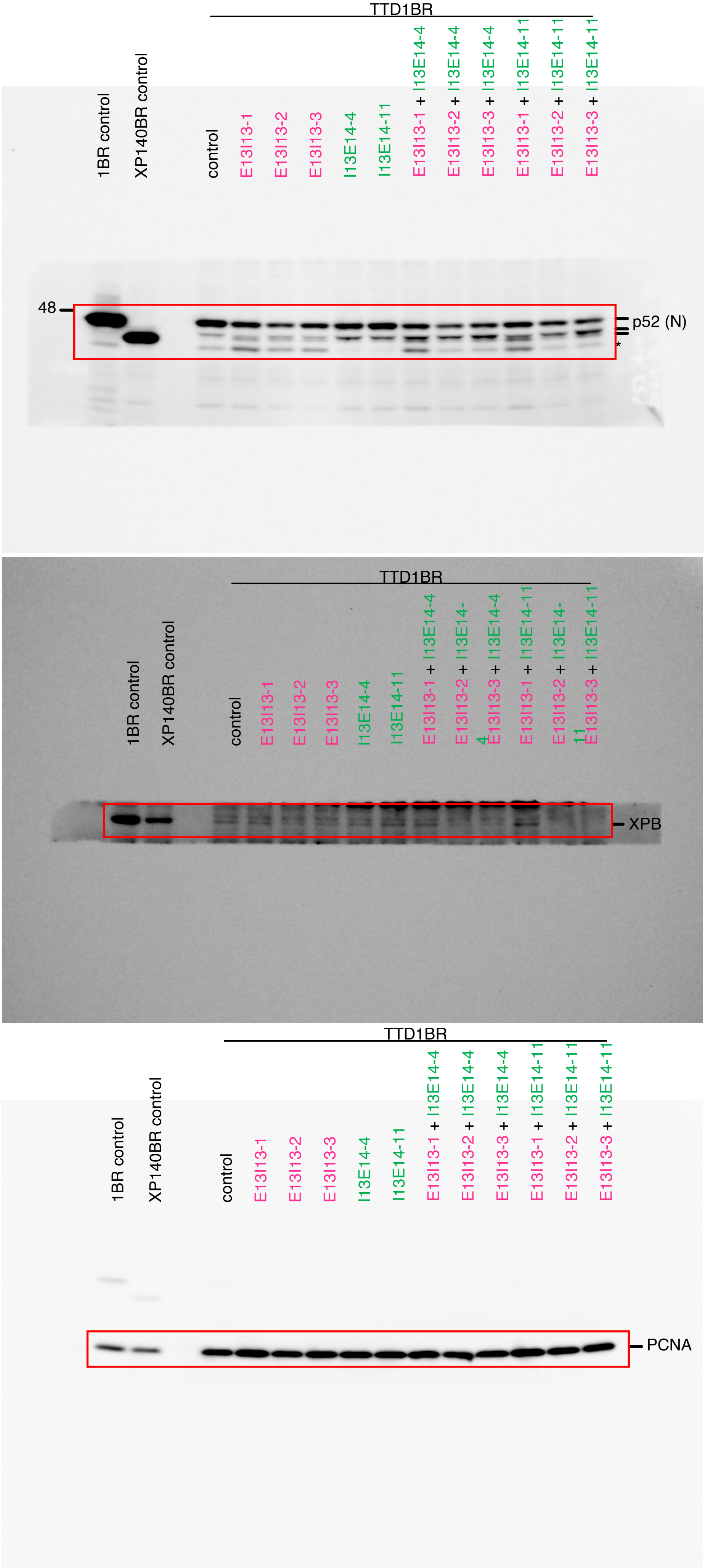
TTD1BR
1BR control
XP140BR control
control
E13I13-1
E13I13-2
E13I13-3
I13E14-4
I13E14-11
E13I13-1 + I13E14-4
E13I13-2 + I13E14-4
E13I13-3 + I13E14-4
E13I13-1 + I13E14-11
E13I13-2 + I13E14-11
E13I13-3 + I13E14-11
48
p52 (N)
*
XPB
PCNA

Fig4 c

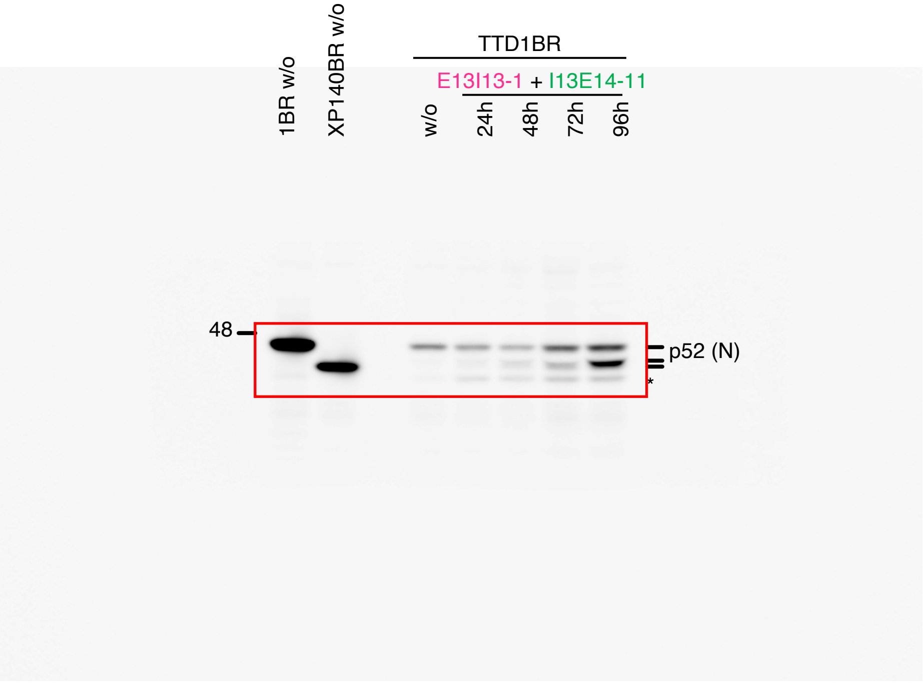
1BR w/o
XP140BR w/o
TTD1BR
E13I13-1 + I13E14-11
w/o
24h
48h
72h
96h
48
p52 (N)
*

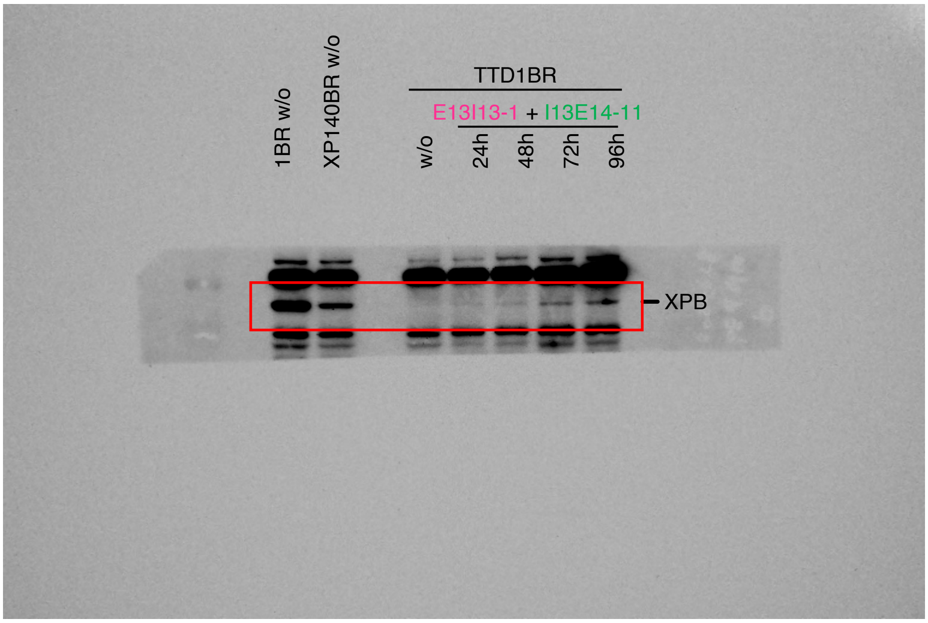
1BR w/o
XP140BR w/o
TTD1BR
E13I13-1 + I13E14-11
w/o
24h
48h
72h
96h
XPB

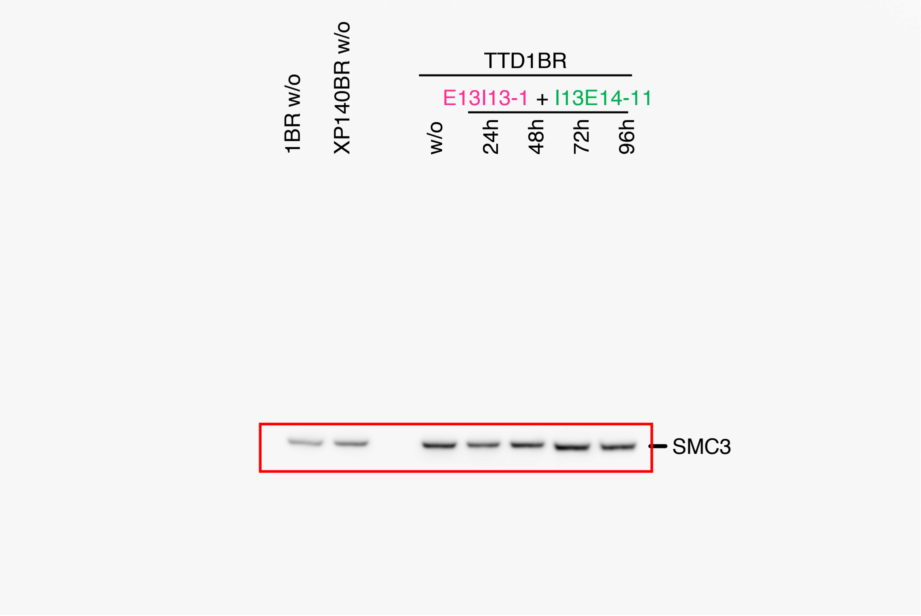
1BR w/o
XP140BR w/o
TTD1BR
E13I13-1 + I13E14-11
w/o
24h
48h
72h
96h
SMC3

Fig4 d

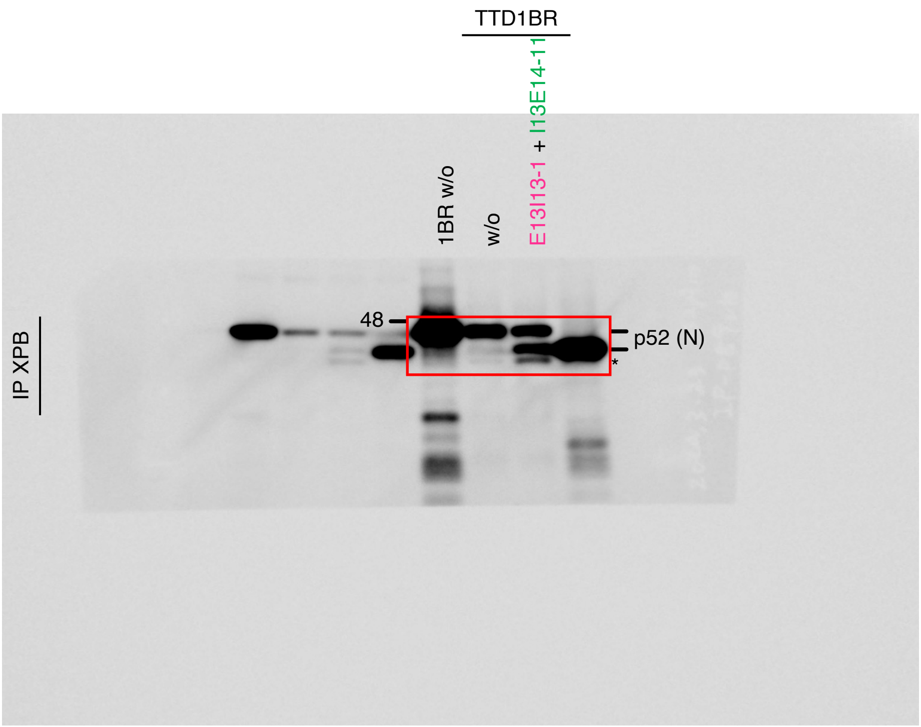

TTD1BR
1BR w/o
w/o
E13I13-1 + I13E14-11
IP XPB
48
p52 (N)
*

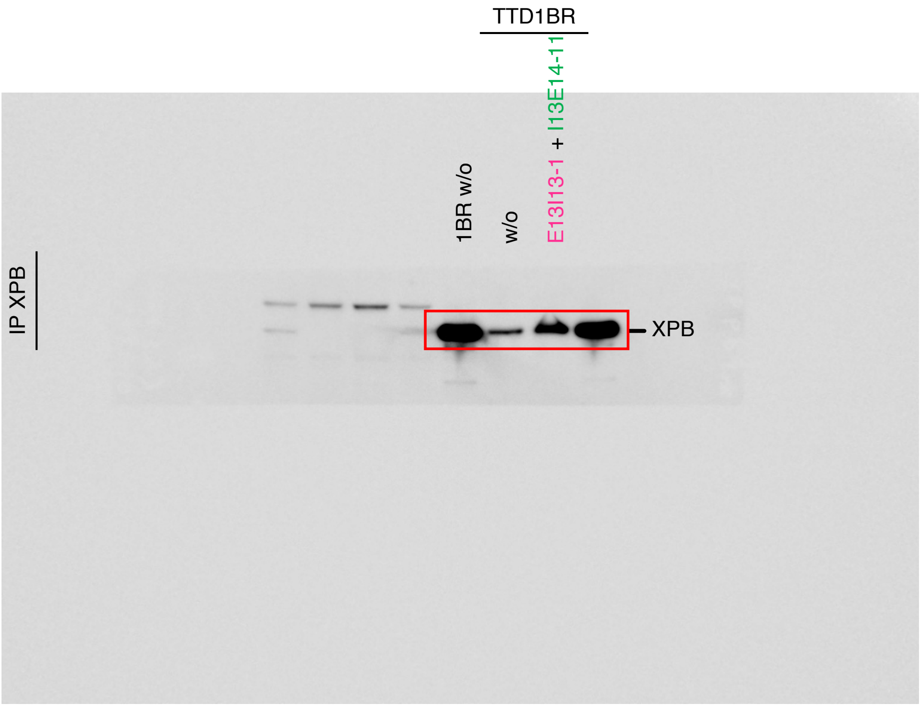

TTD1BR
1BR w/o
w/o
E13I13-1 + I13E14-11
IP XPB
XPB

Fig4 d

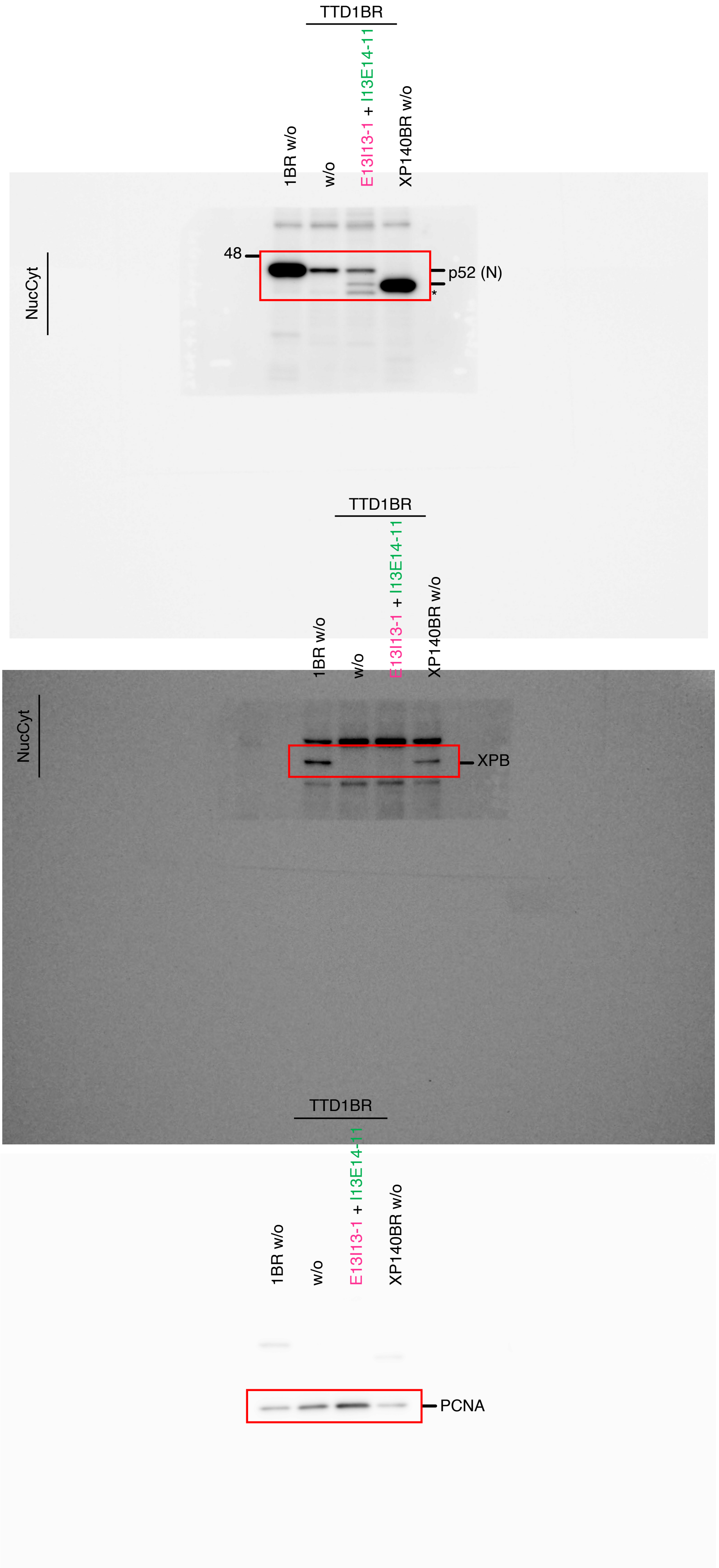
TTD1BR
1BR w/o
w/o
E13I13-1 + I13E14-11
XP140BR w/o
NucCyt
48
p52 (N)
*
TTD1BR
1BR w/o
w/o
E13I13-1 + I13E14-11
XP140BR w/o
NucCyt
XPB
TTD1BR
1BR w/o
w/o
E13I13-1 + I13E14-11
XP140BR w/o
PCNA
